# REVIEW: Towards a risk register for natural capital

**DOI:** 10.1111/1365-2664.12431

**Published:** 2015-04-13

**Authors:** Georgina M. Mace, Rosemary S. Hails, Philip Cryle, Julian Harlow, Stewart J. Clarke

**Affiliations:** ^1^ Centre for Biodiversity and Environment Research Department of Genetics, Evolution and Environment University College London Gower Street London WC1E 6BT UK; ^2^ Centre for Ecology and Hydrology Maclean Building Crowmarsh Gifford Wallingford Oxon. OX10 8BB UK; ^3^ Eftec Economics for the Environment Consultancy Ltd 73‐75 Mortimer Street London W1W 7SQ UK; ^4^ Natural Capital Committee Secretariat DEFRA Area 1B Nobel House London SW1P 3JR UK; ^5^ Natural England Unex House Bourges Boulevard Peterborough PE1 1NG UK; ^6^ The National Trust Westley Bottom Bury St. Edmunds Suffolk IP33 3WD UK

**Keywords:** ecosystem services, goods and benefits, limits, natural capital, targets, thresholds

## Abstract

1. Natural capital is essential for goods and services on which people depend. Yet pressures on the environment mean that natural capital assets are continuing to decline and degrade, putting such benefits at risk. Systematic monitoring of natural assets is a major challenge that could be both unaffordable and unmanageable without a way to focus efforts. Here we introduce a simple approach, based on the commonly used management tool of a risk register, to highlight natural assets whose condition places benefits at risk.
2. We undertake a preliminary assessment using a risk register for natural capital assets in the UK based solely on existing information. The status and trends of natural capital assets are assessed using asset–benefit relationships for ten kinds of benefits (food, fibre (timber), energy, aesthetics, freshwater (quality), recreation, clean air, wildlife, hazard protection and equable climate) across eight broad habitat types in the UK based on three dimensions of natural capital within each of the habitat types (quality, quantity and spatial configuration). We estimate the status and trends of benefits relative to societal targets using existing regulatory limits and policy commitments, and allocate scores of high, medium or low risk to asset–benefit relationships that are both subject to management and of concern.
3. The risk register approach reveals substantial gaps in knowledge about asset–benefit relationships which limit the scope and rigour of the assessment (especially for marine and urban habitats). Nevertheless, we find strong indications that certain assets (in freshwater, mountain, moors and heathland habitats) are at high risk in relation to their ability to sustain certain benefits (especially freshwater, wildlife and climate regulation).
4. *Synthesis and applications*. With directed data gathering, especially to monitor trends, improve metrics related to asset–benefit relationships, and improve understanding of nonlinearities and thresholds, the natural capital risk register could provide a useful tool. If updated regularly, it could direct monitoring efforts, focus research and protect and manage those natural assets where benefits are at highest risk.

## Abstract 2

With directed data gathering, especially to monitor trends, improve metrics related to asset–benefit relationships, and improve understanding of nonlinearities and thresholds, the natural capital risk register could provide a useful tool. If updated regularly, it could direct monitoring efforts, focus research and protect and manage those natural assets where benefits are at highest risk.

## Introduction

The many consequences of environmental degradation have been the concern of ecologists and environmental scientists for decades (Arrow *et al*. 1995). But the rate, nature and extent of environmental degradation has now come into sharper focus with the wider realization that many goods and services that people take for granted depend on the natural environment (Millennium Ecosystem Assessment 2005). The role of the environment underpinning sustainable development was a key topic at Rio+20, the United Nations Conference on Sustainable Development in 2012 where, among other things, governments made commitments to mainstream sustainable development at all levels, integrating economic, social and environmental aspects and recognizing their interlinkages.

The UK Government sponsored National Ecosystem Assessment (UKNEA 2011) was followed by the Government White Paper, ‘The Natural Choice: Securing the Value of Nature’ in 2011. This committed the government to halting the decline of natural capital and to being the ‘first generation to leave the natural environment of England in a better state than it inherited’. The White Paper's stated intention is to ‘put natural capital at the centre of economic thinking and at the heart of the way we measure economic progress nationally’. In England, an independent Natural Capital Committee (NCC) was created to advise on the work. Other countries in the UK are taking different approaches, for example with the development of a Natural Capital Asset Index for Scotland and the creation of an integrated body, Natural Resources Wales, in Wales. Elsewhere, countries are developing approaches to natural capital accounting, including ecosystem mapping in Europe (Maes *et al*. 2013), and accounting frameworks being developed by the UN Statistical Division System for Experimental Ecosystem Accounting (SEEA) (United Nations Statistical Division 2013), WAVES (Defra 2005), and in the UNU‐UNEP Inclusive Wealth Report (UNU‐IHDP & UNEP 2012).

The first of the Terms of Reference for the NCC for England specifies its role to, ‘provide advice on when, where and how natural assets are being used unsustainably. For example, in a way that takes us beyond some acceptability limits or non‐linearity thresholds, or in a way that diminishes some measure of comprehensive wealth’. In this paper, we present the preliminary work that the NCC has developed for identifying the natural assets that are most at risk from unsustainable use, especially bearing in mind the requirement in the Terms of Reference to consider ‘acceptability limits’. This should in turn highlight priorities for action (another of the NCC Terms of Reference).

Natural capital means, literally, capital from nature. The term ‘capital’ comes from neoclassical economics and defines a stock (which may be organized into classes called assets) that has the power of producing further goods (or utilities) to benefit human societies (Ekins *et al*. 2003). At least three major types of capital are usually recognized: natural, human, and manufactured or produced capital. National accounts report mainly on the marketable flows of value from those stocks of capital (roads, buildings, machines, as well as knowledge and skills) using indicators such as gross domestic product (GDP), but not on the capital stocks themselves. Human capital (health, knowledge, culture and institutions) reflects individual and institutional capacity from which an innovative, productive and resilient society results, the value of which is only partially reflected in national accounting aggregate metrics such as GDP. In economic terms therefore, nature (natural capital) can be considered as yielding productive inputs which, when combined with produced and human inputs, generate goods that provide benefits of value to society (Edens & Hein 2013). Methodological approaches are emerging from diverse fields in economics (Arrow *et al*. 2012), accounting (Mayer 2013), and from increasingly well‐developed metrics and analytical models for valuing ecosystem services (Daily *et al*. 2009; Bateman *et al*. 2011). However, methods for accounting for natural capital as a stock, including the thresholds that are characteristic of renewable resources, and the linkages to the economy, development and growth are currently underdeveloped (Helm 2014).

The reference to thresholds in the NCC Terms of Reference reflects a growing appreciation that many natural systems exhibit nonlinear dynamics, whereby small changes in pressures can lead to large, persistent changes in the structure and function of ecological systems, which may then be difficult, slow or impossible to reverse (Scheffer *et al*. 2001; Folke *et al*. 2004). These ecological thresholds arise from the inherent dynamics of natural systems and are distinct from what is meant by the acceptability limits in the NCC Terms of Reference, which indicate the levels of natural capital needed or desired by society. Policies are often adopted to reflect these acceptability limits, either by setting ‘targets’ for maintenance or restoration of aspects of natural capital, or by establishing precautionary limits. Such regulatory limits, enshrined in legislation and policies, are presumably set to reflect people's needs and desires. They are not the same thing as ecological thresholds, although they have important relationships to each other (Johnson 2013), especially in the context of natural capital.

Defining natural capital, delineating the assets, determining the limits of acceptability, incorporating ecological thresholds and identifying the assets at highest risk all pose substantial challenges scientifically as well as in terms of data and information. Even in England, where there is unusually good knowledge about the environment, land and sea use, natural resources and ecosystem science, there is little organized information on which to draw.

A risk register is a tool commonly used by organizations to identify the highest risks to business operations. It highlights those risks that require attention and is developed by considering each plausible risk and other information such as its probability, likely impacts, mitigation potential and where responsibility lies within the organization. Risk registers can be compiled in the absence of full knowledge of the system. The UK Government, for example, publishes annually a National Risk Register of Civil Emergencies. While quantitative approaches are clearly preferable, even qualitative scores based on expert opinion can provide invaluable indications of areas of pending risk (UK Government 2013).

Here we outline methods and present some preliminary results from efforts to identify natural capital assets at risk of unsustainable use. We present a framework for analysing risks and show how it might be applied at national level.

## Methods to identify natural capital assets at risk of unsustainable use

### A framework for the risk register

#### Natural capital

The starting point for a framework needs to be an operational definition of natural capital. Natural capital has been closely equated with ecosystem services (e.g. Kareiva *et al*. 2011), with ecosystems (Dasgupta 2010) and with biodiversity (TEEB 2010), but here we treat it as a stock that includes all natural resources in air, water, sea, land and below‐ground that support human societies. Crucially, it also includes the physical, biological and chemical processes (e.g. weathering, the water cycle, evolution, nutrient cycling, recruitment and ecological interactions). Accordingly, natural capital includes biotic and abiotic elements (as opposed to only biodiversity) and these need not be interacting, as is implicit in the definition of an ecosystem. We reflect Barbier's (2013) description of natural capital as an economy's environment and natural resource endowment.

Natural capital is distinguished from other capitals because it is freely available and can be self‐regulating and self‐renewing without human intervention of any kind. While many of the benefits that flow from it can be augmented with technology, they can rarely be completely replaced, and such endeavours are very often expensive, difficult to sustain or carry side effects that are themselves costly to deal with (Fitter 2013). However, for natural capital to contribute to welfare and production, there is almost always the need for some input of human and produced capital. For example, while soils, water and ecological interactions come together to provide crops, in practice machinery, technology and other inputs are required to sustain the levels of food production needed by society. Natural capital therefore includes all elements of the natural environment that provide benefits to people now and in future. Certain kinds of natural capital require management simply to avoid harm (e.g. pathogens or flood water). Some elements of natural capital are not subject to anthropogenic influence (whether intended or not) even if they affect human welfare and so are excluded on pragmatic grounds. Therefore, natural capital components included in the risk register have the following characteristics: they (i) are changing or likely to change at measurable rates over policy‐relevant time‐scales (decades); (ii) have some actual or potential relevance to human welfare, now or in the future; and (iii) are plausibly subject to management by people in some way to restore or recover, or to restrict use to non‐significant rates of loss, or for use by future generations. These criteria lead to some exclusions from the list of natural assets. For example, while the sun provides energy and every part of life on earth is dependent on it, it is deteriorating at a rate that is barely measurable, and there is nothing that can be done to recover or restore it. Similarly, while mountains provide many sources of value including recreation, aesthetics and microclimates, they are not changing at a rate that materially affects human well‐being and their structure cannot be restored or managed (although mountain ecosystems can be). A final example is clouds which are part of the atmosphere and have measurable benefits (rain, controlling insolation). They might perhaps be managed (e.g. cloud seeding is occasionally practised), but cloud forms and processes change too fast to be meaningful for policy, at least at the moment.

With these issues in mind, we define Natural Capital as: ‘The elements of nature that directly and indirectly produce value or benefits to people, including ecosystems, species, freshwater, land, minerals, the air and oceans, as well as natural processes and functions’ (Natural Capital Committee 2014).

#### Natural capital assets

For the risk register, we need to define categories for natural capital. These are the assets defined by grouping natural capital components according to their biophysical features, the types of benefits they provide and the management they are subject to, in order to ensure the flow of benefits. We define the natural capital assets as: species (including genetic variation), ecological communities, soils, freshwaters, land, minerals, the atmosphere, subsoil assets, coasts, oceans, as well as the natural processes and functions that underpin their operation.

#### Services, goods and benefits

**Figure 1:**
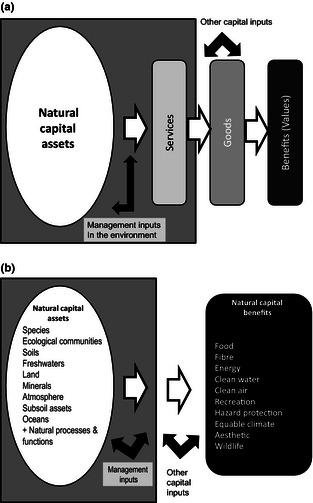
(a) Conceptual framework for the risk register. The natural capital assets in the environment (shaded area) on the left are managed for a flow of services. With the addition of inputs from human and produced capital, these services are transformed to goods, and these goods deliver benefits to people (health, income, enjoyment). The value of different benefits depends on time, place and context. (b) Simplified framework used for compiling the natural capital risk register showing the full set of assets and benefits.

The links between natural capital and the benefits to people underlie the development of metrics. In common with the approach taken in the UK National Ecosystem Assessment (UKNEA) (Bateman *et al*. 2011; Mace & Bateman 2011), we recognize that there is a set of natural capital *stocks* (the assets) (e.g. land used as woodland, soil, species) and each of these may, with appropriate management, provide one or more *services*; these are outputs of each stock in the environment (e.g. freshwater, crops, trees, wildlife). The *services* usually have to be combined with other capital inputs in order to produce *goods*. Goods are ‘good’ things that people receive and use from natural capital stocks (e.g. timber, food, wildlife conservation). Goods need not be physical (e.g. good air quality or recreation) and are consumed or used to provide *benefits* (to people) which can be *valued* (often, but not necessarily in monetary terms). Natural capital stocks provide many potential services and goods from which people derive value. These relationships may change over time and place. For example, the extent to which someone might value a glass of freshwater changes with their circumstances and a similar logic applies to many ecosystem goods and their beneficiaries at a range of scales. Here we are concerned with benefits in an aggregate sense, but recognize that the substantial variation among different groups of beneficiaries, over time, place and circumstance is an important aspect that cannot be ignored in policymaking (Daw *et al*. 2011; Wegner & Pascual 2011). Figure 1a summarizes this framework. For the purpose of developing the risk register, we simplify this chain to only include the natural assets and the benefits shown in Fig. 1b.

We categorize the benefits into major classes: food, fibre (principally timber), energy, freshwater (quantity), aesthetics, recreation, clean air, wildlife, hazard protection and equable climate. The risk register then includes risks to this set of benefits (on the right‐hand side of Fig. 1b) that will result from the degradation of the natural assets (on the left‐hand side of Fig. 1b).

Figure 1 shows that biodiversity may be both an asset (e.g. species, ecological communities) and a benefit (e.g. wildlife). We include species and ecological communities as assets because of the irreplaceable underpinning roles played by the biota in many ecosystem services (Naeem *et al*. 2009; Cardinale *et al*. 2012). However, wild species and habitats are also benefits in the sense that people value wildlife and wild places for education, inspiration, recreation and aesthetic purposes. We use the term ‘wildlife’ for species and ecological community benefits that are often the focus of conservation programmes, recognizing that these are distinct from those that support ecosystem processes and services (Mace, Norris & Fitter 2012).

#### Relationships between assets and benefits

**Figure 2:**
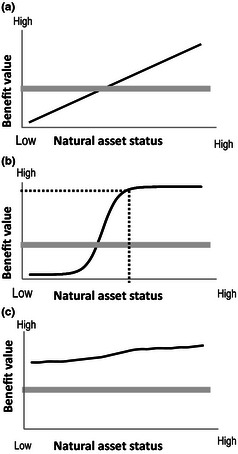
Alternative forms of asset–benefit relationships. The thick grey line in each case represents the target level for benefit. This is a safe level of the natural asset required by society. Environmental degradation may lead to a decline in the status of natural assets, with asset status moving from good to less good condition shown by the solid black line, moving from right to left on the x axis. In (a) and (b), the asset status falls below the target level, but the nonlinear form of the relationship in (b) means that a threshold [shown by the dotted line in (b)] is crossed and the decline in benefits is very rapid at this point. In (c), although the asset status is declining slowly, it is always above the target level required.

Degradation of natural capital is recorded in the risk register in relation to the extent to which it will lead to loss of well‐being in present or future generations. Therefore, while there are many good reasons to monitor and manage natural capital in its own right, here we are interested in declines that may measurably affect human society. In order to do this, we consider the form of the relationship between the condition of a natural asset and any of its benefits (Fig. 2). We define natural asset status according to metrics that best represent the flow of benefits. For example, for food production, this might be the productive biomass (e.g. crop yield) of agricultural land; for water quantity, it might be the flood regulating properties of the catchment; and for wildlife, it could be the diversity of species. As pressures on the environment increase and the status of the natural asset declines, the benefits to people will be reduced. The rate and predictability of this decline is central for the risk register.

#### Targets and safe limits

Figure 2 illustrates alternative hypothetical asset–benefit relationships showing some plausible forms of the relationship between the condition of the natural asset and the benefit provided to people. In addition, a target level of benefit is indicated; this is the benefit required by society (some have already been established as policy objectives). The target is assumed to be determined by present and future needs and would in practice include a precautionary margin for safety (e.g. a safe limit). The targets are similar to the boundaries in the planetary boundaries concept (Steffen *et al*.^2015^), indicating where degradation that presents risks to humanity begins. In order to achieve a level which is both ‘safe and just’, the limits may need to be set with even greater precaution (Dearing *et al*. 2014).

In Fig. 2a, the benefit declines linearly with the asset status; in Fig. 2b, there is a nonlinear decline, and the threshold leads to benefits being rapidly lost (and maybe difficult to recover) below a certain asset status. In Fig. 2c, although the asset status is declining, the benefits lost are very minor and, over foreseeable asset status conditions, never approach a level which would be considered ‘too low’ relative to the target. From Fig. 2, it is clear that knowledge of the form of these relationships, an understanding of how close the delivery of the benefit is to any target, and how close the asset status is to any putative threshold is critical to compiling the risk register.

#### Measuring the status of natural assets

We identify three dimensions of asset status that help resolve how much benefits are affected by deterioration in the condition of natural assets. These measure the quantity, quality and spatial configuration of the assets in relation to the benefits. (i) *Quantity* refers to the ‘amount’ of the asset, its area, volume or mass. Quantity is often relevant for provision of goods (e.g. food from biomass, freshwater from water bodies, energy from biomass), but may also be relevant for some regulatory benefits (e.g. flood control from coastal wetland area, wind or noise regulation from trees). (ii) *Quality* refers to a range of more specific conditions of the natural asset and will be critical where the nature of habitat management or the presence of certain components or processes affects benefits. For example, for wildlife conservation, the quality of habitats may often be more significant than the total area of habitat (Lawton *et al*. 2010) and for flood regulation, both upland‐ and lowland‐habitat land management are important although the relationship is complex (Defra 2005; O'Connell *et al*. 2007). (iii) Spatial configuration refers to the location of the asset and/or its spatial patterning and fragmentation, both of which have been shown to have substantial effects on benefits (Fisher *et al*. 2008; Bateman *et al*. 2011, 2013). Spatial configuration is critical for wildlife and for recreational and health benefits from the environment, but also influences many productive and regulatory functions provided by natural assets, meaning that spatial planning has very substantial consequences for economic decision‐making (Bateman *et al*. 2013).

Metrics for benefits should ideally reflect their contribution to human well‐being and should be net of input from productive or human capital. The values are most often expressed in monetary terms although for wildlife benefits, there are questions about whether it is possible to generate robust estimates at all (Bateman *et al*. 2013). For the purposes of this work, no new primary valuation work was undertaken. Instead values were sourced and transferred from a range of existing studies and from the literature where available and appropriate. This was sufficient for the purposes of this preliminary exercise (see Table S1 and S2, Supporting information).

### Compiling the risk register

#### Natural asset classes

In practice, the information necessary to relate benefits to natural capital asset classes is difficult to find and to organize. The way in which natural capital assets come together to support the provision of benefits is often very complex. For example, food is the product of soils, land, water, species and ecological communities (e.g. pollination and pest control) as well as other types of capital inputs (e.g. labour, additives, machinery, transport). Natural capital provides multiple values that are interdependent and interacting in ways that are complicated to describe. In addition, data on natural capital assets themselves are often lacking even in a well‐monitored and studied environment like the UK (Natural Capital Committee 2014).

Instead, as a means to link natural capital assets to benefits, we use the eight broad habitat types (hereafter referred to as ‘habitats’) identified and mapped in the UK (UKNEA 2011) (see Table 1). These are terrestrial, freshwater and marine habitats and currently are better resolved for terrestrial areas although work is underway to distinguish marine areas. Baseline data from the UKNEA and related sources provide quantitative evidence, whereby the habitats may be linked to natural capital assets and to benefits (UKNEA 2011). The habitats are spatially distinct and they sum to the total land and sea area. They have some parallels with what many ecosystem service assessments refer to as ‘ecosystems’ (Hein *et al*. 2006; Nelson *et al*. 2009), that is they are spatially defined areas of the landscape such as wetland or a river catchment, where multiple ecosystem services can be mapped and measured. The UN SEEA (United Nations Statistical Division 2013) also use habitat classifications as accounting units for natural capital. They define areas within which there is some degree of substitutability in the manner in which they provide services or natural capital inputs. Thus, there is expected to be rather little substitutability *between* areas classified as ‘freshwaters’ and areas classified as woodlands, but there should be substantially more (but certainly not complete) substitutability *among* areas classified as woodlands; this substitutability will be more relevant to the delivery of some benefits than others. While not an adequate representation of natural capital, the broad habitat types provide a starting point for current purposes.

**Table 1:** Classification of broad habitats used for the risk register based on the classification used in the UK National Ecosystem Assessment. For some analyses, these habitat classifications may be too broad and so have been subdivided into meaningful habitat units

Broad habitat type	Component habitats	Scope
Mountains, moorlands and heaths	Blanket bog	Rainfall‐fed bog in upland environments
	Mountains, moorlands and upland heaths	Upland heath, montane habitats and associated wetlands (flushes, fens). Also includes rock and scree habitats such as limestone pavements
	Lowland heath	Lowland habitats dominated by heather family or dwarf gorse species
Semi‐natural grasslands	Semi‐natural grasslands	All grasslands unimproved for agricultural purposes. This includes a range of grassland types
Enclosed farmland	Enclosed farmland	Arable, horticultural land and improved grassland as well as associated boundary features, for example hedgerows
Woodlands	Woodlands	Includes broadleaved and coniferous woodlands both natural woods and planted (wet woodland is included here)
Freshwaters	Standing open waters	Lakes and ponds (reservoirs and canals are considered to be manmade and therefore out of scope)
	Rivers and streams	Streams and rivers down to the tidal limit
	Groundwaters	Aquifers and significant quantities of below‐ground water
	Wetlands	Lowland fens, raised bogs, swamps, reedbeds and floodplain wetlands
Urban	Urban	The natural environment elements of built up areas, for example parks, gardens, towpaths, urban trees, sustainable urban drainage systems
Coastal margins	Coastal dunes and sandy shores	Dune systems and the upper zone of sandy shores
	Saltmarsh	The upper zone of vegetated intertidal habitat – transition into other intertidal habitats
	Transitional and coastal waters	Estuaries, coastal lagoons and other near‐shore waters
Marine^a^	Intertidal rock	Bedrock, boulders and cobbles which occur in the intertidal zone – colonized by mussels/barnacles and seaweeds depending on exposure
	Intertidal sediment	Shingle (mobile cobbles and pebbles), gravel, sand and mud in the intertidal zone
	Subtidal rock	Bedrock, boulders and cobbles in the subtidal zone colonized by seaweeds (infralittoral zone) or animal communities (circalittoral zone)
	Shallow subtidal sediment	Shingle (mobile cobbles and pebbles), gravel, sand and mud in the subtidal zone
	Deep sea bed	The sea bed beyond the continental shelf break
	Pelagic water column	The water column of shallow or deep sea; beyond the coastal waters

a The marine ‘land‐use type’ is based on EUNIS habitat classification and proposals for Marine Strategy Framework Directive reporting. These could be amalgamated to give: intertidal, subtidal, deep sea bed and pelagic.

Different characteristics of each habitat determine the benefits. These characteristics can be evaluated relative to each benefit using the extent or amount of each habitat (quantity), its condition (quality) and where it is (spatial configuration) (Table 1). For example, the more woodland there is, the more timber and wood is likely to be available for harvest (quantity). However, the timber yield of woodlands is very dependent upon management, so the condition and composition of woodlands matters too (quality). Finally, if the recreation benefits associated with woodlands are considered, it matters where the woodlands are in relation to where people live (spatial configuration).

#### Asset–benefit relationships

We considered the relationship between these three characteristics (quantity, quality and spatial configuration) for each of the eight habitats and each of the ten benefits. In total, 240 relationships (3 × 8 × 10) were reviewed (Table S1). The priority relationships were determined to be those where society can, or does, have influence (e.g. we can realistically control conditions to influence the level of benefits) and where the level of benefits involved is non‐trivial. For example, water quality (clean water) is strongly affected by management of enclosed farmland (pollution by nutrients, pesticides and sediment; water abstraction), and as over 70% of England is farmland, it has an important influence over the overall amount of clean water available. Similarly, recreation is strongly affected by the quantity and location of woodlands, but the quality in terms of species composition is less important (although tracks allowing internal access will be important). Conversely, the quality of freshwater habitat types strongly affects recreational benefits; quantity and spatial configuration much less so (see Table S1 for details).

#### Establishing targets and acceptability limits

Identifying the target or limit for each asset–benefit relationship (Fig. 2) is a key step in developing the risk register. The limit defines the point at which change in the asset becomes deleterious or even dangerous, that is it defines the nature and severity of the risk. Although many commentators equate dangerous change simply with thresholds in the asset themselves, this can lead to failures to spot losses of benefits where there are no thresholds, or indeed to recover assets that have no societal consequences. As is clear from Fig. 2, target levels for the asset cannot be established in the absence of societal aspirations, nor without understanding the form of the asset–benefit relationship. Here we take a range of approaches to identifying limits of acceptability, societally relevant targets, and to characterizing the form of the asset–benefit relationship with respect to the target level (Fig. 2).

We used the most relevant policy target in each case where it exists. In many instances, this relates directly to indicators of status, for example EU Water Framework Directive ecological status classes. For the habitats, the target used varies according to the type of the benefit provided. So for example, in assessing the level of benefits derived from changes in woodland quantity, woodland status has been measured against the government's woodland cover target (12% by 2060). Similarly, for recreation in coastal areas, compliance with the EU Bathing Waters Directive has been used, and for marine fisheries (in the absence of specific targets for different stocks), the target used was an average of fish stock levels between 1938 and 1970. For some habitats, no similar, universal, target for quality exists; in these cases, the condition of Sites of Special Scientific Interest has been used as a proxy across all land in a particular habitat type. This assumption is justified on the basis that, although targets for SSSIs are likely to be more stringent, equally their protected status should mean that there is greater emphasis on securing the right management. Overall, we would expect SSSI land to be in a better state than non‐SSSI land in a similar habitat category, and hence, our assumption is likely to be conservative.

**Figure 3:**
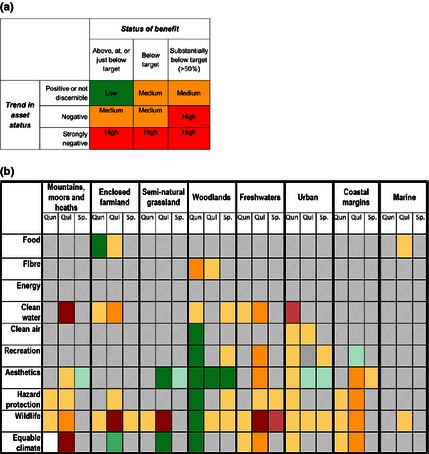
(a) Risk register scoring matrix; (b) The risk register results showing broad habitats as columns, and benefits as rows. The grey cells indicate relationships that were assessed to not be significant or where there is no information from which to make an assessment. Where the relationship is known to the extent that an assessment is possible, the colour of the cell shows the risk rating for the relationship, using the scoring matrix in Fig. 3a. This indicates that the quantity (Qun), quality (Qul) and spatial configuration (Sp.) of the broad habitat type have significant consequence for the benefits; red indicates it is at high risk, orange at medium risk and green at low risk. The density of the colour weakens with more uncertainty (see Table S1 and S2).

The risk register provides a risk classification for each characteristic and habitat type and benefit using the relationships outlined in Fig. 2. The risk assessment matrix (Fig. 3a) shows the risk scoring as high (red), medium (orange) or low (green) based on whether the benefit level is currently above or below target and whether the asset is deteriorating and how rapidly (see Table S2). Each relationship was assessed for the strength of the evidence and for the agreement among assessors, and an uncertainty score was determined for each one (Table S2).

## Results

### The relationship matrix

For each asset–benefit relationship, we determined the form of the relationship between the habitat type and the level of benefit. We first determined whether there was a substantial effect on benefits from each condition in each habitat. In this initial analysis, of these 240 relationships, 167 were judged to be of lower significance for either functional or management reasons (see Table S1); for example, the quantity of the land area covered by the marine environment cannot be altered or managed to enhance or reduce benefits derived from the marine environment. This left 73 relationships for closer scrutiny, and these were all assessed in detail (Fig. 3b).

### The risk register

We considered each of the 73 priority relationships against the risk matrix in Fig. 3a, and using available information on the status and trends of natural assets and benefits (see Table S1 and S2), we allocated each relationship to one of the risk register categories (high, medium or low) (see Fig. 3b).

Seven relationships have been allocated to the high‐risk category (red). These are cases where there is reasonable confidence that the current status of the natural capital assets in the relevant broad habitat types is poor, and/or the trends are strongly negative in the relevant dimension (quantity, quality and spatial configuration). The categories of goods and, therefore, benefits most at risk include the following:

- clean water from mountains, moors and heaths, due to the quality of those habitats;
- clean water from the current extent and projected growth of urban habitats leading to a deterioration in freshwater, soils and natural water purification processes in these areas;
- wildlife is at risk in many habitats (semi‐natural grasslands, enclosed farmland and freshwaters) due to poor‐quality habitats and unfavourable spatial configurations; and
- equable climate, essentially carbon storage, is at risk from the degraded condition of mountains, moors and heaths which have the potential for much greater carbon storage.

The medium‐risk category (orange) includes two‐thirds of the relationships assessed, but for nine of these, a confident assessment was not possible due to lack of information. These ‘unknown’ relationships are included in the orange category as a precautionary measure subject to further analysis. Of those medium‐risk relationships where information on status and/or trends is available, the types of benefits at risk include wildlife and hazard protection, with clean water, aesthetics, equable climate and recreation also featuring prominently. The freshwater and urban land‐use categories have the greatest number of relationships at risk.

Across the two highest risk categories (high and medium), freshwater and mountains, moors and heaths are the two habitats with the greatest number of benefit types at risk, with six and five, respectively. Both provide a range of benefits and are currently subject to a number of human pressures. Hence, it is the quality of these habitats that is reflected in our risk assessment.

The analysis identified 17 relationships which are considered to be at low levels of risk (green) based on current information. These include aesthetic benefits from a range of habitats, particularly in relation to the spatial configuration of those land‐use categories.

Overall, it is the quality of the habitats that is most often the cause of a high‐risk classification, rather than their quantity or the spatial configuration. The analysis also includes a number of relationships for which there was insufficient evidence to assess either status or trend.

## Discussion

### Implications of results

The risk register identifies natural capital assets where further degradation places the benefits we derive from them at risk. We found that the habitats with most benefits at risk were freshwater, and mountains, moors and heath. For freshwater, the benefits at risk are clean water, recreation, aesthetics, hazard protection, wildlife and equable climate. For mountains, moors and heaths, the benefits at risk are aesthetics, hazard protection, wildlife, clean water and equable climate. Here, the high level of risk is largely a result of significant loss and degradation of blanket bog over the last 60 years. Historical air pollution combined with unsuccessful attempts to convert this habitat to productive agricultural land has left a legacy of soil erosion, impoverished vegetation and associated impacts on wildlife, carbon storage and clean water provision. Increasingly, areas of upland blanket bog in the UK are being restored through reversal of drainage schemes and restoration of native peat‐forming vegetation. This work is being driven by both recognition of the problems habitat degradation brings and the benefits that might be delivered through reinstating blanket bog vegetation and function (Bonn *et al*. 2014).

Similarly, freshwaters continue to suffer because, despite many recent conservation successes and action under the EU Water Framework Directives, they continue to be affected by activities in other land‐use categories. Freshwater areas are sinks for sediments and pollutants generated in the landscape and are thus affected by a range of land uses (e.g. agriculture, urban runoff) as well as being intensively managed themselves to provide clean water and to deal with wastewater. The current status of freshwater supports this conclusion with only 33% of water bodies classified as being in good ecological status (or better) in England (Defra 2013).

Of particular note among the low‐risk relationships are those benefits associated with the quantity of woodland, which has doubled in the post‐war period. This positive trend means that benefits associated with the amount of woodland (fibre, clean air, aesthetic benefit, equable climate, recreation, wildlife) are considered to be low risk. However, it is important to note that many of these benefits are still at risk due to the poor quality and unfavourable spatial configuration of this increased woodland. The low‐risk category also includes food provision from the quantity of farmland which reflects the fact that in the past 70 years, most increases in UK food production have come from improvements in other forms of capital (fertilizers, machinery, crop varieties, cropping techniques) rather than by bringing more land into agriculture. Despite the general low‐risk score for food, we noted above that the risk register based in broad habitat types deals rather poorly with highly dispersed assets, such as soil. Soil is a continuing concern in the UK because of additives and pollutants, and it is affected by erosion; around 2.2 million tonnes of topsoil is eroded annually in the UK (Environment Agency 2004).

Our assessment of urban habitats is likely to be rather weak because unlike rural areas where the roles of natural capital are well recognized, there are limited data. In fact, many natural assets in urban areas are both very significant for people and show marked degradation. For example, air and water quality in urban areas, and the significant role of trees and urban green space for regulating and cultural services (Tratalos *et al*. 2007) may have been weakly represented in our analysis as a result of the habitat classification being broad and the key natural assets in urban areas being dispersed.

Our finding that quality and spatial configuration featured more commonly than quantity in the risk register is encouraging, since in theory, these are easier attributes to address than quantity which is ultimately always limited by land and/or sea area. This result may partly be a consequence of using habitats as categories in the risk register and also by data availability. The finding is, however, in line with other assessments which suggest that the condition of many of our natural capital assets is degraded or in decline. For example, Lawton *et al*. (2010) emphasized the importance of improving the condition of protected sites for wildlife as well as increasing their size and improving their connectedness.

An increase in the quantity of one broad habitat type is balanced by a loss in another. In other words, there is a bias towards seeing trade‐offs in benefits in the risk register despite the potential for management to support multiple benefits from land and seascapes (Bennett, Peterson & Gordon 2009; Raudsepp‐Hearne, Peterson & Bennett 2010). We do not assess such trade‐offs, nor is there any way to represent multiple benefits not already recorded. In addition, other analyses have illustrated that there may be significant advantages for society in changing the balance of land use between land‐use categories (Bateman *et al*. 2013).

### Discussion of the approach

This risk register is a new approach compared to most previous analyses, where natural capital has been assessed in its own right. It provides a means of highlighting those benefits derived from natural capital that are at high risk. Despite the lack of relevant data and information, and even in the absence of comprehensive establishment of societal targets, it provides a practical way to guide decisions about the priorities for recovery and protection of natural assets. The conclusions are of course very tentative, but even the process of constructing the risk register, and compiling information indicates significant areas for data gathering and research that could quickly make this a more robust and policy‐relevant tool. We indicate below some of the priority areas for new research and monitoring, but first consider briefly some more fundamental issues about the design of the risk register.

First, we acknowledge that valuation of nature is itself controversial. However, our approach does not rely on monetary valuation. The metrics for benefits can in principle be any indicator that society chooses, and the metrics for assets should simply resolve the relationship most effectively. There are, however, difficulties with any metric for certain assets and benefits. Many authors highlight biodiversity as the most difficult natural asset to value and to assess at a national level, both because of its complexity as a concept and its multiple, complex and subjective values (Mace 2013). We acknowledge all these concerns in the risk register. We follow Mace, Norris & Fitter (2012) in recognizing that different components of biodiversity are both assets and benefits, and we measure each one accordingly.

As described above, we used the broad habitat types from the UKNEA (2011) as proxies for natural capital assets. The main justification is that it provides an almost coherent classification of land uses with similar biophysical components and processes and can therefore deliver similar benefits. In principle, we suggest that the risk register should consist of assets where the biophysical features and potential benefits are commensurate within rather than between categories. The habitats classification achieves this and is consistent with other ecosystem‐based assessments that have used land classifications in this way (e.g. UN 2003). There are, however, several weaknesses in the approach. Marine areas are poorly represented, despite their importance for many benefits. Also, because the habitats by definition add up to the total land and sea area of the UK, they are essentially fixed in the assessment, and any virement of land uses between them will appear to be a trade‐off. This means that the potential for management of habitats for multiple different benefits will be underestimated. Habitat classifications may overemphasize the differences between habitats, which do in fact exist in a continuum on multiple dimensions. Finally, certain types of natural capital that are widely discussed as causes for concern cannot be reflected in this approach. In particular, we highlight soils, the atmosphere and land use.

How might natural capital assets otherwise be classified? As mentioned above, comparable approaches, such as the SEEA, also use land‐use types (United Nations Statistical Division 2013). The current EU project, Mapping and Assessment of Ecosystems and their Services, is based on ecosystem mapping that is broadly equivalent, although it takes a more biodiversity‐based approach to ecosystem services (Maes *et al*. 2013). However, other approaches have also been suggested in discussions of critical natural capital. De Groot *et al*. (2003) distinguished two types, one for maintaining ecosystem functions (ecocentric) and one for human well‐being (anthropocentric). Ekins *et al*. (2003) distinguished four types: production, absorption (waste management), life‐support (= regulating) and amenity, which provides a useful framework that is comparable to, but more inclusive than, classifications commonly used in environmental economic models. There are many other possibilities, but we are most interested in classifications that link key environmental functions to policymaking. This is a key area for further development.

In principle, the risk register includes all natural resources and their benefits: renewables and non‐renewables. In practice, it ended up dominated by renewables, mainly because these are more easily developed using the asset–benefit relationships. However, the UK's initial aggregate estimates of the value of natural capital (ONS 2014) include many non‐renewables, and it is noticeable that many current conflicts in environmental management arise between non‐renewables and renewables. We therefore consider that the risk register needs to be developed to incorporate all natural capital assets, as it may provide a new way to prioritize among such conflicts.

A key area of uncertainty in compiling the risk register concerned the thresholds and nonlinearities in the asset–benefit relationships. While natural capital thresholds are an important area of scientific interest and enquiry, especially as they relate to tipping points and dangerous change (Rockstrom *et al*. 2009; Lenton & Williams 2013), nonlinearities also occur at local scales with potentially important consequences and are a very common feature of ecological systems (Muradian 2001; Raffaelli & White 2013). Our knowledge and understanding of these effects is inadequate, but there is an emerging literature of both theoretical and empirical case studies (Suding & Hobbs 2009; Bullock *et al*. 2011) that will need to be developed. In most cases, our analysis only incorporated such effects phenomenologically, that is where they are represented implicitly in specific asset–benefit relationships (e.g. wild fisheries). This will constitute an important gap in the risk register when an assumption of a linear change is non‐precautionary, and therefore, we consider the focused analysis of thresholds relevant to benefit–asset relationships to be essential for any future application of a risk register.

An initial analysis of the data available for reporting on assets (Natural Capital Committee 2014) indicates that there are potentially good data sets and a partial picture for many of the other assets. Assets subject to existing regulatory regimes, such as the EU Water Framework Directive and the recently adopted EU Marine Strategy Framework Directive, are increasingly monitored in a systematic way, and although coverage may be limited (e.g. exclusion of small water bodies and headwaters), there is an emerging picture of overall status. Other assets (soils, atmosphere) are relatively well monitored for specific purposes but lack composite metrics against which their overall status and trends can be assessed. There are also assets for which only certain components are well monitored giving only a partial picture of their overall status and trends (species, ecological communities).

The shortcomings of existing data are clear from a review of species monitoring information (Table 2). Abundance data are particularly inadequate for many species groups, and there is a clear bias towards charismatic and easily identified taxa (birds, higher plants, butterflies, dragonflies) or those that are commercially valued (some marine fish). Furthermore, most information on species and ecological communities (approximating to habitats) is often focused on those already known to be of concern, with the result that declines in widespread and common species and rare or significant ecological communities (for example bogs and ancient woodlands) could deteriorate without our being aware (Gaston & Fuller 2008). A more detailed assessment of current monitoring data is provided by Maskell *et al*. (2013).

**Table 2:** Summary of current knowledge and data availability for UK species

Location	Species group	Abundance	Distribution	Trend
Terrestrial and freshwater	Micro‐organisms	−	+	−
	Fungi	−	+	−
	Algae	+	+	+
	Lichens	+	+	++
	Bryophytes	+	++	++
	Higher plants	+	++	++
	Invertebrates (freshwater)	++	++	++
	Invertebrates (terrestrial)	+	+	+
	Fish (freshwater)	+	++	+
	Amphibians	−	+	+
	Reptiles	−	+	+
	Birds	++	++	++
	Mammals	+	+	+
Marine	Phytoplankton	−	++	++
	Algae (marine)	+	+	+
	Invertebrates (marine)	+	+	+
	Fish (marine)	++	++	++
	Seabirds	+	+	+
	Mammals (marine)	+	+	+

−, little or no data; +, data inconsistently collected across components, time or space; ++, good data at appropriate spatial or temporal scales.

The targets used in the preliminary risk register were drawn from existing policies only. This has the advantage that they are clearly high priority targets and that there is no question about societal commitments to them. However, a significant drawback is that they are incomplete, maybe set at arbitrary or risk‐prone levels and may not include a safe allowance for uncertainties or lag times in either recording natural capital deterioration, or in implementing policies to reverse declines (limits). Targets may have been set without an appreciation of the economic benefits that may result or the cost of restoration and future maintenance. In addition, these targets have mostly been developed in isolation, considering only one benefit at a time, and the possibility that multiple benefits might alter them in significant ways is difficult to incorporate given that the targets or limits are developed in policy environments that are also relatively isolated. In addition, it is important that targets and limits are set over time‐scales that are relevant for the risk register.

How could targets be established? They might be set through a scientific or technical analysis of the benefit–asset relationship. This can be done most easily in cases where the relationships are direct and reasonably well understood, and target setting is especially important where there are nonlinearities in the system. For example, the carbon emission targets set by the UK Government come from a scientific understanding of the role of greenhouse gases in the atmosphere regulating climate, an assessment of dangerous levels of climate change, and the contribution to global carbon emissions in the UK (Committee on Climate Change 2008). Similarly, levels of offtake of fisheries species are at least informed by a scientific understanding of the functional relationship between offtake and population size (or biomass) allowing the calculation of a maximum sustainable yield, or population viability analysis can indicate the area or configuration of good quality habitat necessary to ensure that a species has a high probability of long‐term persistence (Fryxell, Sinclair & Caughley 2014).

Targets might also be established using standard cost–benefit calculations. For example, if all households in England need assurance that they face some low probability of flood risk (say 1:200), then flood plains and upland water management can be designed to help achieve this. For example, when locating new housing, greater attention may need to be given to avoiding high‐risk flood zones. Similarly, a certain level of air pollution in urban areas might be prescribed that minimizes the longterm health costs for people relative to the required changes to transportation fuel quality and control. Such approaches have commonly been applied in ecosystem valuation (Bateman *et al*. 2011; Barbier 2014).

A third, common means by which targets have been established is through legislation and policies based on societal desires and wishes. Thus, we have a network of National Parks, Areas of Outstanding Natural Beauty, Sites of Special Scientific Interest and a suite of protected sites that arise from EU legislation (EU Birds Directive, EU Habitats Directive). These are all public designations developed to meet people's wishes and expectations to conserve aesthetic and historical aspects for enjoyment of generations to come.

## Conclusions

Natural capital is of central importance to the economy and to people's welfare now and in the future, and yet there is much evidence that many natural assets are declining and deteriorating to such a degree that the benefits are at risk. The task of monitoring and measuring natural capital at a national level is therefore important, but also very challenging given the scale of data gathering and analysis that would be necessary for a comprehensive assessment. The approach described here, using a risk register based on existing knowledge and expert judgement, provides an effective means to highlight the areas of greatest concern and to focus future monitoring and data gathering. Our preliminary natural capital risk register for the UK identifies the main habitats, or land‐use types, where the benefits may be at risk, including freshwater and mountains, moors and heaths. Certain other habitat types, especially urban, coastal and marine are poorly represented due to difficulties in gathering relevant data. Certain benefits seem especially at risk, including freshwater, wildlife and climate regulation. A key finding, that often it is the quality or spatial configuration of natural assets that places the benefits at risk more often than the quantity, is encouraging since better management and spatial planning can then be used to rapidly improve the benefits.

The risk register is preliminary and could be usefully elaborated especially with a more thorough analysis of the quantitative relationships between natural assets and their benefits, as well as consideration of costs of maintenance and/or recovery and the extent to which loss and degradation might be replaced by or compensated for using human, or manufactured or produced capital. This analysis also requires a clear assessment of the societally relevant targets for benefits, without which the asset–benefit relationships cannot be interpreted. In this preliminary assessment, targets were based on existing policies or regulations, which exclude some important targets, but also tend to emphasize the benefits that are most economically significant, and most well understood in the short term. Therefore, work is needed to ensure that the risk register is sufficiently inclusive of more poorly documented or understood benefits at risk.

Another advantage of continuing to manage a risk register, perhaps updating it on a biennial basis, is that it directs data gathering to natural assets and benefits that are the most significant for society, and clearly points to areas where policy mechanisms are needed to address problems and restore the natural assets.

The risk register points to some significant gaps in ecological science. It emphasizes the importance of measuring status and trends in natural assets, at a scale and resolution that is relevant to their benefits. Current monitoring is often focussed on status more than trends and spatial features are under‐represented. Linking the natural assets to sustainable benefits also requires a strong science base concerning the functions and processes of natural systems that lead to nonlinear dynamics affecting the benefits. The thresholds that result can cause unexpectedly severe, and a hard to reverse loss of benefits, and being able to predict thresholds and their consequences is a significant and very important challenge. A stronger understanding, based on relevant ecological studies, will add further value to the risk register and contribute to sustainable benefits from natural capital into the future.

## Supporting information


**Table S1.** Justification for prioritization of functional relationships between each broad habitat type, benefit and characteristic.

**Table S2.** Justification for risk register scoring and references used to support the scoring.
