# Supplementary material for: REVIEW: Towards a risk register for natural capital
Source: J Appl Ecol. 2015 Apr 13;52(3):641–53. doi: 10.1111/1365-2664.12431 (PMC4979659; doi:10.1111/1365-2664.12431)
Supplement: Supplementary file 1 — Table S1. Justification for prioritization of functional relationships between each broad habitat type, benefit and characteristic. [file JPE-52-641-s001.pdf]

Supplementary Table 1:

Justification for prioritization of Functional Relationships between each Broad Habitat type, Benefit and Characteristic.

The 240 potential relationships were reviewed and classified into four groups:

1. Non-prioritised (where the benefit seemed unlikely to be impacted by any characteristic in any Broad Habitat type),
2. Negligible relationships;
3. Minor relationships
4. Prioritised relationships (where the condition of the benefit was substantially affected by the condition of the Broad Habitat type.

The tables below include the results of consideration of all 240 relationships based on 8 Broad Habitat types (Mountains, Moorlands and Heaths (MMH); semi-natural grasslands (SNG); Enclosed farmland (EF); Woodlands (W); Freshwaters (F); Urban (U); Coastal Margins (CM); Marine (M)); 10 Benefits (Food, Fibre, Energy, Clean water, Clean air, Recreation, Hazard protection, Equable climate; Aesthetic; Wildlife); and 3 characteristics (Quantity, Quality; Spatial configuration). Only in the case of the Minor and Prioritised relationships were more details collated on the form of the relationship (Linear (L) or Non-Linear (NL;positive (+) or negative (-).

1. Non-prioritised relationships

No relationship

Habitat type	Benefit	Characteristic	Relationship Justification
MMH	Food	Spatial configuration	No relationship - the spatial configuration of the A/U cannot be influenced by society to change the amount of benefit realised. Location of A/U is defined by altitude - over 300m.
MMH	Fibre	Spatial configuration	No relationship - the spatial configuration of the A/U cannot be influenced by society to change the amount of benefit realised. Location of A/U is defined by altitude - over 300m.
MMH	Energy	Spatial configuration	No relationship - the spatial configuration of the A/U cannot be influenced by society to change the amount of benefit realised. Location of A/U is defined by altitude - over 300m.
MMH	Clean water	Spatial configuration	No relationship - the spatial configuration of the A/U cannot be influenced by society to change the amount of benefit realised. Location of A/U is defined by altitude - over 300m.
MMH	Clean air	Quantity	MMH A/U has no significant relationship in production of clean air benefit.
		Quality	
		Spatial configuration	
MMH	Recreation	Spatial configuration	No relationship - the spatial configuration of the A/U cannot be influenced by society to change the amount of benefit realised. Location of A/U is defined by altitude - over 300m.
MMH	Hazard protection	Spatial configuration	No relationship - the spatial configuration of the A/U cannot be influenced by society to change the amount of benefit realised. Location of A/U is defined by altitude - over 300m.
MMH	Equable climate	Spatial configuration	No relationship - the spatial configuration of the A/U cannot be influenced by society to change the amount of benefit realised. Location of A/U is defined by altitude - over 300m.
EF	Clean air	Quality	No relationship - the quality of the A/U cannot be influenced by society to change the amount of the benefit produced. (Note that effects of methane/nitrogen oxide are considered in equable climate)

Habitat type	Benefit	Characteristic	Relationship Justification
		Spatial configuration	No relationship - the spatial configuration of the A/U cannot be influenced by society to change the amount of the benefit produced.
EF	Recreation	Spatial configuration	No relationship - the spatial configuration of the A/U cannot be influenced by society to change the amount of the benefit produced i.e. the location of EF will not be changed for recreational benefits - other over riding factors regarding location (e.g. best soils for arable crops etc).
EF	Hazard protection	Spatial configuration	No relationship - the spatial configuration of the A/U cannot be influenced by society to significantly change the amount of the benefit produced.
EF	Equable climate	Spatial configuration	No relationship - the spatial configuration of the A/U cannot be influenced by society to change the amount of the benefit produced.
SNG	Energy	Quantity	No relationship. Biofuel production has been included in the Enclosed Farmland A/U.<br>SNG covers 1% of the total area of England (SoNE, 2008) and characterised by grassland that has not been managed for anything other than conservation.
		Quality	
		Spatial configuration	
SNG	Clean water	Quantity	No relationship. SNG does not contribute to the production of clean water. The UK NEA discusses semi-natural grassland as being a better land use that the Enclosed Farmland A/U in terms of water quality.
		Quality	
		Spatial configuration	
SNG	Clean air	Quantity	No relationship. Semi-natural grassland does not contribute to the clean air benefit.
		Quality	
		Spatial configuration	
SNG	Recreation	Spatial configuration	No relationship. Semi-natural grassland is geographically constrained by underlying geology and restricted to 1% of total area of England (SoNE, 2008).
SNG	Hazard	Quantity	No relationship. Semi-natural grassland does not contribute to the protection from hazards and restricted to 1% of total

Habitat type	Benefit	Characteristic	Relationship Justification
	protection	Quality	area of England (SoNE, 2008).
		Spatial configuration	
SNG	Equable climate	Spatial configuration	No relationship - semi-natural grassland is geographically constrained by underlying geology.
W	Fibre	Spatial configuration	No relationship - it does not matter where the woodland is, assuming infrastructure is in place to transport timber to beneficiaries. Currently 80% of the UKs wood and wood product needs are met by imports (UK NEA 2011,pg 262).
W	Energy	Spatial configuration	No relationship - it does not matter where the woodland is, assuming infrastructure is in place to transport timber, for biofuel, to beneficiaries. Currently 80% of the UKs wood and wood product needs are met by imports (UK NEA 2011,pg 262).
W	Clean water	Quality	No relationship- it is not the quality of the A/U that determines the benefit, but quantity and spatial configuration of woodland, and therefore uptake of water and potential to intercept pollutants.
W	Hazard protection	Quality	It is not the quality of the A/U that determines the benefit, but quantity and spatial configuration of woodland, and therefore uptake of water and binding of soils.
W	Equable climate	Spatial configuration	No relationship - storage of carbon can occur anywhere.
FW	Food	Spatial configuration	No relationship - it does not matter where fish production occurs assuming infrastructure is in place to transport to beneficiaries.
FW	Fibre	Spatial configuration	No relationship - it does not matter where reeds are grown, assuming infrastructure is in place to transport reeds to beneficiaries. Currently a large proportion of UK's reed product needs are met by imports.
FW	Energy	Quantity	No relationship - it is not considered realistic to increase the extent of the A/U unit e.g. rivers to provide more hydro power due to complexity of conditions that determine where these habitats occur.

Habitat type	Benefit	Characteristic	Relationship Justification
		Quality	No relationship - to get energy from freshwater A/U need to put the material capital in the right place to harness energy.
FW	Clean air	Quantity	No relationship - cannot influence the A/U for clear air benefit.
		Quality	
		Spatial configuration	
U	Food	Spatial configuration	No relationship- tend to be community farms/domestic gardeners and allotment holders.<br>Limited extent of cultivation and high proportion of impermeable areas restricting ability to change spatial configuration.
U	Fibre	Quantity	No relationship - cannot influence the A/U for fibre benefit.
		Quality	
		Spatial configuration	
U	Energy	Quantity	No relationship - cannot influence the A/U for energy benefit.
		Quality	
		Spatial configuration	
CM	Food	Spatial configuration	No relationship - the spatial configuration of the A/U cannot be influenced by society-restricted to interface between land and sea.
CM	Fibre	Spatial configuration	No relationship - the spatial configuration of the A/U cannot be influenced by society-restricted to interface between land and sea.
CM	Energy	Quantity	No relationship - the A/U cannot be influenced by society to provide an energy benefit. i.e. we harness what is already in place (tidal).
		Quality	
		Spatial configuration	

Habitat type	Benefit	Characteristic	Relationship Justification
CM	Clean water	Quantity	No relationship - Although sand dunes and shingle with a reasonable depth form shallow aquifers of clean water (used for small-scale local abstractions such as golf) (UK NEA), the quantity of the A/U cannot be influenced by society to increase the value of the benefit produced. i.e. we utilise what is there.
		Spatial configuration	No relationship - the spatial configuration of the A/U cannot be changed or influenced by human management to increase the value of the benefit produced.
CM	Clean air	Quantity	No relationship - the A/U cannot be changed or influenced by human management to provide a clean air benefit.
		Quality	
		Spatial configuration	
CM	Recreation	Spatial configuration	No relationship - the spatial configuration of the A/U cannot be changed or influenced by human management to increase the value of the benefit produced - restricted to interface between land and sea.
CM	Hazard protection	Spatial configuration	No relationship - the spatial configuration of the A/U cannot be changed or influenced by human management to increase the value of the benefit produced.
CM	Wildlife	Spatial configuration	No relationship - the spatial configuration of the A/U cannot be changed or influenced by human management to increase the value of the benefit produced
CM	Equable climate	Spatial configuration	No relationship - the spatial configuration of the A/U cannot be changed or influenced by human management to increase the value of the benefit produced.
M	Food	Quantity	No relationship - the quantity of the A/U cannot be changed or influenced by human management to increase the value of the benefit produced.
		Spatial configuration	No relationship - the spatial configuration of the A/U cannot be changed or influenced by human management to increase the value of the benefit produced.
M	Fibre	Quantity	No relationship - none of the characteristics of the A/U can be changed or influenced by human management to increase the value of the benefit produced.
		Quality	
		Spatial configuration	

Habitat type	Benefit	Characteristic	Relationship Justification
M	Energy	Quantity	No relationship - none of the characteristics of the A/U can be changed or influenced by human management to increase the value of the benefit produced.
		Quality	
		Spatial configuration	
M	Clean water	Quantity	No relationship - none of the characteristics of the A/U can be changed or influenced by human management to increase the value of the benefit produced.
		Quality	
		Spatial configuration	
M	Clean air	Quantity	No relationship - none of the characteristics of the A/U can be changed or influenced by human management to increase the value of the benefit produced.
		Quality	
		Spatial configuration	
M	Recreation	Quantity	No relationship - the quantity of the A/U cannot be changed or influenced by human management to increase the value of the benefit produced.
		Spatial configuration	No relationship - the spatial configuration of the A/U cannot be changed or influenced by human management to increase the value of the benefit produced.
M	Aesthetic	Quantity	No relationship - the quantity of the A/U cannot be changed or influenced by human management to increase the value of the benefit produced.
		Spatial configuration	No relationship - the spatial configuration of the A/U cannot be changed or influenced by human management to increase the value of the benefit produced.
M	Hazard protection	Quantity	No relationship - none of the characteristics of the A/U can be changed or influenced by human management to increase the value of the benefit produced.

Habitat type	Benefit	Characteristic	Relationship Justification
		Quality	
		Spatial configuration	
M	Wildlife	Quantity	No relationship - the quantity of the A/U cannot be changed or influenced by human management to increase the value of the benefit produced.
		Spatial configuration	No relationship - the spatial configuration of the A/U cannot be changed or influenced by human management to increase the value of the benefit produced.
M	Equable climate	Quantity	No relationship - the quantity of the A/U cannot be changed or influenced by human management to increase the value of the benefit produced.
		Spatial configuration	No relationship - the spatial configuration of the A/U cannot be changed or influenced by human management to increase the value of the benefit produced.

2. Negligible relationship

A/U	Benefit	Characteristic	Relationship Justification	Graph
MMH	Food	Quantity	MMH is of low/medium low importance in providing food. MMH naturally have low agricultural productivity due to soil properties, water logging and topography (sheep predominant use) therefore classed as poor quality agricultural land. Although we could influence the quantity and quality characteristics, this would be limited to the margins (e.g. change in lowland heath) and there would be negligible change in total benefit produced from the A/U over the next 25 yrs.	-
		Quality		-
MMH	Fibre	Quantity	MMH is of low/medium low importance in providing fibre, sheep wool by product of sheep meat-little market value. Although we could influence the quantity and quality characteristics, there would be negligible change in total benefit produced from the A/U over the next 25 yrs.	-
		Quality		-
MMH	Clean water	Quantity	The relationship is considered to be +L (none/negligible).<br><br>The quantity of the A/U could decrease through change in land use (e.g. to woodland, to enclosed farmland) or development, however the potential to increase the extent of the habitats is limited to the margins e.g. heath areas, majority of other subcomponents require specific topographic conditions to exist. As any changes in extent will be minimal, the impact to benefit produced over the next 25yrs will be none/negligible. Although MMH significant source of water (70% UK drinking water) –coincidental in location - quantity of the A/U does not significantly affect amount of water.	
MMH	Recreation	Quantity	The relationship is considered to be +L (none/negligible).<br><br>The quantity of the A/U could decrease through change in land use (e.g. to woodland, to enclosed farmland) or development, however the potential to increase the extent of the habitats is limited to the margins e.g. heath areas, majority of other subcomponents require specific conditions to exist. As any changes in extent will be minimal, the impact to benefit produced over the next 25yrs will be none/negligible.	-
		Quality	The relationship is considered to be +NL (none/negligible).<br><br>The use of MMH for recreation e.g. mountain biking, walking etc will largely be determined by access, and material capital investments e.g. trails, footpaths etc, and the maintenance of these. The impact to the benefit produced over 25yrs, resulting from a change in quality, is considered to be none/negligible.<br><br>Recreation = f [land (topography, altitude); material capital (management practices - trails, footpaths, access to rock faces for climbing)]	-

MMH	Aesthetics	Quantity	The relationship is considered to be +L (none/negligible).<br><br>It is considered that most of the aesthetic value of MMH is the scenery and sense of wilderness, and what is available will be highly valued.<br><br>The quantity of the A/U could decrease through change in land use (e.g. to woodland, to enclosed farmland) or development, however the potential to increase the extent of the habitats is limited to the margins e.g. heath areas, majority of other subcomponents require specific conditions to exist and therefore unlikely to be adversely affected by land use changes. As any changes in extent will be minimal, the impact to benefit produced over 25yrs will be none/negligible.	-
EF	Food	Spatial configuration	The relationship is considered to be +NL (none/negligible).<br><br>Although we can influence the spatial configuration of enclosed farmland, this does not influence the total benefit produced or its value i.e. it does not matter where produce food. It is also acknowledged that some geology/soils are more fertile than others, however again, society cannot influence where these occur, can just utilise for best output.	-
EF	Fibre	Quantity	Enclosed farmland is of low/medium low importance in providing fibre (considered to be a secondary crop to food production). Although we could influence the quantity, quality and spatial configuration characteristics, there would be negligible change in total value of benefit produced because of low ES provision from the A/U over the next 25 yrs.	-
		Quality		-
		Spatial configuration		-
EF	Energy	Spatial configuration	The relationship is considered to be +NL (none/negligible).<br><br>Although we can influence the spatial configuration of EF, this does not influence the value of the benefit produced i.e. it does not matter where produce biofuel. It is also acknowledged that some geology/soils are more fertile than others, however again, society cannot influence where these occur, can just utilise for best output.	-
EF	Clean water	Spatial configuration	The relationship is considered to be +NL (none/negligible).<br><br>Although we can influence the spatial configuration of EF, this is not considered to significantly change the amount of the benefit produced - other over-riding factors would affect location of farms.	-
EF	Clean air	Quantity	The relationship is considered to be -NL (none/negligible).<br><br>Although we can influence the quantity of EF, and therefore potential for air quality issues (vehicle emissions), this is not considered to significantly change the amount of the benefit produced. (urban A/U greater contributor).	-

EF	Recreation	Quantity	The relationship is considered to be +NL (none/negligible).<br><br>Although we can change the quantity of EF, this is not considered to significantly change the benefit produced - this is primarily determined by the quality characteristic .i.e. access.	-
EF	Aesthetics	Quality	The relationship is considered to be +NL (none/negligible).<br><br>Although we can influence the quality of EF to improve aesthetics e.g. greater heterogeneity of farming types in the landscape is likely to be more aesthetically pleasing than a homogenous farming landscape, this is not considered to significantly change the amount of the benefit produced.	-
		Spatial configuration	The relationship is considered to be +NL (none/negligible).<br><br>Although we can influence the spatial configuration of EF to bring closer to people, this is not considered to significantly change the amount of the benefit produced and overriding factor of locality.	-
EF	Hazard protection	Quantity	The relationship is considered to be -NL (none/negligible).<br><br>Although we can influence the quantity of EF, this is not considered to significantly change the amount of the benefit produced – quality more important. However it is assumed that there is a level of soil erosion under baseline quality conditions, and therefore an increase in area of EF would have some impact flooding (increased sediment in rivers potentially causing flooding problem)	-
EF	Equable climate	Quantity	The relationship is considered to be -NL (none/negligible).<br><br>Although the area of enclosed farmland can be increased, the stocking density of livestock that give rise to methane emissions is part of the quality characteristic at baseline it is assumed some increase.	-
SNG	Food	Quantity	SNG is of low/medium low importance in providing food. Although we could influence the quantity, quality and spatial configuration characteristics for food, there would be negligible change in benefit produced from the A/U over the next 25 yrs. Livestock grazing on improved grasslands has been included in the Enclosed Farmland A/U.	-
		Quality		-
		Spatial configuration		-
SNG	Fibre	Quantity	Semi-natural grasslands is of low/medium low importance in providing fibre. Although we could influence the quantity, quality and spatial configuration characteristics, there would be negligible change in benefit produced from the A/U over the next 25 yrs.	-
		Quality		-
		Spatial configuration		-

SNG	Recreation	Quantity	The relationship is considered to be +NL (none/negligible).<br><br>It is assumed that all SNG could be replaced with another A/U. The first units of grassland will be highly valued, however, at a critical amount i.e. enough grassland to satisfy recreational demands, any increases over this will not be as valued. The overall increase in area of semi-natural grassland is considered to be limited, as it is dependent on underlying geological conditions.<br><br>As only 1%, potential change in quantity is limited and also ability to extend (due to underling geological conditions required).	-
		Quality	The relationship is considered to be +NL (none/negligible).<br><br>The recreational benefits from SNG will be governed by low level management e.g. light grazing, management of footpaths. Overall it is considered that there is limited requirement to improve the quality of the A/U for recreational benefits assuming access in place.<br><br>Recreation = f [**species**; **soils**; **land** (topography); **material capital** (light grazing to minimise scrub, footpaths, bridlepaths)]	-
SNG	Aesthetic	Quantity	The relationship is considered to be +NL (none/negligible).<br><br>It is assumed that all SNG could be replaced with another A/U. The first units of grassland will be highly valued, however, at a critical amount i.e. enough grassland to satisfy recreational demands, any increases over this will not be as valued. The overall increase in area of semi-natural grassland is considered to be limited, as it is dependent on underlying geological conditions.<br><br>As only 1%, potential change in quantity is limited and also ability to extend (due to underling geological conditions required).	-
SNG	Equable climate	Quantity	The relationship is considered to be +NL (none/negligible).<br><br>It is assumed that all semi-natural grassland could be replaced with another A/U therefore the value crosses the origin. The first units of grassland will be highly valued, however, at a critical amount i.e. enough grassland to satisfy recreational demands, any increases over this will not be as valued. The overall increase in area of semi-natural grassland is considered to be limited, as it is dependent on underlying geological conditions.	-
W	Food	Quantity	Woodland A/U is of low importance in providing food. Although we could influence the quantity, quality and spatial configuration characteristics, there would be negligible change in total benefit produced from the A/U over	-

		Quality	the next 25 yrs.	-
		Spatial configuration		-
W	Energy	Quality	The relationship is considered to be +NL (none/negligible).<br><br>It is the quantity that drives the change in benefit more than the quality i.e. amount of timber grown for biofuel.<br><br>However, aspects of quality of the A/U can affect the yield. In a poor quality environment e.g. high levels of acidification, low nutrient cycling, tree growth will be poor and therefore limit yield.<br><br>It may also be possible to select the species that are grown, to select those with quickest growth rates. Improvements in material capital to harvest the timber will also increase the amount of output.<br><br>Biofuel yield = f [**species** (quick growing), **ecological communities** (invasives, pests and disease); **soils** (decomposers, nitrifying bacteria - nitrogen fixation, nutrient cycling); **freshwaters** (groundwater); land (altitude, gradient); **atmosphere** (rain, temperature, nitrogen, carbon dioxide, wind); minerals (potassium, magnesium); **material capital** (management - coppicing, felling, crop rotation, irrigation, processing, machinery, transport, pest control, nutrient enrichment, pollution - SO2)]<br><br>Overall, the value of woodland for biofuel will be lower than the value for timber, as biofuel is readily substitutable and therefore there is a lower demand.	-
W	Clean air	Quality	The relationship is considered to be +NL (none/negligible)<br><br>It is the quantity that drive the change in benefit i.e. more woodland equals greater potential to absorb pollutants and increase O_2 production.	
		Spatial configuration	The relationship is considered to be +NL (none/negligible).<br><br>UK NEA suggests that trees could be planted around livestock units to reduce pollution spreading, and it is considered that the same approach could be applied to roads to absorb pollutants. However, the overall change in benefit is considered to be minimal, with the greatest change driven by quantity of trees (0_2 production, pollutant absorption). Please note, the importance of greenspace and trees is considered in the urban A/U.	-
FW	Food	Quantity	The relationship is considered to be +L (none/negligible).<br><br>The overall value of food from freshwater (fish) is considered to be low when compared to EF (crops and	-

			livestock).<br><br>As the number of waterbodies increases, the potential for use for aquaculture also increases. However, the overall change in extent is considered to be small i.e. cannot significantly increase number of rivers or lakes.	
FW	Fibre	Quantity<br><br>(Wetland)	The relationship is considered to be +L (none/negligible).<br><br>Although the UK NEA identifies that there is a strong demand for quality thatching reed, this is considered to be relatively low in value compared to other fibre products e.g. timber from woodland.<br><br>It is also considered that the area of reedbed available for harvesting is unlikely to increase considerably if the area of 'freshwater' increases due to prevailing conditions limiting how much habitat can be created.	-
		Quality<br><br>(Wetland)	The relationship is considered to be +NL (none/negligible).<br><br>The quality of the wetland subcomponent will affect reed growth and therefore amount that can be harvested and sold. However the future provision is considered to be low.<br><br>In a degraded wetland, it is considered unlikely that there would be sufficient quantity of reeds to be commercially viable to harvest. However, as the quality of the wetland increases, the quantity and quality of reeds available for harvesting will increase significantly. At a critical point, further improvements will not significantly increase value from reed yield.<br><br>Reed yield = f [**species** (common reed), **freshwater** (water, floodplain, low flows, submergence - 300mm water depth in spring, soils-clays and silts, nutrient enrichment), **land** (gradient), atmosphere (temperature), **material capital** (cutting and harvesting)]	-
FW	Clean water	Spatial configuration	The relationship is considered to be +NL (none/negligible).<br><br>Storage reservoirs and water treatment works are typically located within close proximity to populations, or where rainfall or river flows are high. However, there is an historic context to this as water supply components will be developed where cities have developed i.e. close to populations. Distribution networks connect a range of water supply components which may be some miles from the beneficiary.<br><br>The location of wetlands can alter the effectiveness of purification, with some being located in areas of between sources of pollutants and the main watercourse. However, this is considered to have a negligible effect to the overall cost of treating water for use.	-

FW	Recreation	Quantity	The relationship is considered to be +L (none/negligible).<br><br>It is assumed that freshwater is valued moderately high for aesthetics e.g. coastal margins and MMH more valued.<br><br>Although you can change the area of some of the subcomponents e.g. wetlands, reservoirs, you cannot significantly change the area of the A/U for a recreational benefit. Wetlands and reservoirs are normally created for another purpose (e.g. wildlife, clean water) and recreation is a by-product.	-
FW	Aesthetics	Quantity	The relationship is considered to be +L (none/negligible).<br><br>It is assumed that freshwater is valued moderately high for aesthetics e.g. coastal margins and MMH more valued.<br><br>It is considered that you cannot significantly change the area of the A/U, just around the margins such as the wetland subcomponent. These changes in area will not give rise to a significant change in value.	-
		Spatial configuration	The relationship is considered to be +NL (none/negligible).<br><br>The proximity of freshwater to populations will be valued, although there will be a certain distance which is acceptable, and any improvements on this will not be valued as greatly and ability to change limited.	-
FW	Hazard protection	Quantity	The relationship is considered to be -NL (none/negligible).<br><br>As the number of waterbodies increases, the potential for flooding also increases. With a low extent of waterbodies, the avoided cost of flood protection is high, as waterbodies increases, and therefore risk of flooding increases, this avoided cost decreases. However, it is considered that you cannot significantly change the area of the A/U, just around the margins such as the wetland subcomponent. These changes in area will not give rise to a significant change in benefit.	-
U	Food	Quantity	The urban A/U is of low/medium low importance in providing food. Although we could influence the quantity and quality characteristics, there would be negligible change in total value of benefit produced from the A/U over the next 25 yrs.	-
		Quality		-
U	Clean water	Spatial configuration	The relationship is considered to be +NL (none/negligible).<br><br>Urban greenspaces could be arranged to act as interceptors to pollution before it enters watercourses. However, the change in value to the clean water benefit is considered to be negligible with the greatest impact arising through replacement techniques e.g. SUDS, material capital investments such as Thames Tideway Tunnel (these not considered as part of the current scope of work to inform the risk register)	-

U	Clean air	Spatial configuration	The relationship is considered to be +NL (none/negligible).<br><br>It is considered that urban greenspaces could be configured to maximise potential to scavenge pollutants however the change of value to the clean air benefit is considered to be negligible given the overall impact of the built urban environment on air quality.	-
U	Hazard protection	Quality	The relationship is considered to be +NL (none/negligible)<br><br>The quality of the built urban environment could be improved by reducing the area of impermeable surfaces, and maximising potential of greenspaces to reduce surface water runoff.	-
		Spatial configuration	The relationship is considered to be +L (none/negligible).<br><br>It is considered that urban greenspaces could be configured to maximise potential to intercept rainfall and reduce surface water runoff however the change of value to the protection from hazards benefit is considered to be negligible given the overall impact of the built urban environment and reliance on replacement techniques to reduce the impact.	-
CM	Food	Quantity<br><br>Saltmarsh and sand dunes	The relationship is considered to be +NL (none/negligible)<br><br>Older established saltmarsh and sand dune grasslands are used for grazing livestock (predominantly sheep) (UK NEA 2011). The current provision of food from this A/U is considered to be low when compared to EF and M. Although there is potential to reduce the quantity of these subcomponents through land use change, the ability to increase them is limited as they are the ultimate stage of succession for these habitats.	
		Quality<br><br>Saltmarsh and sand dunes	The relationship is considered to be +NL (none/negligible)<br><br>The saltmarsh and sand dunes which support livestock typically have a soil profile to support grass. Anything that affects the store of soil will affect the amount of benefit that can be produced. However, this is considered to be minimal as the grassland successional stage is well established and least vulnerable to erosion.	
CM	Fibre	Quantity	The relationships are considered to be +NL (none/negligible)<br><br>Coastal margins are of low importance in providing fibre (wool). Although we could influence the quantity and quality characteristics (spatial configuration cannot be changed), there would be negligible change in total benefit produced from the A/U over the next 25 yrs.	-
		Quality		-
CM	Clean water	Quality	The relationship is considered to be +NL (none/negligible).<br><br>It is assumed that as the quality of the habitat increases, its ability to purify also increases. It is considered that a limited number of aquifers benefit from this process, with the majority of aquifers in England being inland, and	-

			therefore the overall value of an increase in quality is minimal. As stated in the UK NEA, Dungeness is the only shingle site which provides a local source of drinking water. The benefit in purification is to the marine A/U.<br><br>Clean water = f [**coasts** (substrate); **freshwater** (aquifer); **material capital** (abstraction wellfield)]	
CM	Recreation	Quantity	The relationship is considered to be +L (none/negligible).<br><br>The ability to increase the quantity is limited e.g. some subcomponents only such as saltmarsh. Minor changes in the quantity of these habitats are not considered to be significantly valued for recreation.	-
CM	Aesthetic	Quantity	The relationship is considered to be +L (none/negligible).<br><br>The ability to increase the quantity is limited e.g. some subcomponents only such as saltmarsh. Changes in the quantity of these habitats are not considered to be significantly valued for aesthetics.	-
M	Aesthetic	Quality	The relationship is considered to be +NL (none/negligible).<br><br>The quality of the marine environment, and wider sea views, can be affected by offshore windfarms (perceived as both positive and negative impacts on landscape). The change in value of the benefit is considered to be none/negligible.	-
M	Equable climate	Quality	The relationship is considered to be +NL (none/negligible).<br><br>Marine organisms regulate the climate by acting as a sink for carbon dioxide and facilitating burial of carbon in seabed sediment. This is done by photosynthesis and also storage of carbon in shells (calcium carbonate).<br><br>The abundance and diversity of marine flora and fauna will be primarily determined by the quality of the water. This will increase up to a critical point, after which any increase in quality, and therefore associated species abundance, will be less valued. However, our ability to change or influence the benefit by human management is considered to be limited.<br><br>Equable climate = f [**species** (crustaceans, molluscs); **ecological communities** (phytoplankton, $CaCO_3$ absorption); **ocean** (salinity, temperature, pH); **pressures** (pollution)]	-

3. Minor relationships

A/U	Benefit	Characteristic	Relationship Justification	Graph
MMH	Energy	Quantity<br><br>Blanket bog and heath (habitats on peatland)	The relationship is considered to be +L (minor/moderate).<br><br>Peat is a non-renewable energy source; only the rate of extraction/consumption can be managed.<br><br>The quantity of the A/U could decrease through change in land use (e.g. heaths and bogs to grassland) or development, however the potential to increase the extent of the habitats is limited e.g. blanket bog requires certain conditions to exist. Therefore any changes in extent will be small.<br><br>The timescales over which appropriate conditions need to be present to allow the formation of peat are considerable (decades). Peat has a slow rate of natural regeneration (mms per year), and therefore management would aim to maintain the 'stock' of peat that could be extracted for fuel, as this could be degraded. However, the change in quantity is often driven by a change in quality e.g. soil erosion (see below).	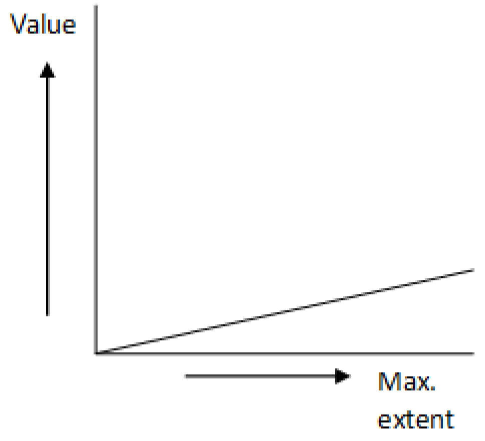
		Quality	The relationship is considered to be +NL (minor/moderate).<br><br>Peat will only form under certain conditions. If you graze or burn blanket bog, the species required for peat formation e.g. sphagnum moss etc, will be lost by the resultant lowering of the water table and drying of the existing peat layer. The vegetation will be replaced by heath species typical of drier conditions.<br><br>Suitable land management is therefore crucial in determining whether the stock of peat will be retained by ensuring continued conditions for it formation.<br><br>The current provision of peat for energy is low and localised	

			(UK NEA 2011, pg 126), and the UK Government is implementing measures to reduce peat use.<br><br>Peat formation = f [**species** (sphagnum moss etc); **ecological communities** (photosynthesis and carbon locking); **soils** (high acidity, organic matter and water holding capacity, nutrient availability); **atmosphere** (temperatures, rainfall, CO2, N); freshwater (high water table); **land** (low gradient); **material capital** (extraction methods, land management - burning and grazing regimes)]	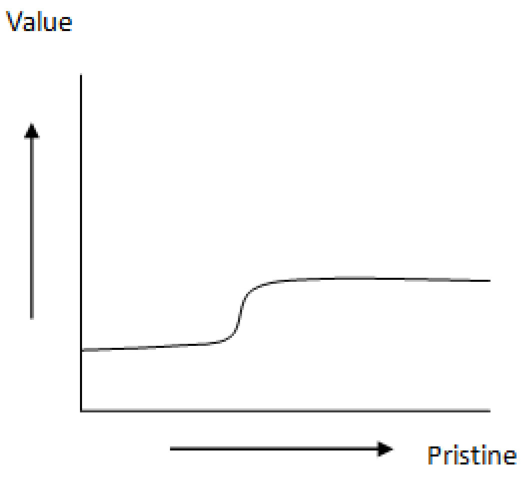
MMH	Wildlife	Spatial configuration	The relationship is considered to be +NL (minor/moderate).<br><br>The connectivity of habitats, e.g. blanket bogs and heaths, is important for maintaining wildlife.<br>However, the ability to fragment with other land uses and/or provide better connectivity is considered to be limited by underlying conditions required for formation (e.g. geology, altitude, high precipitation etc.)	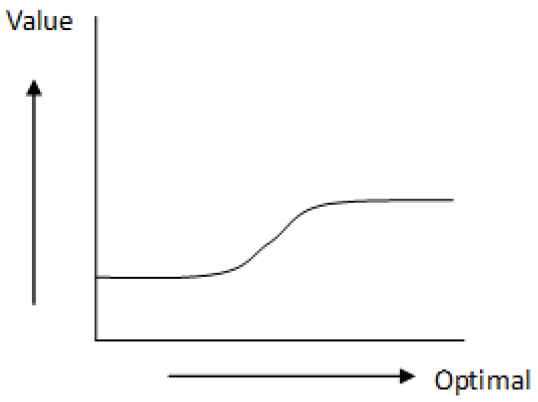

MMH	Equable climate	Quantity<br><br>Blanket bog	The relationship is considered to be +L (minor/moderate).<br><br>The quantity of the A/U could decrease through change in land use (e.g. heaths and bogs to grassland) or development, however the potential to increase the extent of the habitats is limited e.g. blanket bog requires certain conditions to exist. Therefore any changes in extent will be small. However, given the importance of blanket bog in carbon sequestration, the impact in benefit produced over 25yrs is considered to be minor/moderate.	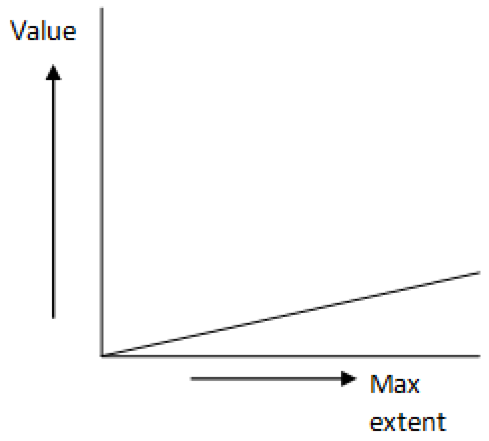
EF	Energy	Quantity	The relationship is considered to be +NL (minor/moderate).<br><br>There is potential to both increase and decrease the area of the A/U, and the proportion of the A/U which is devoted to production of biofuels rather than food. As the quantity of the A/U increases so does the potential to produce biofuel and therefore resultant value. However, this will be limited by the market demand and therefore there will be a critical area required, after which increases in area will no longer be as highly valued.<br><br>Given the current provision of benefit from A/U, considered to be minor/moderate.	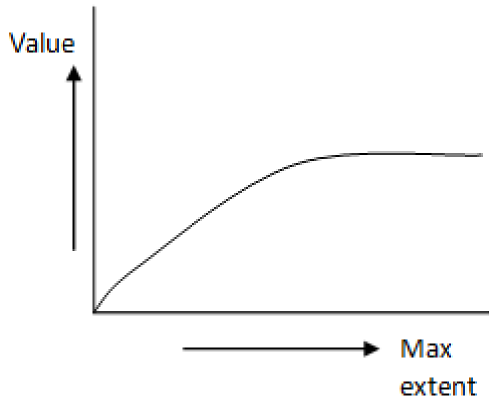

		Quality	The relationship is considered to be +NL (minor/moderate).<br><br>The amount of biofuel that can be produced from EF will depend upon the quality of the habitat, but more importantly the material capital investment made to produce the crop. Poor quality habitats will not produce large amounts of biofuel and therefore the value will be low. As this increases, the value will significantly increase up to an optimal point, where after further improvements in quality will no longer give such substantial increases in benefit value.<br><br>Biofuel crop yield = f [**species** (crop type); **soils** (agricultural Grade I – V); **land** (aspect, altitude, gradient, exposure to wind); **atmosphere** (temperature and rainfall); **freshwater** (groundwater); **minerals** (potassium, magnesium); **ecological communities** (pollination, invasive species/disease); **material capital** (management practices e.g. irrigation, pest/disease control, nutrient enrichment, aeration of soil, crop rotation)]<br><br>Given the current provision of benefit from A/U, considered to be minor/moderate.	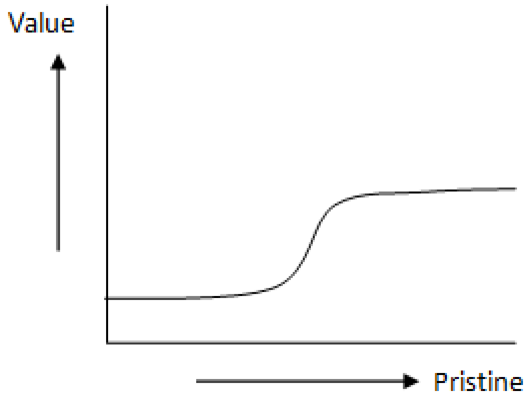
EF	Recreation	Quality	The relationship is considered to be +NL (minor/moderate).<br><br>The quality of enclosed farmland can significantly affect the value of recreation e.g. through use of set aside to create game shooting areas, improve access arrangements.<br><br>Recreation = f [**species**; **ecological communities** (pollination); **freshwater** (waterbodies present in enclosed farmland A/U); **land** (aspect, altitude, gradient); **material capital** (management practices e.g. buffer strips, set-aside, management of activities, signs/waymarks, well maintained footpaths/bridleways)]	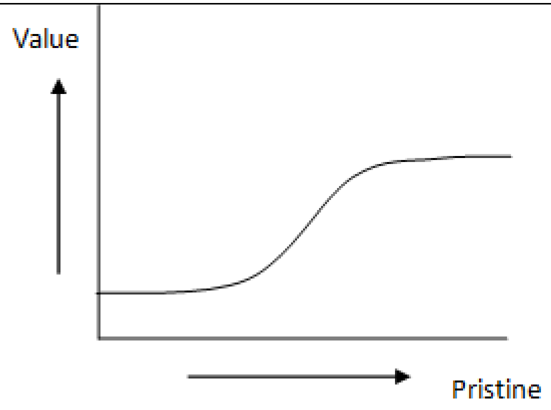

EF	Aesthetic	Quantity	The relationship is considered to be +NL (minor/moderate).<br><br>There is potential to both increase and decrease the area of the A/U, which could result in a loss of the farming heritage from the wider landscape. The future provision to aesthetics is considered to be minor/moderate.	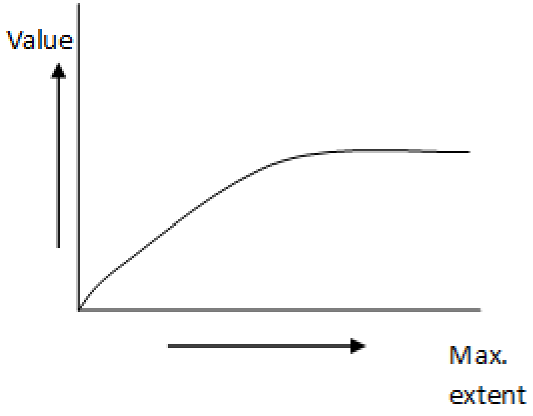
SNG	Wildlife	Spatial configuration	The relationship is considered to be +NL (minor/moderate)<br><br>Semi-natural grassland is geographically constrained by underlying geology, but land use changes could interrupt the overall connectivity. However as SNG occupies only 1% of England land, and a large proportion is protected to some degree (68% of SNG is within SSSI, other designations also protect e.g. SAC, AONB), the ability to influence connectivity is considered to be limited, although could be significant in terms wildlife abundance and diversity.	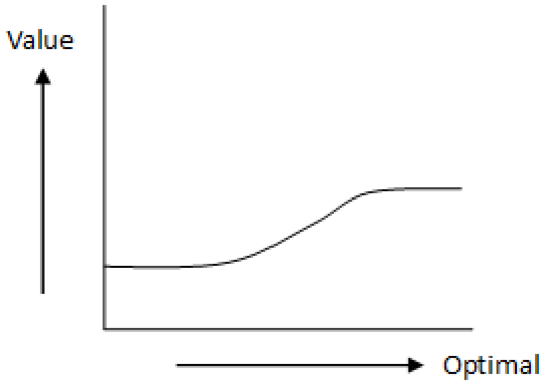

W	Energy	Quantity	The relationship is considered to be +NL (minor/moderate).<br><br>The quantity of the A/U can be changed, with the potential for all areas of woodland to be converted to a different land use. Therefore the first unit of woodland will be highly valued. However, there will be a point where the area of woodland satisfies market demand, and any increase in quantity thereafter will not be as valued.<br><br>Overall, the value of woodland for biofuel will be lower than the value for timber, as biofuel is readily substitutable and therefore there is a lower demand.	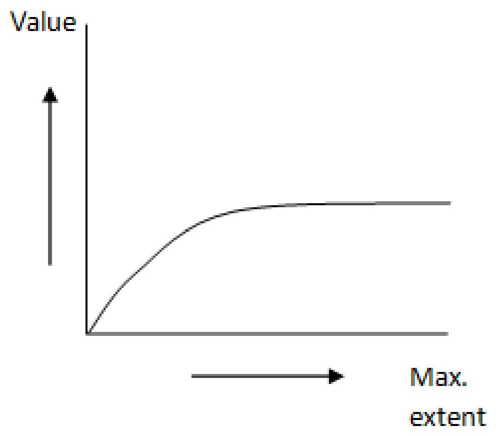
W	Recreation	Quality	The relationship is +NL (minor/moderate).<br><br>The quality of recreation will largely be governed by the management practices i.e. improving access, felling, coppicing, and creating recreational opportunities e.g. mountain biking trails, zip lines etc. The species composition of the woodland is not likely to be as important for recreational activities.<br><br>As management improves the recreational facilities, the value will increase. At a certain point, further improvements will no longer be as valued.<br><br>Recreation value = f [**species**, **ecological communities (**invasives, pests and disease); **soils** (decomposers, nitrifying bacteria - nitrogen fixation, nutrient cycling); **freshwaters** (groundwater); **land** (altitude, gradient); **atmosphere** (rain, temperature, nitrogen, carbon dioxide, wind); **minerals** (potassium, magnesium); **material capital** (management - paths, bridleways, coppicing, felling, recreational equipment,	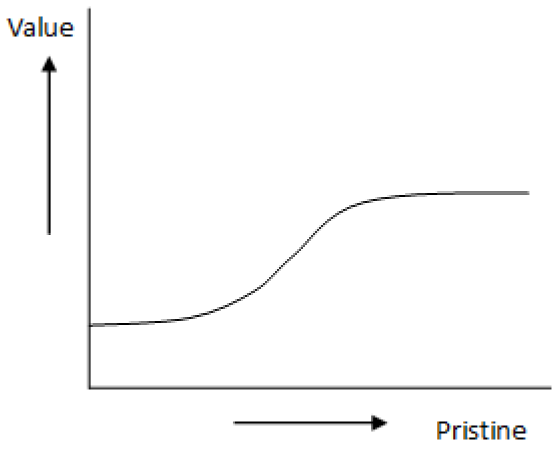

			pollution - SO_2)]	
W	Equable climate	Quality	The relationship is considered to be +NL (minor/moderate).<br><br>Although different species may have different capacities and uptake rates of CO_2 it is predominantly the quantity of woodland that will give the greatest benefit. However the age of the woodland will also be important in CO_2 uptake in younger woodlands greater than mature woodlands. Species composition may also affect rates of uptake.<br><br>Timber yield = f [**species** (fast growing e.g. eucalyptus), **ecological communities** (invasives, pests and disease); **soils** (decomposers, nitrifying bacteria - nitrogen fixation, nutrient cycling); freshwaters (groundwater); **land** (altitude, gradient); **atmosphere** (rain, temperature, nitrogen, carbon dioxide, wind); **minerals** (potassium, magnesium); **material capital** (management - coppicing, felling, crop rotation, pest control, pollution - SO_2)]	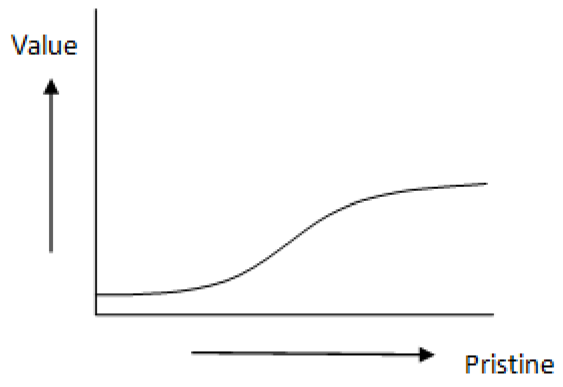

FW	Food	Quality	The relationship is considered to be +NL (minor/moderate).<br><br>Overall value of food from freshwater (fish), and the contribution quality has to this, will be low.<br>It is considered that the majority of fish production is undertaken in artificially created habitats, with high levels of human management, although noted that high value fish such as salmon can only be reared in high quality water courses.<br><br>In a degraded/poor environment (either for water abstraction for artificial habitats or the natural system), the quantity and quality of water will limit fish production, with improvement in quality, fish production will increase. At a critical point, further improvements will not significantly increase value from fish production.<br><br>Fish yield = f [**freshwater** (rivers, standing open water, water - volume, quality (levels of O2, temperatures, nutrient levels), **land** (gradient, altitude), **ecological communities** (phytoplankton, zooplankton, aquatic vegetation), **species** (biomass, disease/pests), **manufactured capital** (controlled growing facilities (artificial waterbodies), fish passes on natural systems, water abstraction), **human management** (stocking densities, management of fish life cycle, disease/pest control, artificial feeding, nutrient enrichment)]	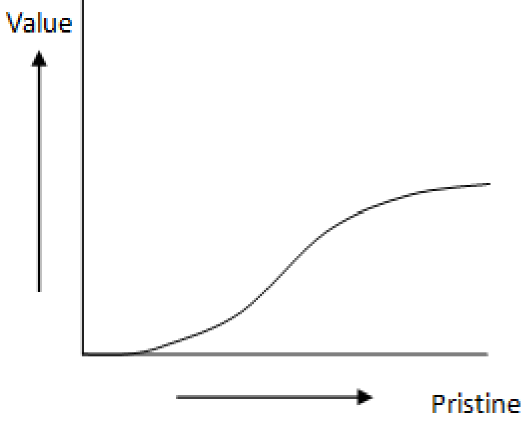
FW	Energy	Spatial configuration	The relationship is considered to be +L (minor/moderate).<br><br>The value of benefit realised is dependent on the positioning of material capital to harness the energy i.e. create dams in upland areas where there is sufficient flow, vertical distance (head) and volume of water, with low suspended sediment.	

				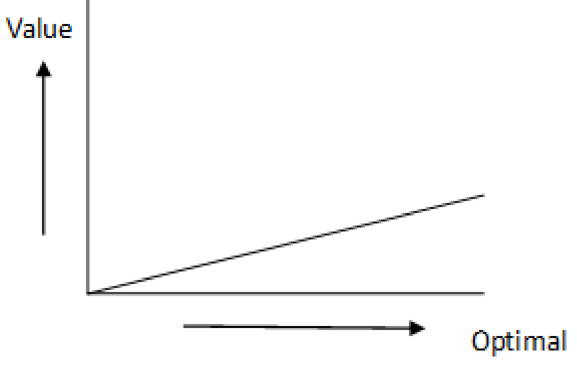
FW	Recreation	Spatial configuration	The relationship is considered to be +NL (minor/moderate).<br><br>The proximity of freshwater to populations will be valued, although there will be a certain distance which is acceptable, and any improvements on this will not be valued as greatly. It also considered that there is a limit on the number of waterbodies that could be created near to populations to increase value from spatial configuration.	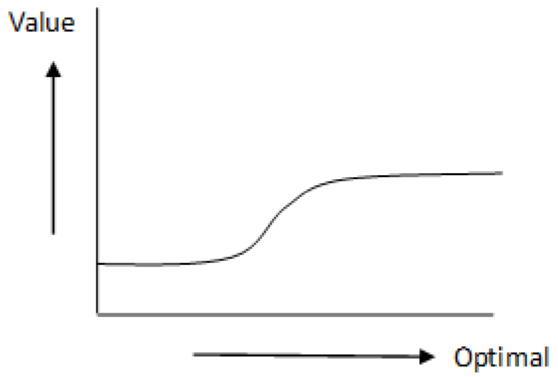

U	Clean water	Quality<br><br>Built urban	The relationship is considered to be +NL (minor/moderate).<br><br>The impact from the built urban subcomponent on clean water is generally derived from combined sewer overflows during storm events and polluted surface water runoff that gets into the watercourses. Changes to use of cars, littering, reduced loading etc could reduce this. The main improvements would require the use of SUDS, oil interceptors etc and material capital investments e.g. Thames Tideway Tunnel are required. However these are all replacement techniques to reduce the impact of the urban A/U and not considered as part of the scope for the risk register work.	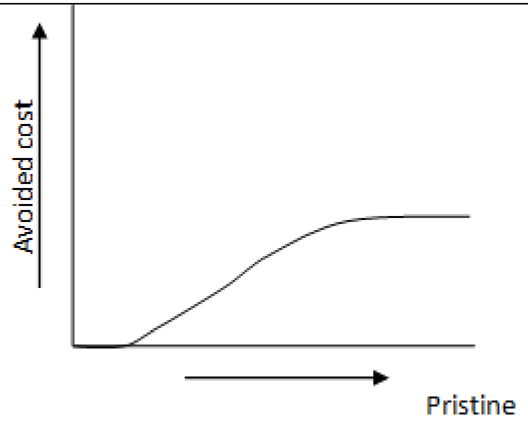
U	Recreation	Quality<br><br>Greenspace	The relationship is considered to be +NL (minor/moderate).<br><br>Poor quality greenspace is considered to be that which is unmanaged e.g. overgrown towpath, or possibly lacking in facilities e.g. greenspace with no play area in close proximity to residential housing i.e. active enjoyment of the greenspace is difficult. Pristine quality greenspace is that which is well managed and offers the facilities desired by the public.<br><br>The initial unit of greenspace within the urban will be valued, but as the quality improves, this will increase. However, after a certain level of improvements, any additions are no longer as highly valued i.e. the marginal increase is less.<br><br>= f [**ecological communities** (urban greenspace - parks, gardens, towpaths), **material capital** (management practices e.g. mowing, construction of playgrounds, football pitches, maintenance of these features)]	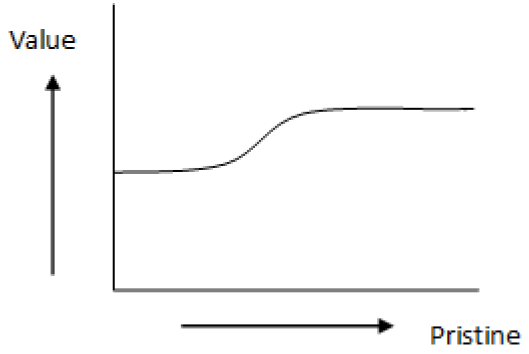

U	Equable climate	Quality<br><br>Built urban	The relationship is considered to be +NL (minor/moderate).<br><br>The built urban subcomponent of the A/U is considered to have a negative effect on flooding due to the extent of impermeable surfaces. A poor environment would be one of high impermeability, whereas a well designed environment would incorporate urban green spaces to intercept rainfall and reduce surface water runoff.<br><br>Surface water flooding = f [**ecological communities** (vegetation); **soils** (permeability); **atmosphere** (rainfall)]	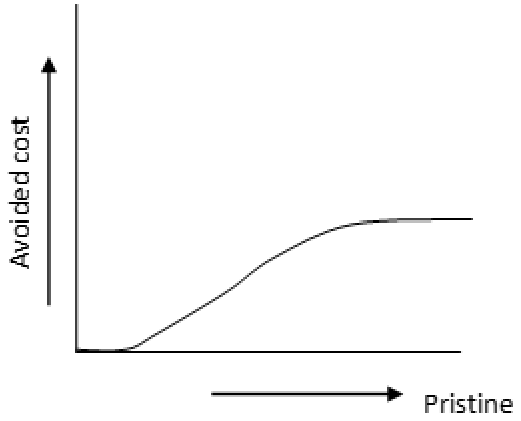

		Spatial configuration	The relationship is considered to be +NL (minor/moderate)<br><br>It is considered that urban greenspaces could be configured to maximise potential to intercept rainfall and reduce surface water runoff however the change of value to the protection from hazards benefit is considered to be negligible given the overall impact of the built urban environment and reliance on replacement techniques to reduce the impact.	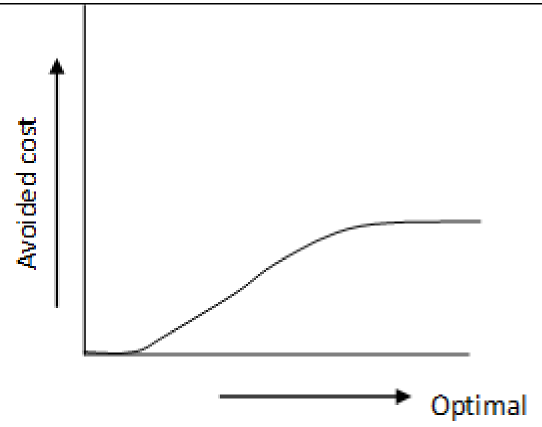
M	Recreation	Quality	The relationship is considered to be +NL (minor/moderate).<br><br>The value of recreation can be improved with artificial reefs (e.g. surfing) and decreased through no-catch zones (angling).	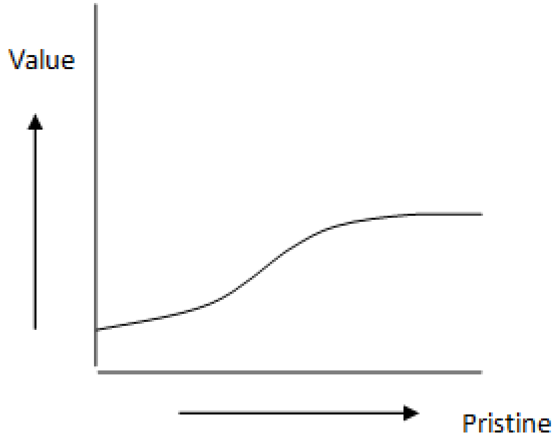

4. Prioritised Relationships

Major relationships

A/U	Benefit	Characteristic	Relationship Justification	Graph
MMH	Clean water	Quality<br><br>Blanket bog	The relationship is +NL (major).<br><br>As the quality of blanket bog improves, the cost of treating water to drinking water standards will decrease - peat accumulation immobilises nutrients i.e. when not degraded, organic carbon, N etc held in place which would otherwise be released into water. The impact of degraded peatland on clean water is considered to be significant.<br><br>Clean water = f [**ecological communities** (vegetation - nutrient cycling, pollutant absorption), **soils** (pH, nutrient concentrations (TOC, nitrate, phosphate, ammonium), erosion, infiltration), **freshwater** (high water table) **land** (altitude, gradient), **atmosphere** (temperature and rainfall); **pressures** (management practices e.g. low intensity grazing, low drainage gripping, limit burning)]	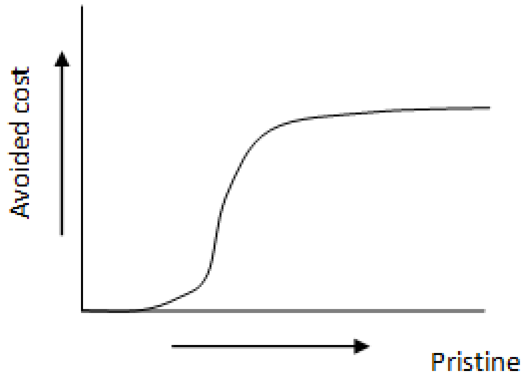
MMH	Aesthetics	Quality	The relationship is considered to be +NL (major).<br><br>The quality of MMH can affect the 'sense of experience' gained from the A/U. Although the value of MMH for aesthetics will largely be determined by the land form which cannot be influenced by society. Appropriate management of the habitats e.g. heath could be considered to enhance views, with degraded habitats not offering the same 'scenery' as good or pristine habitats. (Note, the aesthetic appeal of special plant and animal life is considered under the 'wildlife' benefit).	

A/U	Benefit	Characteristic	Relationship Justification	Graph
			Aesthetics = f [**land** (topography, altitude); **material capital** (management practices - burning, grazing, gripping)]	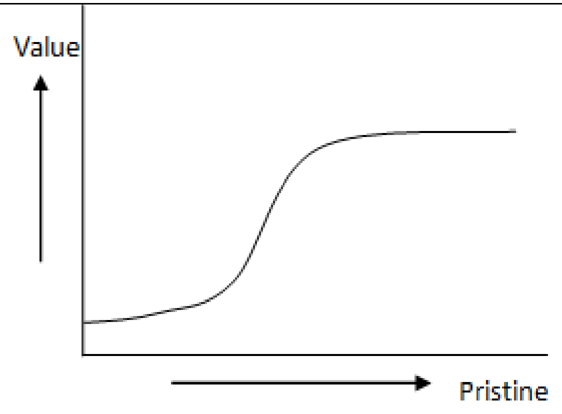
		Spatial configuration	The relationship is considered to be +NL (major).<br><br>Although the spatial configuration of the A/U cannot be influenced by society as it is defined by altitude (over 300m), the extent of it, and therefore aesthetic appeal, can be interrupted if the landscape is fragmented by urban area, tall structures or other land uses i.e. anything that interrupts the view, sense of wilderness. A continuous landscape is considered to be highly valued for aesthetics and therefore any impact on this are considered to be significant.	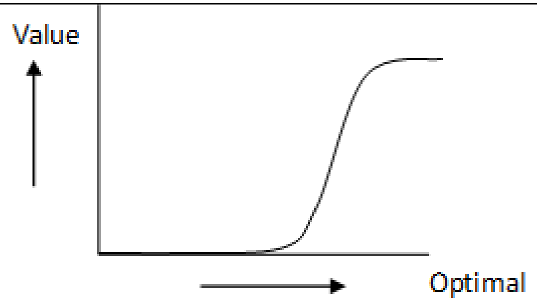

A/U	Benefit	Characteristic	Relationship Justification	Graph
MMH	Hazard protection	Quantity	The relationship is considered to be -NL (minor/moderate) for heaths and fire risk. The relationship between blanket bog and flooding is also considered to be -NL but there is a high level of uncertainty on significance.<br><br>There is the possibility of decreasing and to some extent increasing the quantity (although small) of some of the MMH subcomponents (blanket bogs and heaths) through land use changes e.g. afforestation, expansion of enclosed farmland.<br>Wildfire is a risk, with heath being particularly susceptible and therefore if the quantity increased, the potential risk of fire would also increase.<br><br>There is uncertainty around the role of blanket bog and flooding. As detailed in UK NEA 2011, historically have considered blanket bog as acting as a sponge and holding water, however good quality blanket bog would have a high water table, and therefore little capacity to hold additional water during a storm event. This could give rise to surface water runoff which would lead to increased flooding downstream.<br><br>There is also some uncertainty over the role of quantity in soil erosion and slope instability. Assuming the current baseline quality (eroding blanket bog) it would be assumed that an increase in quantity would have a negative effect on soil stability.<br><br>**Given the uncertainty of the relationships, this has been upgraded to major and will therefore be taken forward for further consideration in the risk register.**	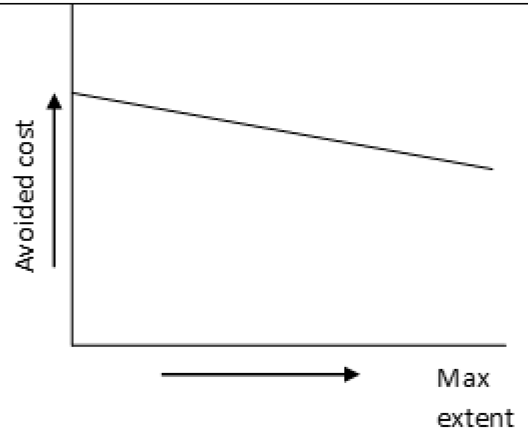

A/U	Benefit	Characteristic	Relationship Justification	Graph
		Quality	The relationship is considered to be +NL (minor/moderate) for both fire and soil stability.<br><br>Wildfire risk can be decreased by managed burns to reduce the biomass present (UK NEA), with older heath having a higher fuel load (certain). Degraded blanket bog is also likely to increase the risk of fire, as the drier habitat will potentially assist in the spread of fire whilst a better quality habitat, which is wetter, is likely to reduce the spread (uncertain).<br><br>It is unclear how blanket bog affects flooding (as stated above, the high water table could lead to runoff), however it is considered that a degraded blanket bog with grips and gullies would allow more efficient runoff of surface water during a storm event and therefore potentially increase flooding downstream (uncertain).<br><br>A degraded blanket bog will experience soil erosion which could lead to slope instability (uncertain).<br><br>Wildlife risk = f [**ecological communities** (heath biomass, blanket bog); **soils** (eroding); **freshwater** (low water table); **atmosphere** (temperature, rainfall); **pressures** (management practices e.g. burning regime)]<br><br>Flooding risk = f [**ecological communities**; **soils** (pH, nutrient concentrations (TOC, nitrate, phosphate, ammonium), erosion, infiltration), **freshwater** (water table) **land** (gradient), **atmosphere** (rainfall); **pressures** (management practices e.g. drainage gripping, burning)]<br><br>Soil erosion = f [**ecological communities**; **soils** (pH, nutrient concentrations (TOC, nitrate, phosphate,	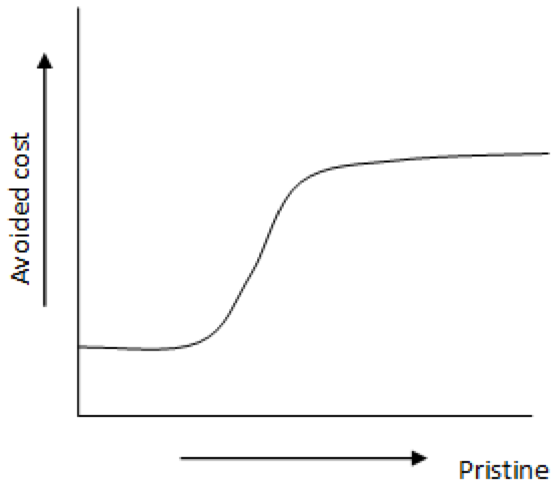

A/U	Benefit	Characteristic	Relationship Justification	Graph
			ammonium), erosion, infiltration), **freshwater** (water table) **land** (gradient), **atmosphere** (temperature, rainfall and wind); **pressures** (management practices e.g. grazing, drainage gripping, burning)]<br><br>**Given the uncertainty of the relationships, this has been upgraded to major and will therefore be taken forward for further consideration in the risk register.**	
MMH	Wildlife	Quantity	The relationship is considered to be +L (major).<br><br>There is the possibility of decreasing and to some extent increasing the quantity (although small) of some of the MMH subcomponents (blanket bogs and heaths) through land use changes e.g. afforestation, expansion of enclosed farmland.<br><br>The quantity will be important in determining the size of a population that can be supported i.e. carrying capacity. As the area increases, although only at the margins, there is potential to increase the abundance of species (note that quality is probably more important in determining the variety of species).	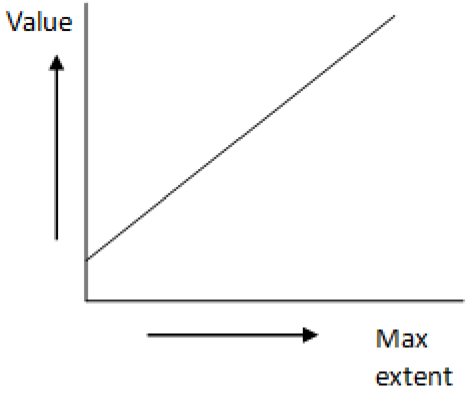
		Quality	The relationship is considered to be +NL (major).<br><br>'Pristine' MMHs are those which have a high level of heterogeneity, supporting a mosaic of habitats. These in turn support a range of highly specialised species.<br><br>It is considered that people will highly value habitats	

A/U	Benefit	Characteristic	Relationship Justification	Graph
			nearing 'pristine' condition and although the contribution of additional species may only be of marginal value after this, the value will still increase.<br><br>It is considered that land management practices can be both beneficial for wildlife, or adversely affect the wildlife. For example, a transition from heather to grass has been observed following an increase in pressure from sheep-grazing with consequences for plant diversity. Sheep preferentially graze grasses but utilise heather and other dwarf shrubs along the edge of grass patches and paths (Palmer *et al*. 2003). Consequently, the condition of heather can be severely impacted by grazers and ultimately leads to grass- dominance across hill slopes (UK NEA, pg 116). However, low intensity grazing could also be beneficial in reducing scrub succession.<br><br>Wildlife = f [**species**; **ecological communities** (pollination), **soils** (pH, nutrient concentrations (TOC, nitrate, phosphate, ammonium), erosion, infiltration), **freshwater** (water table); **land** (altitude, gradient, topography), **atmosphere** (temperature, rainfall, CO_2, N);**material capital** (management practices e.g. grazing, drainage gripping, burning)]	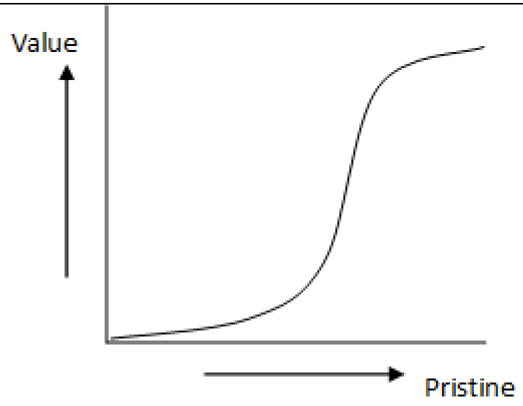
MMH	Equable climate	Quality	The relationship is considered to be +NL (major).<br><br>Blanket bog will only form under certain conditions. If you graze or burn blanket bog, the species required for peat formation, and those which store carbon, i.e. sphagnum moss, will be lost by the resultant lowering of the water table and drying of the existing peat layer. The vegetation	

A/U	Benefit	Characteristic	Relationship Justification	Graph
			will be replaced by heath species typical of drier conditions.<br><br>Suitable land management is therefore crucial in determining whether peat will continue to form and sequester carbon, or whether the locked carbon could be released through poor management.<br><br>Peat formation = f [**species** (sphagnum moss); **ecological communities** (photosynthesis and carbon locking); **soils** (high acidity, organic matter and water holding capacity, nutrient availability); **atmosphere** (temperatures, rainfall, CO_2, N); **freshwater** (high water table); **land** (low gradient); **pressures** (extraction methods, land management - burning and grazing regimes)]	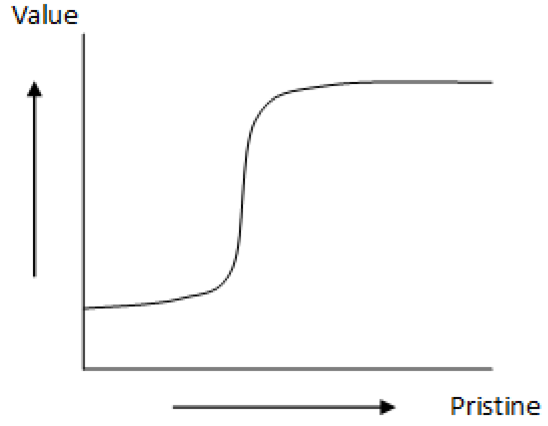
EF	Food	Quantity	The relationship is considered to be +NL (major).<br><br>52.1% of land area in England is EF, consisting of 30.4% arable and horticultural and 21.7% improved grassland (Carey et al. 2008) (UK NEA, pg 200). The EF A/U is therefore one of the main contributors to food production.<br><br>The quantity of the A/U can be increased, and as this does, so does the potential to produce food. However, this will be limited by the market demand and therefore there will	

A/U	Benefit	Characteristic	Relationship Justification	Graph
			be critical area required, after which increases in area will no longer be as highly valued.	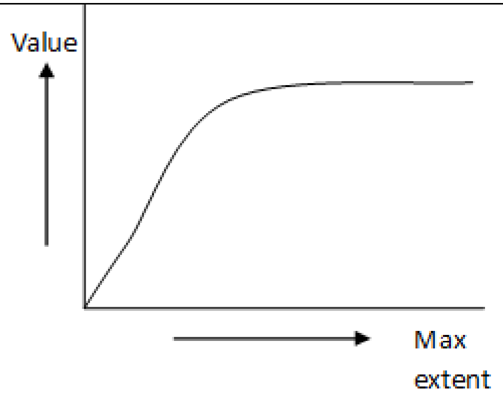
		Quality	The relationship is considered to be +NL (major).<br><br>The amount of food that can be produced from enclosed farmland will depend upon the quality of the habitat, but more importantly the material capital investment made to produce food. Poor quality habitats will not produce large amounts of food and therefore the value will be low. As this increases, the value will significantly increase up to an optimal point, where after further improvements in quality will no longer give such substantial increases in benefit value.<br><br>Most improved grassland is managed to provide food for livestock, mainly sheep and beef and dairy cattle. It is typically in the form of ‘improved’ pasture or long-term leys, managed using herbicides, fertilisers, ploughing, reseeding, liming and drainage to favour competitive, nitrogen-responsive grasses which provide silage to feed livestock over the winter and grazing for the rest of the	

A/U	Benefit	Characteristic	Relationship Justification	Graph
			year (Fuller 1987) (UK NEA, pg 201)<br><br>Crop pollinators - Key driver is the loss of flower-rich, semi-natural landscape elements in farmland (Tscharntke et al. 2005; Winfree et al. 2009; Le Féon et al. 2010) such as flower-rich field margins, species-rich meadows and arable plants in crops. The loss of grass and clover leys, and the legumes they contain, has also been important (Carvell et al. 2006), and pesticides have been shown to have lethal and sub-lethal effects on bees (Morandin et al. 2005), resulting in local losses in bee diversity (Brittain et al. 2010) (UK NEA, pg 218).<br><br>Crop yield = f [**species** (crop type); **soils** (agricultural Grade I – V, erosion); **land** (aspect, altitude, gradient, exposure to wind); **atmosphere** (temperature, rainfall), **freshwater** (groundwater); **minerals** (potassium, magnesium); **ecological communities** (pollination, invasive species/disease); **material capital** (management practices e.g. irrigation, pest/disease control, nutrient enrichment, aeration of soil, crop rotation, GM crops)]<br><br>Livestock = f [**soils**; **species** (grass, cows, sheep, pigs etc); **land** (altitude, gradient); **atmosphere** (rainfall, temperature); **minerals** (potassium, magnesium); **material capital** (re-sowing, nutrient enrichment, breeding stock selection)]	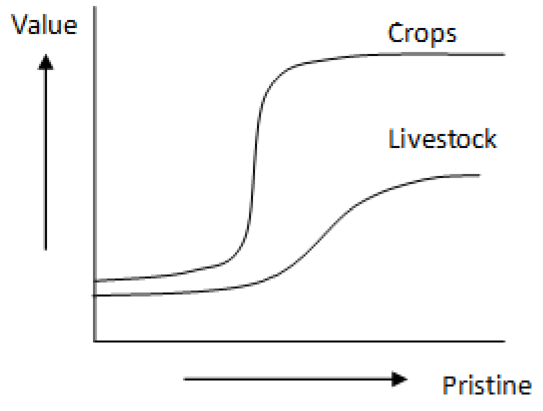

A/U	Benefit	Characteristic	Relationship Justification	Graph
EF	Clean water	Quantity	The relationship is considered to be -NL (major).<br><br>EF habitats cover 60% of England (SoNE, 2008). Agriculture accounts for about 60% of nitrates in rivers (Hunt et al. 2004) and, consequently, influences coastal water quality and fisheries (EEA 2001). Agriculture was responsible for 28% of the damage to rivers due to phosphorous and 61% due to nitrogen in 2012 (Defra, 2013c). It also contributes to approximately 75% of sediment getting into watercourses (Reducing and controlling agricultural pollution, Defra website). A third of waterbodies are at risk from eroded soil (Environment Agency, Corporate Strategy 2010-2015).<br><br>Under baseline quality conditions, as the area of enclosed farmland increases the detrimental effect to water quality will also increase and this is considered to be significant.	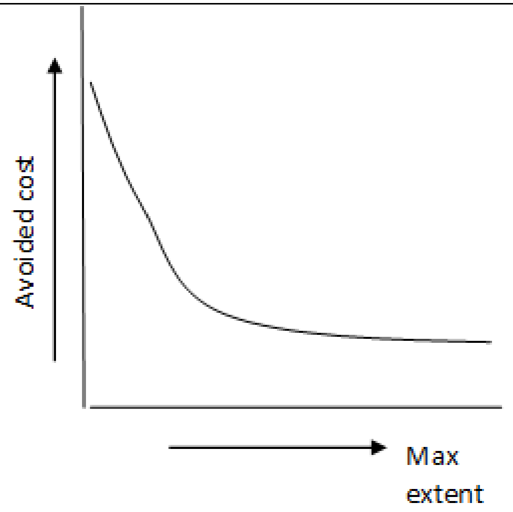

A/U	Benefit	Characteristic	Relationship Justification	Graph
		Quality	The relationship is considered to be +NL (major).<br><br>The quality of EF can significantly affect clean water through improvements in water quality e.g. through use of buffer strips to capture pollutants before they enter watercourses, reduced application of fertilisers will reduce nutrient enrichment of watercourses.<br><br>Water quality = f [**species**; **ecological communities** (pollination, pollutant uptake); **soils** (exposure); **freshwater** (temperature, suspended sediment); **land** (aspect, altitude, gradient, exposure to wind); **atmosphere** (temperature, rainfall, wind); **material capital** (management practices e.g. use of buffer strips, reduced application of fertilisers, ploughing direction to reduce soil erosion, crop rotation to maximise uptake of nutrients for different plant species)]	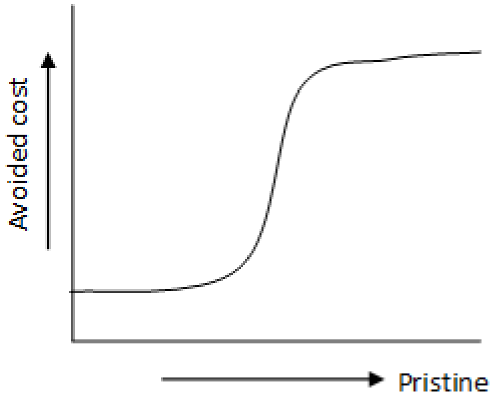
EF	Hazard protection	Quality	The relationship is considered to be +NL (major).<br><br>The quality of the habitat is considered to affect the potential for flooding downstream, with degraded habitats being affected by soil erosion which can be transported to the river networks by surface water runoff.<br><br>Agriculture contributes to approximately 75% of sediment getting into watercourses (Reducing and controlling agricultural pollution, Defra website). A third of waterbodies are at risk from eroded soil (Environment Agency, Corporate Strategy 2010-2015).	

A/U	Benefit	Characteristic	Relationship Justification	Graph
			Soil erosion = f [**species**; **ecological communities** (pollination); **soils**; **land** (aspect, altitude, gradient, exposure to wind); **atmosphere** (temperature, rainfall, wind); **material capital** (management practices e.g. use of buffer strips, ploughing direction to reduce soil erosion, field drainage)]	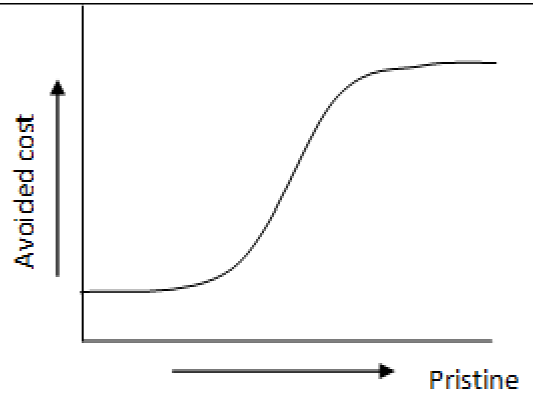
EF	Wildlife	Quantity	The relationship is considered to be +NL (major).<br><br>EF habitats cover 60% of England (SoNE, 2008). Under baseline quality conditions, as the area of enclosed farmland increases the detrimental effect to wildlife will also increase and are likely to be significant.	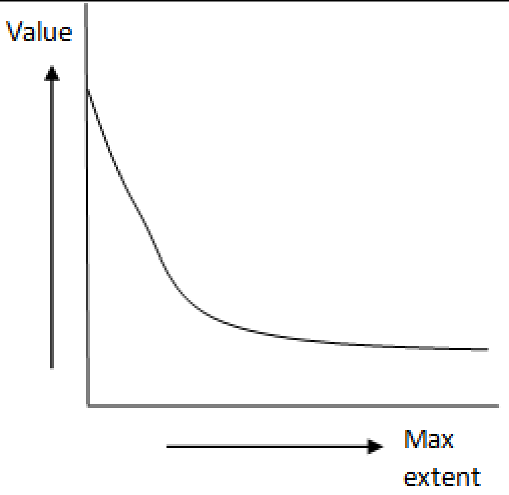

A/U	Benefit	Characteristic	Relationship Justification	Graph
		Quality	The relationship is considered to be +NL (major).<br><br>The quality of the habitat can significantly affect the wildlife value e.g. using buffer strips, set aside lands, increase number of waterbodies on land, reduce use of pesticides, reduce monoculture farming.<br><br>There has been a catastrophic decline in the distribution of arable flowering plants during the last half century and they are now amongst the most threatened elements of our flora (Smith 1989; Rich & Woodruff 1996; Sutcliffe & Kay 2000; Wilson & King 2000; Preston et al. 2002b) (SoNE, 2008) By 2000, the numbers of specialist farmland birds had fallen to 40% of their 1970 levels, and they have fallen a further 4% since then (UK NEA, pg 199).<br><br>Wildlife = f [**species**; **ecological communities** (pollination); **soils**; **land**; **atmosphere**; **material capital** (management practices e.g. use of buffer strips, set aside schemes, creation of waterbodies, reduction in pesticide application, reduction in monoculture)]	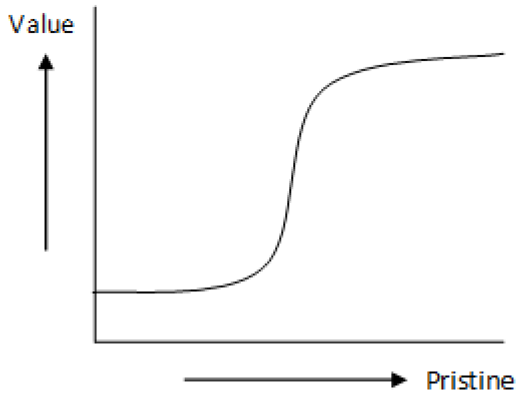

A/U	Benefit	Characteristic	Relationship Justification	Graph
		Spatial configuration	The relationship is considered to be +NL (major).<br><br>EF and hedgerow connectivity is important in allowing wildlife to move not only around the EF A/U but between other A/Us that are separated by EF e.g. woodland, semi-natural grassland.	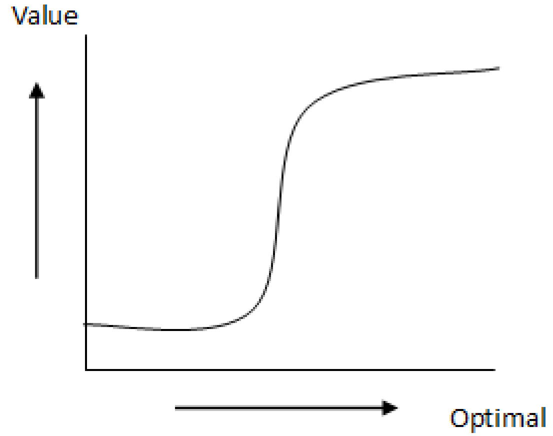
EF	Equable climate	Quality	The relationship is considered to be +NL (major).<br><br>UK agriculture generates net greenhouse gas emissions, with emissions from agriculture accounting for around 7.0% of the UK total (although variation between countries) - nitrous oxide (53% of total agriculture emissions in 2008) and methane (38% of total agriculture	

A/U	Benefit	Characteristic	Relationship Justification	Graph
			emissions in 2008).<br><br>To improve equable climate, a greater proportion of crops should be grown compared to livestock, thereby taking in CO_2 and reducing methane emissions. However, the realisation of this change in quality would be driven by a change in consumer demand which would constrain change.<br><br>To reduce NOx emissions, changes should be made to the amount of fertiliser applied and timing, manure left on soils, etc which affect emissions. Grassland soils are important carbon stores and the level of tillage can affect the amount of carbon released.<br><br>The emissions can be driven by the number of livestock animals, the characteristics of those animals (i.e. their breed, size, yield, digestive systems, etc.), what livestock are fed (for example, a diet with a higher maize content can maintain animal performance while decreasing the production of methane), and how manures are managed (CCC 2010).<br><br>There is potential to reduce greenhouse gas emissions from arable systems through improved soil, fertiliser and agrochemical management (Smith et al. 2008; Macleod et al. 2010). Nitrous oxide emissions arising from crops and soils can be decreased by good nutrient planning, including improving efficiency in using fertiliser by, for example, taking full account of nitrogen in manure applications, timing applications to match crop requirements, using	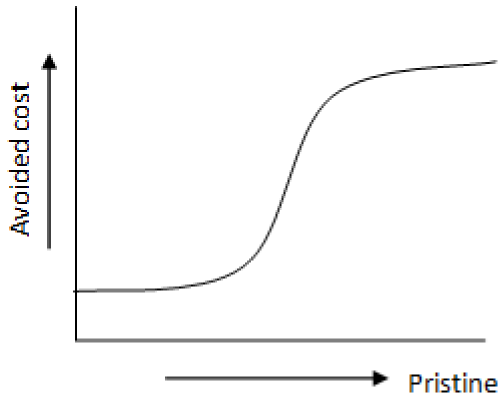

A/U	Benefit	Characteristic	Relationship Justification	Graph
			composts and straw-based manures in preference to slurry where practical, and separating slurry and mineral nitrogen application (UK NEA, pg 216).<br><br>Equable climate = f [**species** (arable vs livestock, livestock breed); **soils**; **material capital** (management practices - tillage, fertilisers, breed selection, stocking densities)]	
SNG	Aesthetics	Quality	The relationship is considered to be +NL (major).<br><br>The quality of SNG can affect the 'sense of experience' gained from the A/U. Although the value of SNG for aesthetics will largely be determined by the land form which cannot be influenced by society, appropriate management of the habitats e.g. removal of scrub could be considered as enhancing views, with degraded habitats not offering the same 'scenery' as good or pristine habitats. (Note, the aesthetic appeal of special plant and animal life is considered under the 'wildlife' benefit)<br><br>The initial unit of semi-natural grasslands will be valued, and as the quality improves, this will increase. However, after a certain level of improvements to the habitat, any additions are no longer as valued i.e. the marginal increase is less.<br><br>Aesthetics = f [**land** (topography, altitude); **material capital** (management practices - grazing, scrub clearance)]	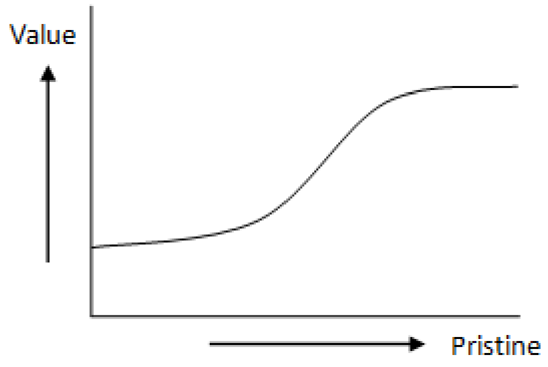

A/U	Benefit	Characteristic	Relationship Justification	Graph
		Spatial configuration	The relationship is considered to be +NL (major).<br><br>Although the spatial configuration of the A/U cannot be easily changed by human management as it is generally defined by geology, the extent of it, and therefore aesthetic appeal, can be interrupted if the landscape is fragmented by urban area, tall structures or other land uses i.e. anything that interrupts the view. A continuous landscape is considered to be highly valued for aesthetics.	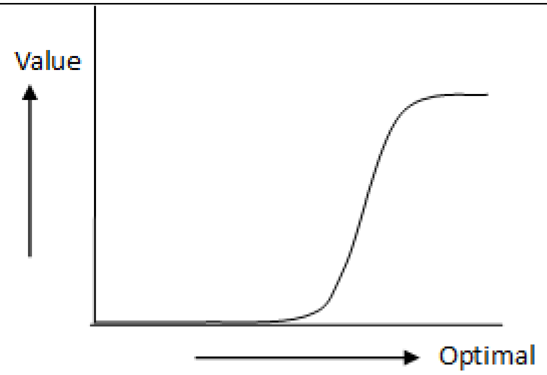
SNG	Wildlife	Quantity	The relationship is considered to be +NL (major).<br><br>It is assumed that all SNG could be replaced with another A/U however the potential to increase the extent of the A/U is considered to be limited as it is dependent on underlying geological conditions. Therefore any changes in extent will be small.<br><br>The quantity will be important in determining the size of a population that can be supported i.e. carrying capacity. As the area increases, although only at the margins, there is potential to increase the abundance of species (note that quality is probably more important in determining the variety of species).	

A/U	Benefit	Characteristic	Relationship Justification	Graph
				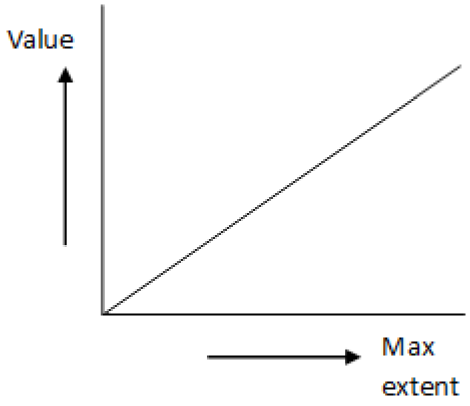
		Quality	The relationship is considered to be +NL (major)<br><br>Lowland grassland priority habitats (dry acid and calcareous grasslands, lowland meadows, purple moor-grass and rush pastures) are home to 206 UK BAP species, while upland grassland priority habitats (calcareous grasslands and upland hay meadows) are home to 41 (UK NEA).<br><br>The main adverse factors affecting SSSI condition are undergrazing, poorly timed grazing and lack of scrub control. These factors allow increased dominance of rank grasses and scrub at the expense of more desirable but less competitive species (SoNE Report, 2008)<br><br>Wildlife = f [**species** (high diversity); **ecological communities** (pollination); **soils**; **land** (topography); **atmosphere** (rain, temperature); **material capital**	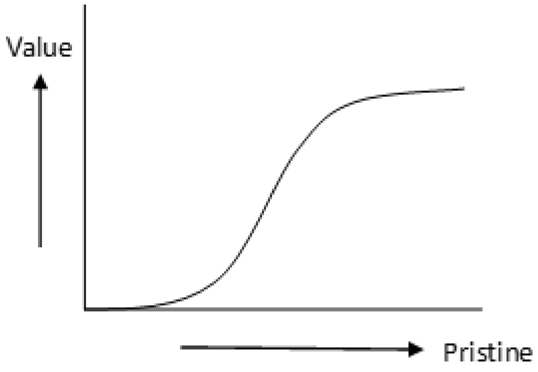

A/U	Benefit	Characteristic	Relationship Justification	Graph
			(conservation management - grazing, cutting, scrub management)]	
SNG	Equable climate	Quality	The relationship is considered to be +NL (major) (uncertain)<br><br>The Countryside Survey (Carey et al. 2007) estimates, and accounting for their land cover, that acid and neutral grasslands contain 144 Tg and 149 Tg, respectively, of the UK carbon store in the top 15 cm soil layer (Chamberlain *et al*. 2010). These figures account for 21% of the soil carbon across the Countryside Survey broad habitats (UK NEA, pg 181).<br><br>Poor management of the habitats could lead to a release of this stored carbon e.g. soil erosion. However, it is unclear how quickly carbon would be released - carbon stock is in the upper 15cm of soil.<br><br>Carbon storage = f [**ecological communities**; **soils**; **land** (topography, exposure) **atmosphere** (temperature, rainfall, CO_2, SO_2); **pressures** (grazing, cutting, scrub management)]<br><br>**Given the uncertainty of the relationship, this has been upgraded to major and will therefore be taken forward for further consideration in the risk register.**	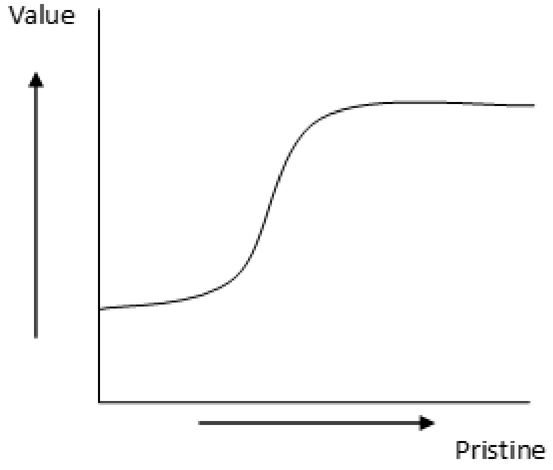
W	Fibre	Quantity	The relationship is considered to be +NL (major).<br><br>The quantity of the A/U can be changed, with the potential for all areas of woodland to be converted to a different land use. Therefore the first unit of woodland will be highly	

A/U	Benefit	Characteristic	Relationship Justification	Graph
			valued. However, there will be a point where the area of woodland satisfies market demand, and any increase in quantity thereafter will not be as valued.<br><br>The quantity of woodland is considered to be the determining factor in amount of time produced and therefore changes in quantity of the A/U will be significant.	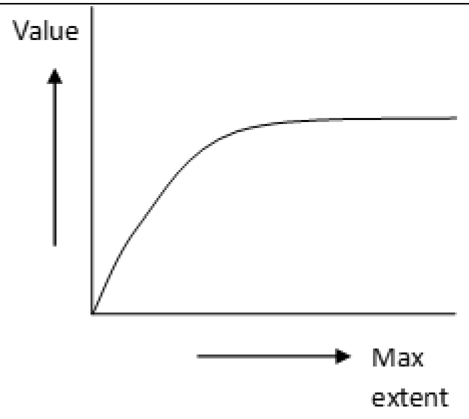
		Quality	The relationship is considered to be +NL (major).<br><br>The quality of the A/U can affect the yield of timber. In a poor quality environment e.g. high levels of acidification, low nutrient cycling, tree growth will be poor and therefore limit yield. It is considered that the costs and effort in harvesting this poor growth would considerably outweigh the value, and in these circumstances it is considered that timber would not be harvested.<br><br>As the quality of the A/U increases, the potential for tree growth also increases. It may be possible to select the species that are grown, to select those with quickest growth rates. Improvements in material capital to harvest the timber will also increase the amount of output. However, there will be critical point where after further increases in quality will produce marginal benefits.	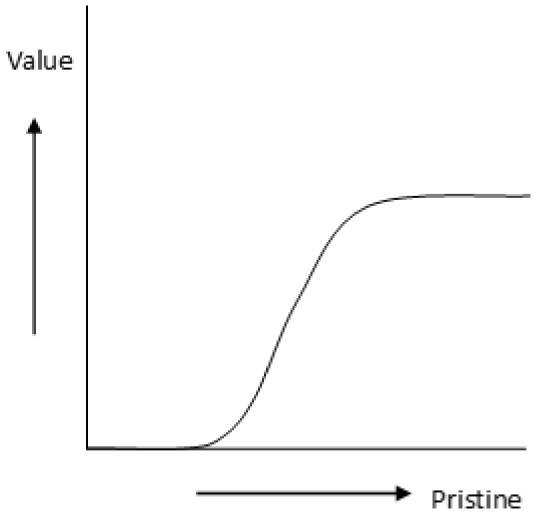

A/U	Benefit	Characteristic	Relationship Justification	Graph
			Timber yield = f [**species** (hardwood and softwood), **ecological communities** (invasives, pests and disease); **soils** (decomposers, nitrifying bacteria - nitrogen fixation, nutrient cycling); **freshwaters** (groundwater); **land** (altitude, gradient); **atmosphere** (rain, temperature, nitrogen, carbon dioxide, wind); **minerals** (potassium, magnesium); **material capital** (management - coppicing, felling, crop rotation, irrigation, processing timber, machinery transport, pest control, nutrient enrichment, pollution - SO_2)]	
W	Clean water	Quantity	The relationship is considered to be NL (major) (uncertain)<br><br>No woodland is likely to mean a high level of run-off, erosion and soil failure which would have a significant negative effect on water quality. Benefits are gained quickly once some woodland is there (binding soils), with benefits slowing down once the woodland is planted.<br><br>Woodland provides a purification role by intercepting pollution, and reduces sediment inputs to watercourses (requirement to remove suspended sediment).<br><br>It is unclear how woodland effects the yield of water. It is likely that woodland would reduce the quantity of water available downstream through evapotranspiration, and indirect effect with water held by roots/interception of foliage with trees present. The effect on total yield is uncertain.<br><br>**Given the uncertainty of the relationship, this has been**	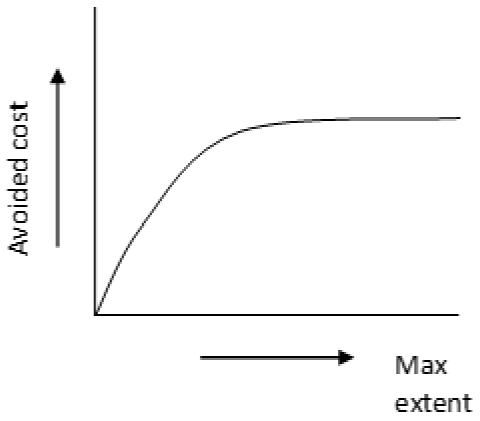

A/U	Benefit	Characteristic	Relationship Justification	Graph
			**upgraded to major and will therefore be taken forward for further consideration in the risk register.**	
		Spatial configuration	The relationship is considered to be +NL (major).<br><br>The afforestation of uplands, which are a significant source of water (quantity) could adversely affect the amount of clean water obtainable. Afforestation in lowland areas, around towns, could act as interceptors to pollution before it reaches the watercourse.<br><br>The potential for spatial configuration of woodlands to affect clean water benefit (quality and quantity) is therefore considered to be significant.	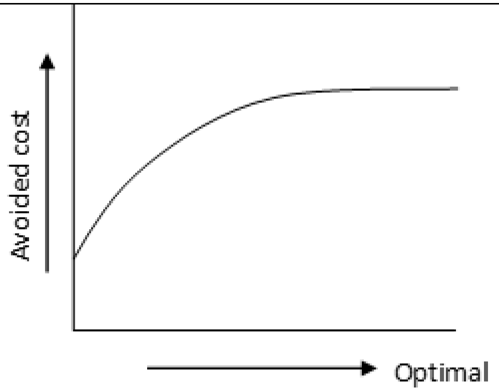
W	Clean air	Quantity	The relationship is considered to be +NL (major).<br><br>Woodland can absorb pollutants internally or adsorb pollutants externally on to leaf and bark surfaces, and provides an overall role in production of 0_2 required for the air we breathe (UK NEA 2011).<br><br>As the quantity of woodland increases, the ability to clean air also increases, although this is most highly valued with the initial units of woodland. There is no critical mass with regard the potential for woodlands to clean air, and therefore this will keep increasing.	

A/U	Benefit	Characteristic	Relationship Justification	Graph
				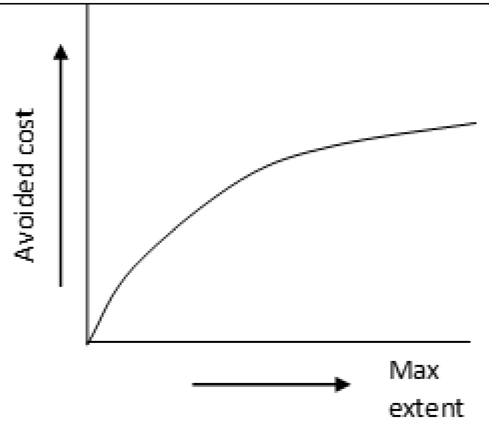
W	Recreation	Quantity	The relationship is considered to be +NL (major).<br><br>Woodland is listed as one of the most popular destinations for countryside visits (~250 million day visits per year) (UK NEA 2011, pg 268)<br><br>It is assumed that all woodland could be replaced with another A/U therefore the value crosses the origin. The first units of woodland will be highly valued, however, at a critical amount i.e. enough woodland to satisfy recreational demands, any increases over this will not be as valued.	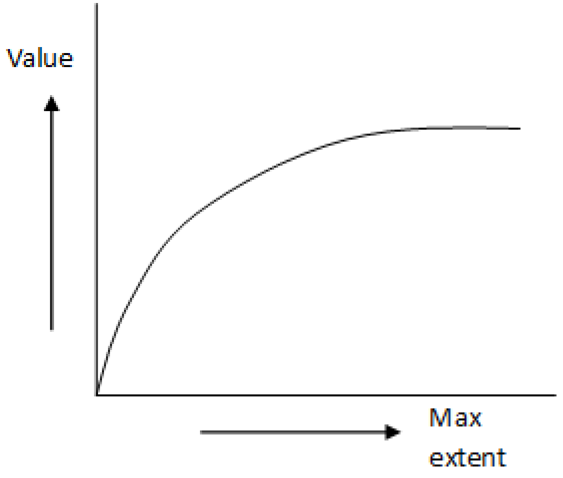

A/U	Benefit	Characteristic	Relationship Justification	Graph
		Spatial configuration	The relationship is considered to be +NL (major).<br><br>Only 55% of population have access to woods greater than 20ha within 4km, and 10% have access to woods greater than 2ha within 500m of their home (UK NEA 2011, pg 268)<br><br>Woodland as recreational resource will be more valued when close to populations. However, there will be a certain distance which the majority of people will be happy to travel, and this will be highly valued, with anything closer being valued but with only marginal benefits.	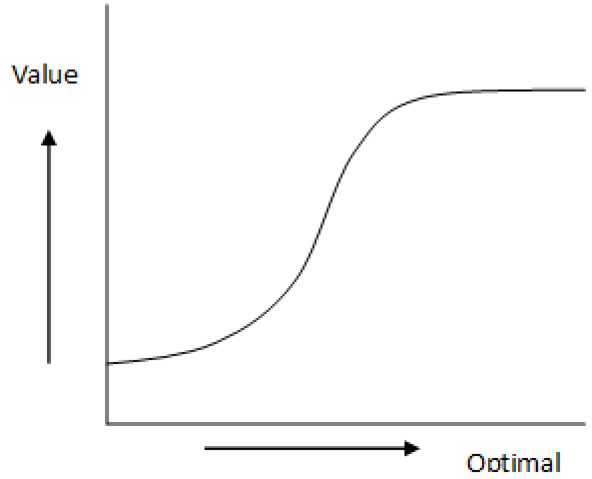

A/U	Benefit	Characteristic	Relationship Justification	Graph
W	Aesthetics	Quantity	The relationship is considered to be +NL (major).<br><br>It is assumed that all woodland could be replaced with another A/U therefore the value crosses the origin. The first units of woodland will be highly valued, however, at a critical amount i.e. enough woodland to satisfy recreational demands, any increases over this will not be as valued.	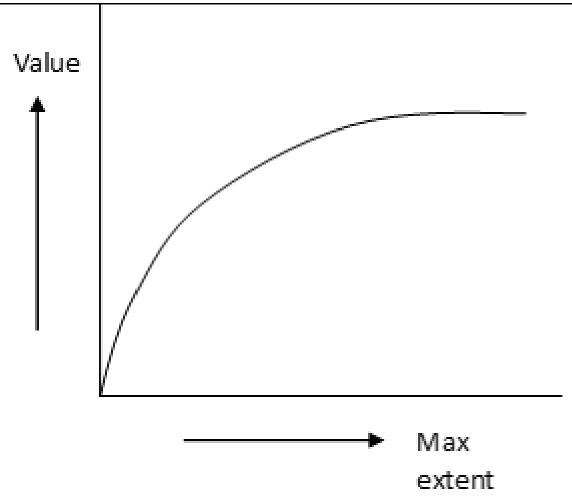

A/U	Benefit	Characteristic	Relationship Justification	Graph
		Quality	The relationship is considered to be +NL (major)<br><br>There is some association between perceptions of landscape value and woodland characteristics: for example, woodland type (broadleaves tend to be more favoured than conifers), tree age (large, old trees tend to be favoured over young ones), openness (valued more than dense, closed areas) and diversity (mixtures and variation valued over uniformity) (Willis *et al*. 2003) (UK NEA, pg 269).<br><br>Ancient woodlands and veteran trees are historic features in their own right and provide a link to past society and culture (Rackham 2013). Many 'Royal Forests' have hundreds of years of history, tradition, myth and legend associated with them, helping to create important historic landscapes. Ancient woodland is also increasingly appreciated for its archaeological content.<br><br>The initial unit of woodlands will be valued, and as the quality improves, this will increase. However, after a certain level of improvements to the habitat, any additions are no longer as valued i.e. the marginal increase is less.<br><br>Aesthetics = f [**species** (broadleaved vs coniferous, varied age structure); **land**; **material capital** (management practices - coppicing, felling)]	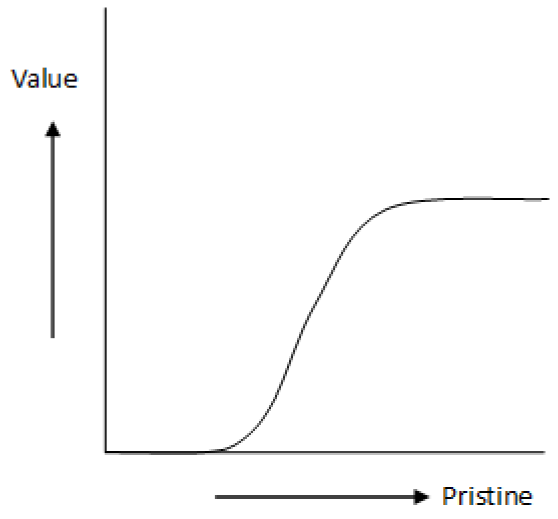

A/U	Benefit	Characteristic	Relationship Justification	Graph
		Spatial configuration	The relationship is considered to be +NL (major).<br><br>There is greater aesthetic value of woodlands where there are numerous plots in the landscape rather than a continuous belt. There is also considered to be greater value if people have views of woodland from their properties. Society has the potential to significantly influence the location of new woodland creation over the next 25 years.	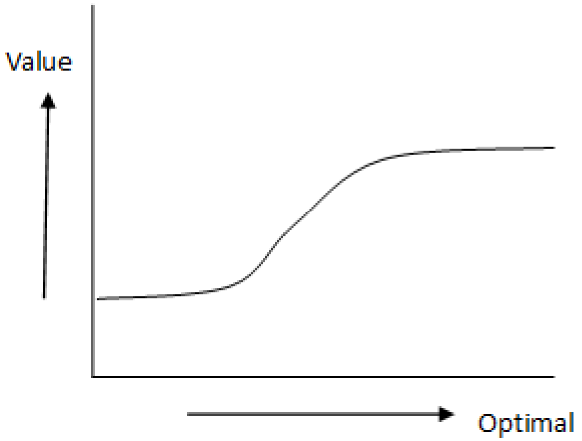
W	Hazard protection	Quantity	The relationship is considered to be +NL (major).<br><br>Woodland provides protection from flooding and soil failure by regulating the quantity of water downstream and stopping soil erosion.<br><br>Forests and woodland have long been associated with an ability to slow down run-off and reduce downstream flooding. There are three ways that trees can assist flood risk management; by reducing the volume of runoff, by promoting rainfall infiltration into the soil and reducing the rate of runoff, and by delaying the downstream passage of flood flows.<br><br>As woodland cover in a catchment increases, the avoided cost of protection will also increase. However, there will be a critical point after which any further increases in area	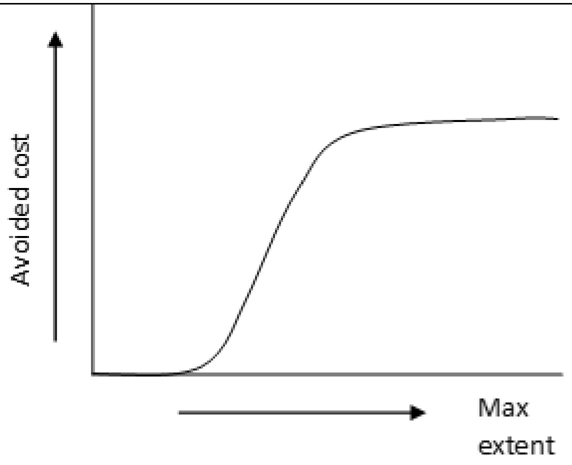

A/U	Benefit	Characteristic	Relationship Justification	Graph
			will only have a marginal increase in benefits.	
		Spatial configuration	The relationship is considered to be +NL (major).<br><br>It is important to locate woodland in an appropriate area of the catchment to maximise influence on flooding, through interception of rainfall and regulate base flows.<br><br>Woodland in upland parts of the catchment are therefore considered optimal, whilst extensive areas on floodplains may be considered non-optimal due to the effect of water displacement.	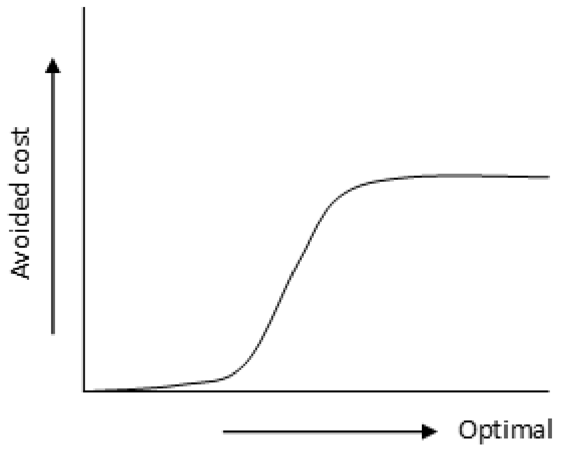

A/U	Benefit	Characteristic	Relationship Justification	Graph
W	Wildlife	Quantity	The relationship is considered to be +NL (major).<br><br>The quantity of the A/U can be changed, with the potential for all areas of woodland to be converted to a different land use. Assuming baseline quality of the A/U, the first unit will be highly valued, and this will continue to increase with the area of woodland. However, there will be a point where the area of woodland is sufficient, but the quality of the habitat will limit species diversity and abundance, and any increase in quantity thereafter will not be as valued.<br><br>The quantity will be important in determining the size of a population that can be supported i.e. carrying capacity. As the area increases, although only at the margins, there is potential to increase the abundance of species (note that quality is probably more important in determining the variety of species).	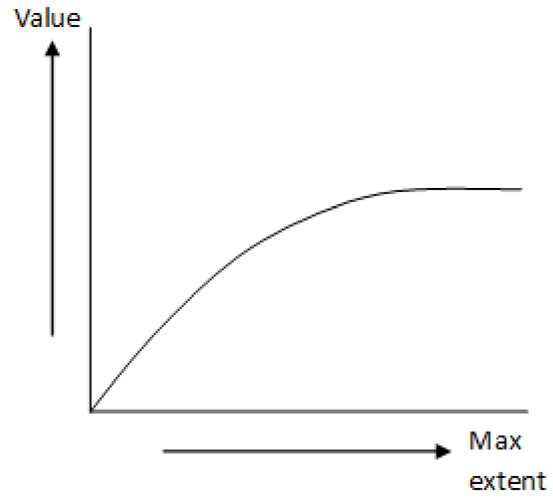

A/U	Benefit	Characteristic	Relationship Justification	Graph
		Quality	The relationship is considered to be +NL (major).<br><br>As the quality of the habitat increases, the potential to support a range of species and high abundance of species will increase. Improvements in diversity will be attributable to management e.g. coppicing and felling, dead log piles, as well as decreases in pollution and pests. A certain level of species diversity and abundance will be highly valued, however after this, increases in more specialised species or general abundance will be of lowering value.<br><br>Wildlife value = f [**species** (diversity), **ecological communities** (invasives, pests and disease); **soils** (decomposers, nitrifying bacteria - nitrogen fixation, nutrient cycling); **freshwaters** (groundwater); **land** (altitude, gradient); **atmosphere** (rain, temperature, nitrogen, carbon dioxide, wind); **minerals** (potassium, magnesium); **material capital** (management -coppicing, felling, restocking with native species, dead log piles, pest control); **pressures** (pollution - SO_2)]	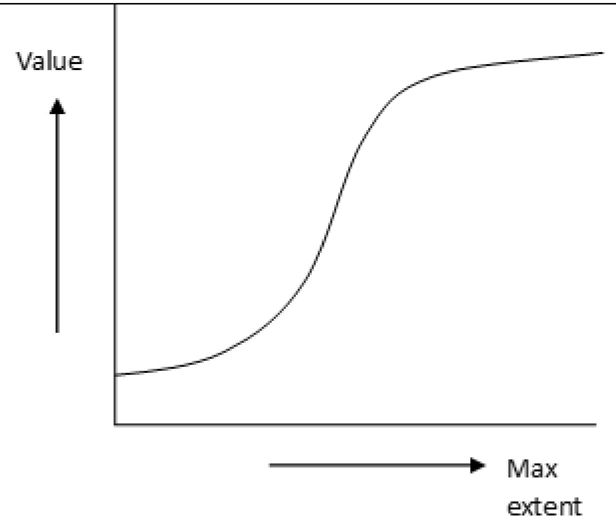

A/U	Benefit	Characteristic	Relationship Justification	Graph
		Spatial configuration	This relationship is considered to be +NL (major).<br><br>With a higher connectivity of woodlands, the species diversity and abundance will increase. It is also possible to create woodland closer to people, and therefore the recreational and aesthetic value of wildlife could increase when in closer proximity to people.	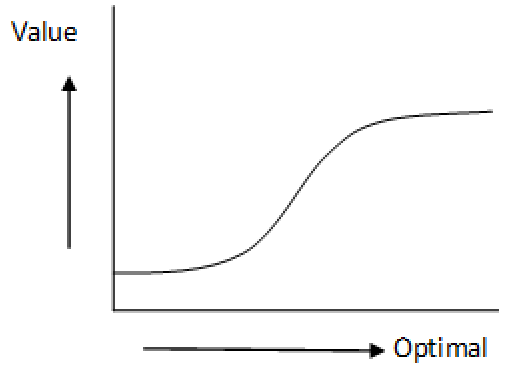
W	Equable climate	Quantity	The relationship is considered to be +L (major).<br><br>Woodland is a carbon store, taking up and locking carbon dioxide through photosynthesis. With no woodland, there would be no benefit to equable climate. However, as the area of the woodland increases, the potential to store carbon would also increase (assuming wood is not used as a biofuel).	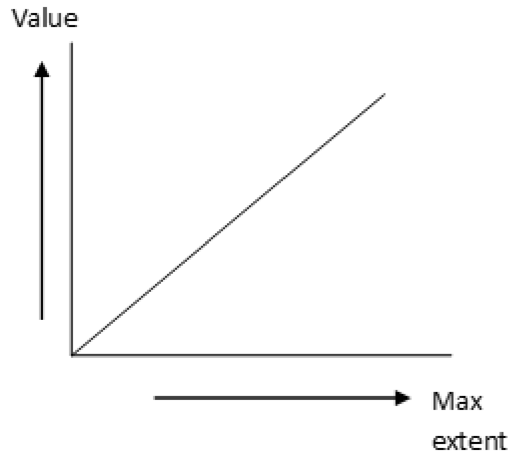

A/U	Benefit	Characteristic	Relationship Justification	Graph
FW	Clean water	Quantity<br><br>Wetlands	The relationship is considered to be +NL (major)<br><br>Wetland habitats have a role in purification - they trap and filter particulates. Wetland systems, particularly reedbeds, have combinations of highly oxic and anoxic sites within their soils due to stratification in the sediment or soil profile and/or the release of oxygen from plant roots; these conditions are conducive to the breakdown and transformation of many pollutants including organic and inorganic compounds derived from agriculture and denitrification (a major mechanism for ‘cleaning’ groundwaters of their nitrogen content).<br><br>An increase in wetlands could therefore significantly improve clean water quality.	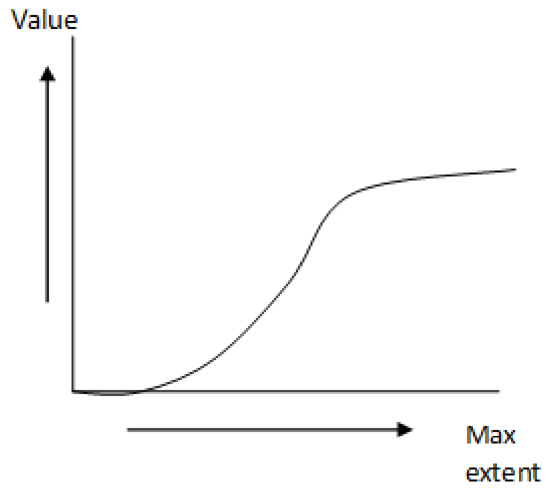
		Quality	The relationship is considered to be +NL (major).	

A/U	Benefit	Characteristic	Relationship Justification	Graph
				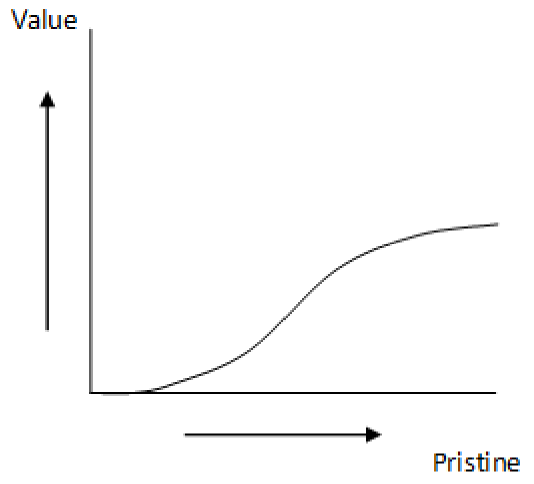
FW	Recreation	Quality	The relationship is considered to be +NL (major).<br><br>Quality can be affected by access restrictions e.g. reservoir not open to public, angling season reduced, low volume of water e.g. drought conditions which limits potential for kayaking etc, and water quality which could deter contact recreation e.g. swimming, angling, or habitat degradation which could restrict walking opportunities.<br><br>In degraded/poor environment (e.g. no water, no fish), there would be no recreation opportunities. As the quality of freshwater increases the value will also increase. However, this will only be up to a certain level of improvements and after this the marginal increase in value will become less.	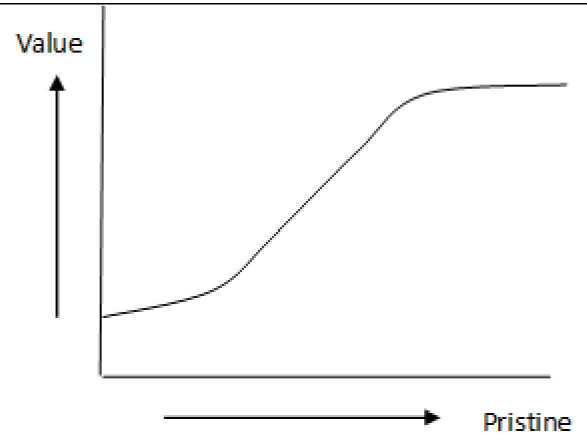

A/U	Benefit	Characteristic	Relationship Justification	Graph
			Recreation = f [**freshwater** (water - volume, flow velocity, nutrients, bacteria, aquatic vegetation), **land** (gradient, altitude), **species** (fish), **material capital** (access, signage/waymarks)]	
FW	Aesthetics	Quality	The relationship is considered to be +NL (major).<br><br>The degradation of habitats is considered to be of key importance when valuing the aesthetics e.g. heavily modified river channel vs natural meandering channel. As the quality of freshwater increases (i.e. naturalisation of the river channel improves) the value will also increase. However, this will only be up to a certain level of improvements and after this the marginal increase in value will become less.<br><br>Aesthetics = f [**freshwater** (water - volume, flow, nutrients, floodplain connectvity), **land** (gradient, altitude), **species**, **material capital** (pollution e.g. oil, litter, absence of significant modifications)]	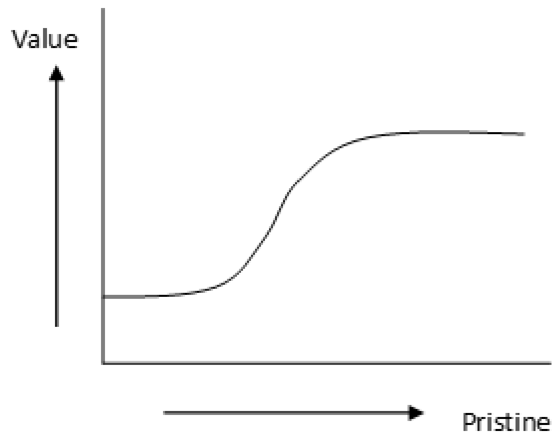
FW	Hazard protection	Quality<br><br>Wetlands (incl. floodplain)	The relationship is considered to be +NL (major).<br><br>In a degraded/poor environment e.g. low permeability of floodplain, the avoided cost of flood protection will be low. However, as the quality increases, the avoided cost will	

A/U	Benefit	Characteristic	Relationship Justification	Graph
			also increase. At a critical point, further improvements will not significantly avoid costs of protection.<br><br>Flood protection = f [**freshwater** (floodplain connectivity, extent and permeability, water - volume, flow velocity, suspended sediment), **land** (gradient), **atmosphere** (rainfall), **species** (woody debris), **material capital** (flow regulation, storage reservoirs, channel modification)]	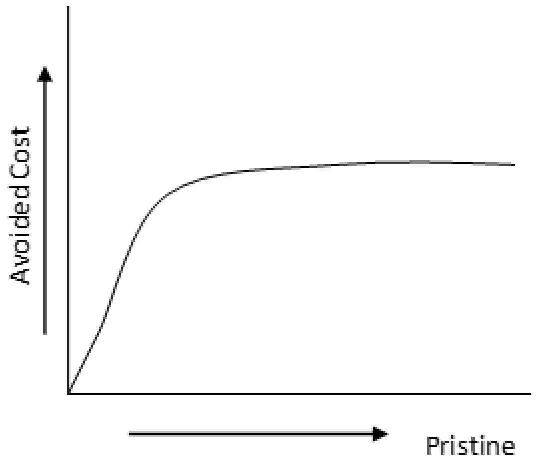
		Spatial configuration<br><br>Wetland (incl. floodplain)	The relationship is considered to be +NL (major).<br><br>Floodplains will be found next to their respective rivers, however their capacity to hold flood waters will be determined by the area available and connectivity with the river. Reservoirs with the aim of regulating flow will need to be optimally positioned in relation to the population they are protecting.<br><br>Therefore optimal positioning of the wetland and standing open water subcomponents will have a positive impact on avoided flood protection costs. This is considered to be significant.<br><br>The quantity will be important in determining the size of a	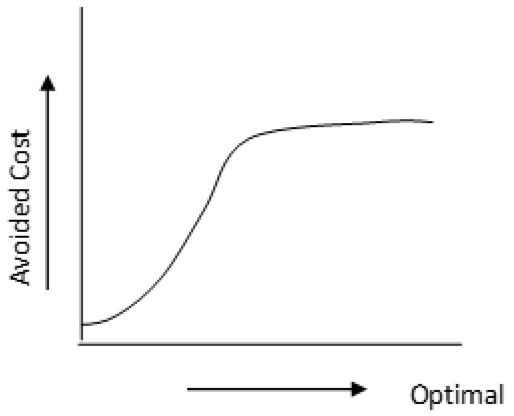

A/U	Benefit	Characteristic	Relationship Justification	Graph
			population that can be supported i.e. carrying capacity. As the area increases, although only at the margins, there is potential to increase the abundance of species (note that quality is probably more important in determining the variety of species).	
FW	Wildlife	Quantity<br><br>Wetlands	The relationship is considered to be +NL (major).<br><br>It is considered that you cannot significantly change the area of the A/U, just around the margins such as the wetland subcomponent. These changes in area will however, give rise to a significant change in wildlife value.	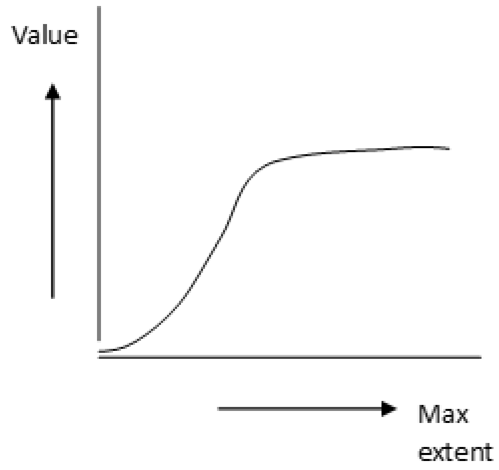
		Quality<br><br>Rivers and streams<br>Wetlands	The relationship is considered to be +NL (major).<br><br>As the quality of freshwater increases the value will also increase. However, this will only be up to a certain level of improvements and after this the marginal increase in value will become less.<br><br>The degradation of habitats is considered to be of key importance when valuing the wildlife benefit e.g. heavily	

A/U	Benefit	Characteristic	Relationship Justification	Graph
			modified river channel vs natural meandering channel.<br><br>Wetlands are very sensitive to subtle changes in water supply and quality, including acidity, nutrient levels and water table fluctuations (Wheeler & Shaw 2001) (UK NEA, pg 332)<br><br>Wildlife = f [**freshwater** (water - volume, flow, nutrients, floodplain connectivity, suspended sediment, nutrient levels, acidity, groundwater); **land** (gradient, altitude); **species** (woody debris); **pressures** (pollution e.g. oil, litter, flow regulation, channel modification)]	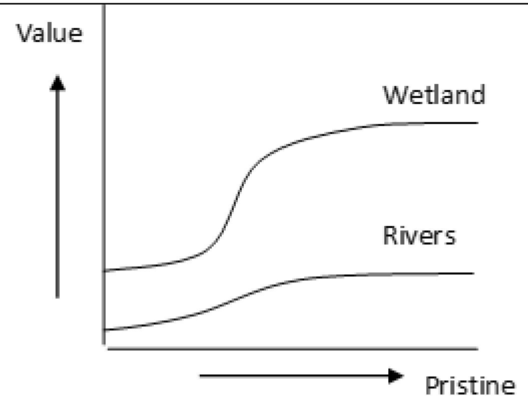
		Spatial configuration	The relationship is considered to be +NL (major).<br><br>With a higher connectivity of freshwater habitats, the species diversity and abundance will increase. It is also possible to create some freshwater habitats closer to people, although limited e.g. new waterbodies, however it is considered that the recreational and aesthetic value of wildlife could increase when in closer proximity to people.	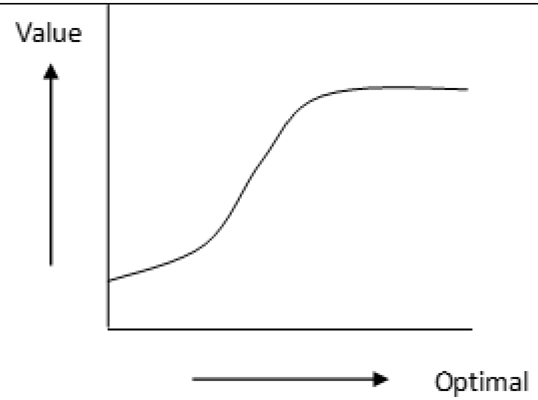
FW	Equable climate	Quantity<br><br>Wetlands	The relationship is considered to be +NL (major).<br><br>Natural England (2010) estimated that the remaining lowland fen in English peatlands stored 1,004–2,576 tonnes	

A/U	Benefit	Characteristic	Relationship Justification	Graph
			of carbon/ha, and raised bog peats stored 1,575–1,629 tonnes of carbon/ha (UK NEA, pg 310). Therefore change in quality of these systems could increase release of carbon.<br><br>It is considered that you cannot significantly change the area of the A/U, just around the margins such as the wetland subcomponent (e.g. through afforestation, conversion to enclosed farmland). These changes in area will however, give rise to a change in equable climate value i.e. more wetland area, more potential to sequester carbon (overall low when compared to other A/Us e.g. marine) and greater area of open water/rivers and streams the more potential there is to moderate extreme temperatures and cool urban areas.	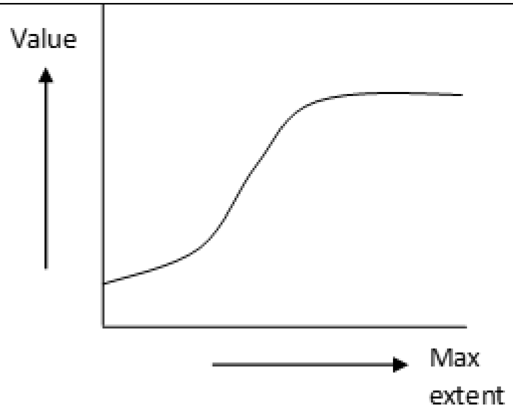
		Quality	The relationship is considered to be +NL (major)<br><br>Plankton biomass takes in carbon which is then locked in sediment. The health of the plankton community may govern diversity and biomass, and therefore amount of carbon uptake.<br><br>Natural England (2010) estimated that the remaining lowland fen in English peatlands stored 1,004–2,576 tonnes of carbon/ha, and raised bog peats stored 1,575–1,629 tonnes of carbon/ha (UK NEA, pg 310). Therefore change in quality of these systems could increase release of carbon.<br><br>Carbon sequestration = f [**species** (plankton biomass); **freshwater** (water - volume, flow, nutrients, floodplain connectivity, suspended sediment, nutrient levels, acidity,	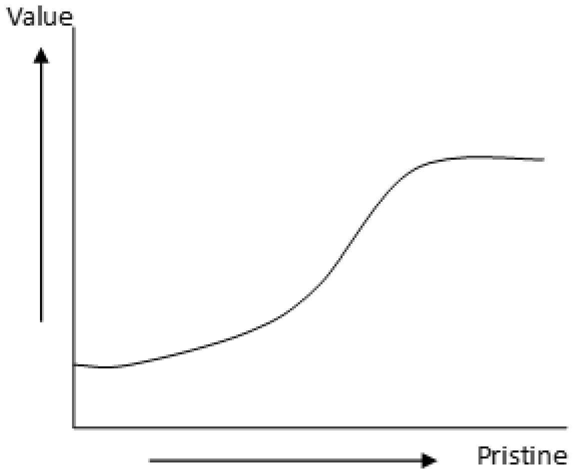

A/U	Benefit	Characteristic	Relationship Justification	Graph
			groundwater); **land** (gradient, altitude); **atmosphere** (temperature); **pressures** (pollution e.g. oil, flow regulation, channel modification)]	
U	Clean water	Quantity	The relationship is considered to be -NL (major).<br><br>The built urban subcomponent of the A/U is considered to have a detrimental effect on clean water. Urban rivers are typically the receiving waterbodies for sewage treatment plant effluent and stormwater discharge. As the area of the built urban environment increases the potential for pollution incidents increases and therefore the potential for avoided treatment cost will decrease.	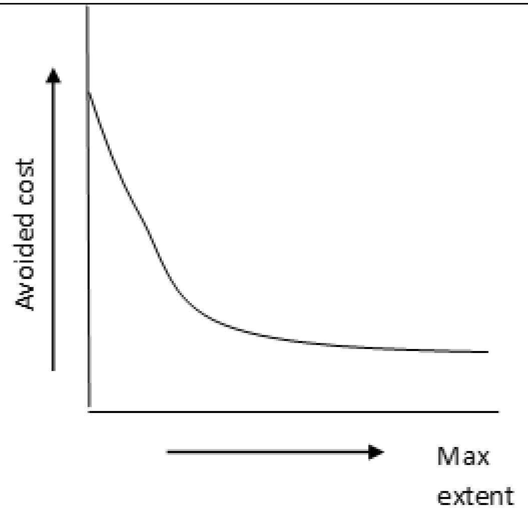

A/U	Benefit	Characteristic	Relationship Justification	Graph
U	Clean air	Quantity	The relationship is considered to be -NL (major).<br><br>As with clean water, the built urban subcomponent of the A/U is considered to have a detrimental effect on clean air.<br><br>With an increase in urban extent, it is assumed that there is an associated increase in population size, and therefore an increase in vehicle emissions, emissions from plant associated with residential/office/ retail space (e.g. CHP plants) and a proportionate increase in construction (dust) as the extent of the urban area increases - PM_{10}, NO_x etc.	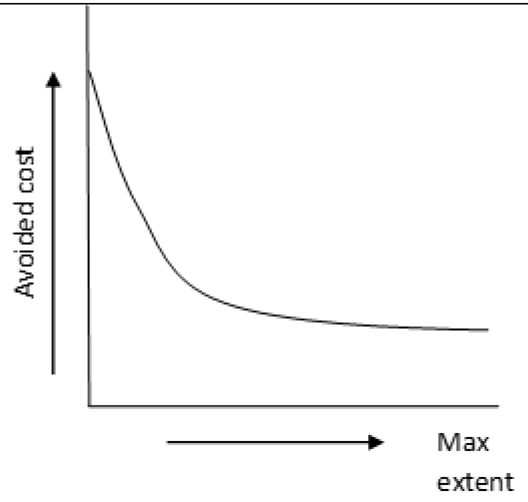
		Quality	The relationship is considered to be +NL (major)<br><br>The built urban subcomponent of the A/U is considered to have a detrimental effect on clean air through its contribution to pollution - PM_{10}, NO_x, CO_2 etc. However policy drivers can help to reduce impacts by targeting improvements in air quality (Low Emission Zones), and urban greenspaces with trees can scavenge air pollutants.<br><br>= f [**species** (London plane trees); **atmosphere**; **material capital** (policies to cap emissions - PM_{10}, NO_2, $SO_{2,}$ reduction in car useage, proportion of green space to built urban)]	

A/U	Benefit	Characteristic	Relationship Justification	Graph
				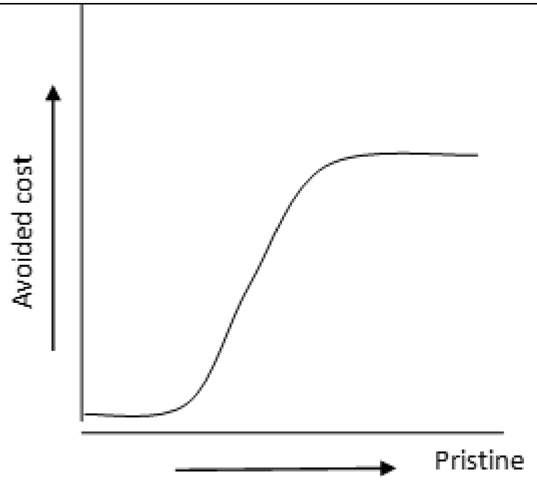
U	Recreation	Quantity<br><br>Greenspace	The relationship is considered to be +NL (major).<br><br>For this characteristic we are considering the amount of greenspace available within the urban environment. This is not considered to be substitutable with other greenspace (i.e. other A/U such as woodland, semi-natural grasslands).<br><br>The initial unit of greenspace within the urban will be highly valued, and the relationship will follow the case of diminishing returns as the amount of greenspace increases i.e. there will be a critical amount of greenspace that is valued the most, and after this there will only be slight increases in value.	

A/U	Benefit	Characteristic	Relationship Justification	Graph
				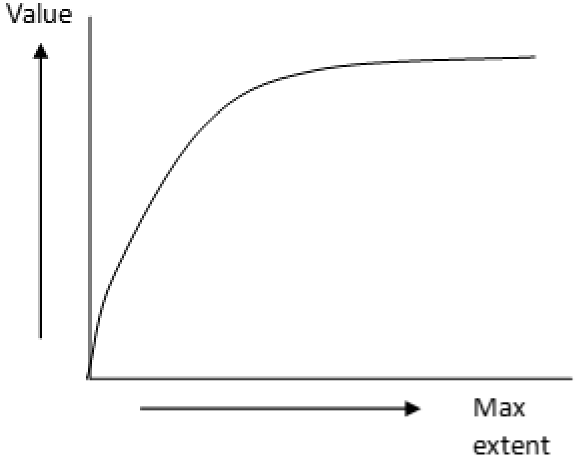
		Spatial configuration	The relationship is considered to be +NL (major).<br><br>The value of a greenspace is highly dependent upon its proximity to the population which uses it. It is also considered that new green spaces could be positioned in an area of high population densities to maximise value. However, this is not considered to be an increasing positive relationship - there will be an optimal distance, and after this other limiting factors will come into play e.g. quality.	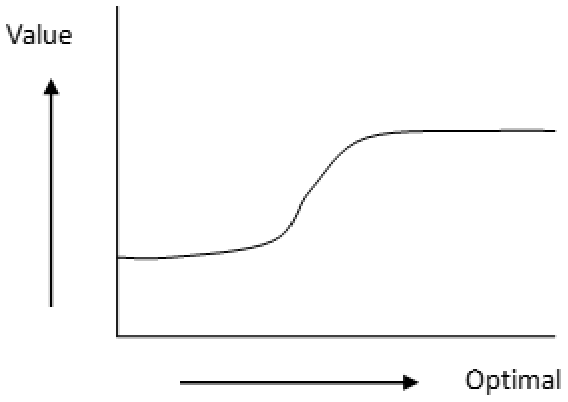

A/U	Benefit	Characteristic	Relationship Justification	Graph
U	Aesthetic	Quantity	The relationship is considered to be +NL (major).<br><br>For this characteristic we are considering the amount of greenspace available within the urban environment. This is not considered to be substitutable with other greenspace (i.e. other accounting units such as woodland, semi-natural grasslands).<br><br>The initial unit of greenspace within the urban will be highly valued there will be a critical amount of greenspace that is valued the most, and after this there will only be slight increases in value as quantity increases.	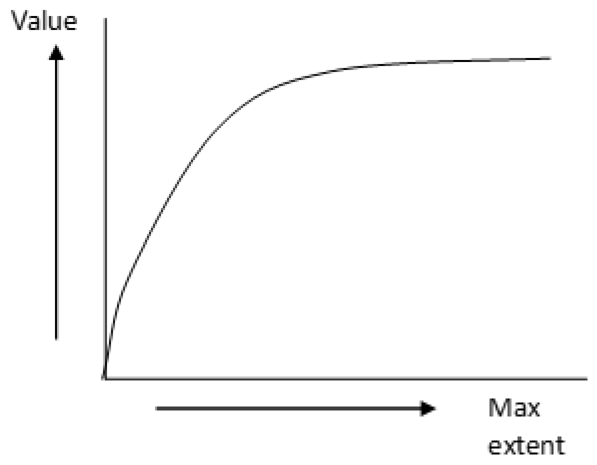
		Quality	The relationship is considered to be +NL (major).<br><br>Poor quality greenspace is considered to be that which is dominated by litter, graffiti, and visually unattractive. It is considered that some urban greenspaces may be too small to have significant recreational value, but do have aesthetic value (Forest Research, undated).<br><br>The initial unit of greenspace within the urban environment will be highly valued, and as the quality improves, this will increase. However, after a certain level of improvements, any additions are no longer as valued i.e. the marginal increase is less.	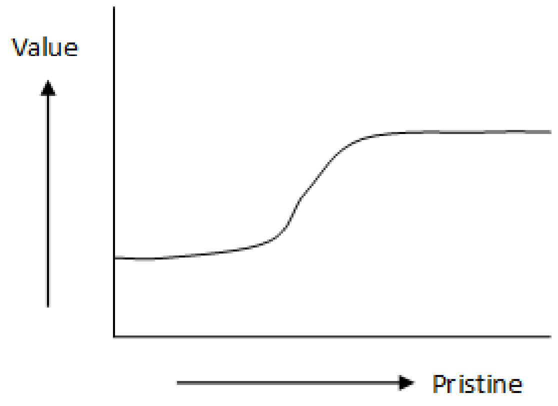

A/U	Benefit	Characteristic	Relationship Justification	Graph
			= f [**ecological communities** (urban greenspace - parks, gardens, towpaths), **material capital** (litter, graffiti management practices e.g. stocking of plants, maintenance of trees)]	
		Spatial configuration	The relationship is considered to be +L (major).<br><br>The aesthetic value of a greenspace is derived from proximity to a population and ability to see green space. Therefore optimal positioning of urban green space will be in densely populated areas to maximise the number of people who can see it.	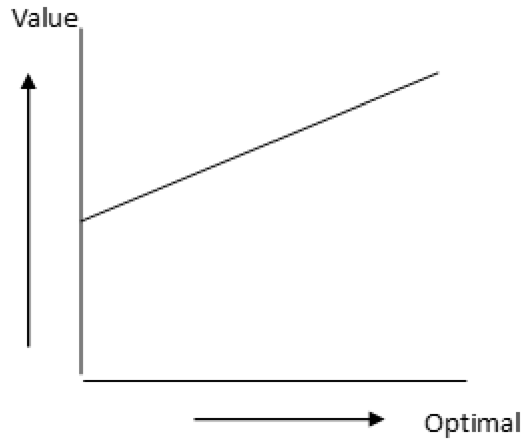

A/U	Benefit	Characteristic	Relationship Justification	Graph
U	Hazard protection	Quantity	The relationship is considered to be -NL (major).<br><br>Surface water flooding is a key issue in urban areas due to the extent of impermeable surfaces (concrete, compacted soils). Therefore as the quantity of urban area increases (assuming baseline quantity), the cost of implementing effective flood protection would also increase. The impact of increased urban areas is considered to be significant.	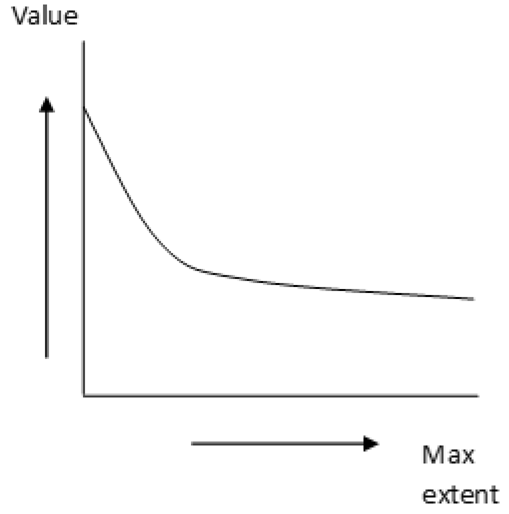
U	Wildlife	Quantity	The relationship is considered to be -NL (major).<br><br>The built urban subcomponent of the A/U is considered to have a detrimental effect on wildlife, whilst an increase in urban greenspace would be considered as a positive for the wildlife benefit. However, it is considered that the rate of urbanisation and expansion of the built urban subcomponent outweighs any increases in greenspace and therefore the overall effect is negative.	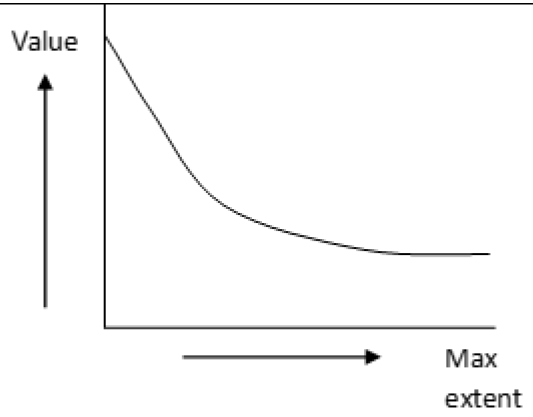

A/U	Benefit	Characteristic	Relationship Justification	Graph
		Quality	The relationship is considered to be +NL (major).<br><br>The quality of the urban environment can be improved by increasing the area and quality of the urban greenspaces as species such as birds and mammals have minimum area thresholds in order to survive. To improve wildlife in an urban area, measures should be undertaken to promote native species rather than ornamentals, set aside areas of grassland to be allowed to grow up/increase wildflowers, remove alien/invasive species, pick up litter etc.<br><br>Wildlife = f [**species** (native); **ecological communities** (pollination); **soils**; **material capital** (green space, management practices e.g. mowing regime altered to create different sward heights, areas for wildflower recovery, removal of alien/invasive species)]	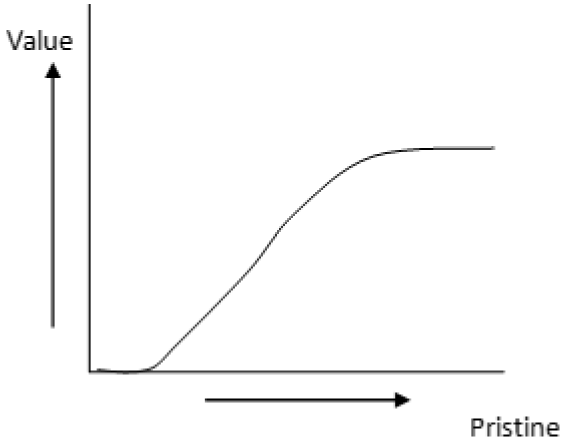
		Spatial configuration	The relationship is considered to be +NL (major).<br><br>To increase abundance and diversity, urban greenspaces should be connected to allow movement of species between areas. Fragmented habitat, and therefore fragmented populations, are less sustainable.	

A/U	Benefit	Characteristic	Relationship Justification	Graph
				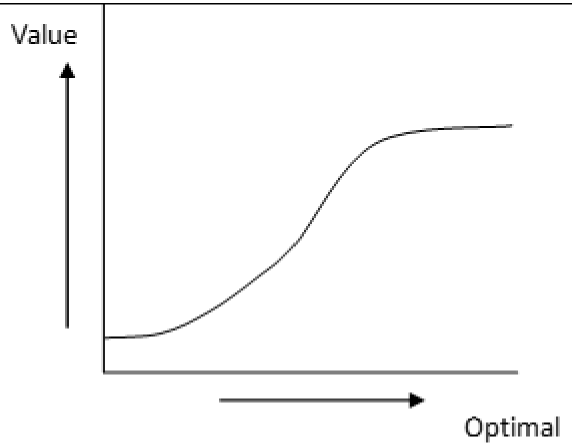
U	Equable climate	Quantity	The relationship is considered to be -NL (major)<br><br>The built urban subcomponent of the A/U is considered to have a detrimental effect on the climate, whilst urban greenspaces are considered to positively effect the climate through CO_2 uptake and heat regulation.<br><br>With an increase in urban extent, it is assumed that there is an associated increase in population size, and therefore an increase in CO_2, ozone emissions, heat islands and wind tunnelling due to an increase in the built element. The cost to treat these effects is also considered to increase as the extent of the urban area increases.<br><br>These impacts are considered to outweigh the benefits of urban greenspace.	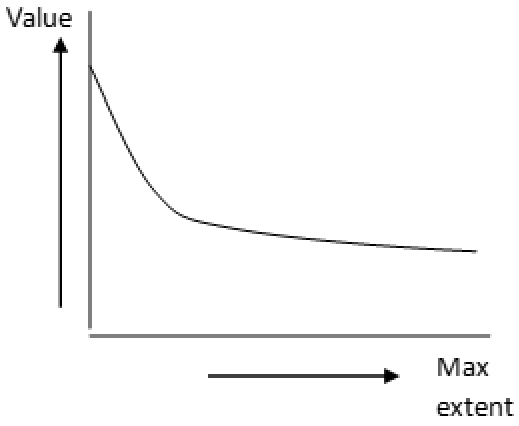

A/U	Benefit	Characteristic	Relationship Justification	Graph
CM	Recreation	Quality	The relationship is considered to be +NL (major).<br><br>The quality of coastal margins could significantly affect the active enjoyment of them e.g. litter, poor bathing water standards.<br><br>The initial unit of coastal margins will be valued, and as the quality improves, this will increase. However, after a certain level of improvements, any additions are no longer as valued i.e. the marginal increase is less.<br><br>Recreation = f [**coasts** (limits on bacteria levels (E.coli and streptococci, limits on levels of cyanobacteria, phytoplankton, macro-algae); **material capital** (management practices e.g. litter collection, maintenance of coastal footpaths, signage/waymarks, maps, information boards, waste bins, toilets, modification for golf courses)]; pressures (industrial, wastewater and sewage related discharges).	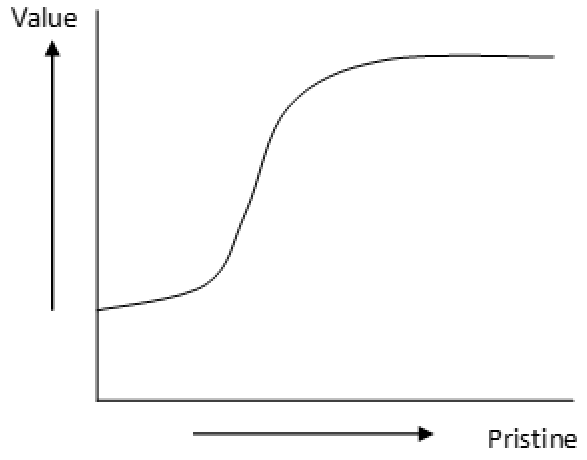

A/U	Benefit	Characteristic	Relationship Justification	Graph
CM	Aesthetic	Quality	The relationship is considered to be +NL (major).<br><br>The quality of coastal margins could significantly affect the enjoyment of them. It is considered that people will value a heterogeneous landscape with good quality habitats and limited hard engineering structures.<br><br>The initial unit of coastal margins will be valued, and as the quality improves, this will increase. However, after a certain level of improvements, any additions are no longer as valued i.e. the marginal increase is less.<br><br>Aesthetics = f [**coasts** (abundance of habitats); **oceans** (view, sense of being at seaside); **ecological communities** (wildlife associated with habitats); **material capital** (hard engineering, cultural memories, archaeology and heritage)]	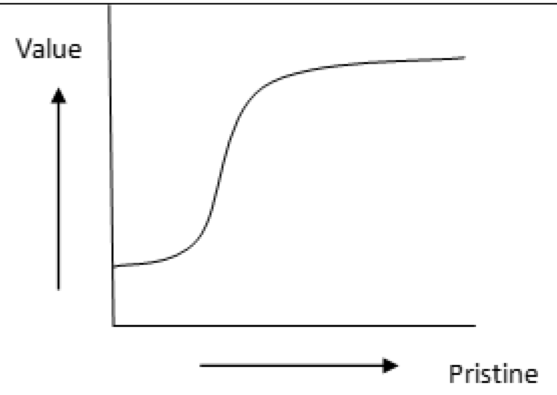
		Spatial configuration	The relationship is considered to be +NL (major).<br><br>The aesthetic appeal can be interrupted if the landscape is fragmented by urban area, tall structures or other land uses i.e. anything that interrupts the view. A continuous and connected landscape is considered to be highly valued for aesthetics.	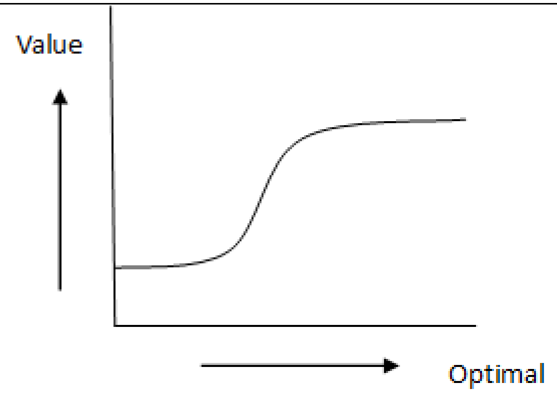

A/U	Benefit	Characteristic	Relationship Justification	Graph
CM	Hazard protection	Quantity	The relationship is considered to be +L (major).<br><br>The coastal margin protects from erosion, wave and tide damage and coastal flooding. Vegetated saltmarsh can attenuate wave energy; an 80m strip can reduce the height of landward seawalls from 12m to 3m (UK NEA Technical Report). Sand dunes and shingle banks dissipate energy and if wide enough, can replace the need for artificial defences. With schemes such as managed realignment, substantial areas of saltmarsh can be created.<br><br>The area of these same habitats can be reduced by change in land use e.g. drainage for enclosed farmland, changes for recreational benefits e.g. golf courses, and also reduced through hard engineering flood defence schemes.<br><br>Therefore the contribution of coastal margins to flood defence is considered to be high, with potentially large increases in area possible which are significantly valued.	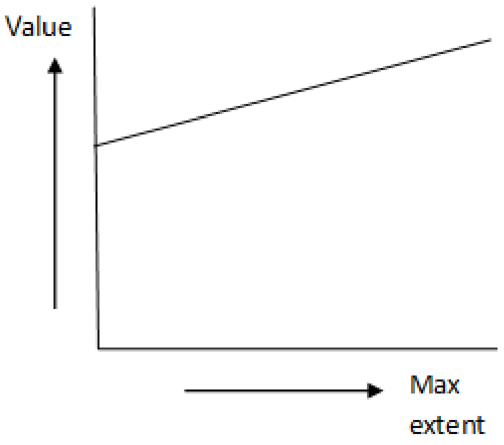

A/U	Benefit	Characteristic	Relationship Justification	Graph
		Quality	The relationship is considered to be +NL (major).<br><br>It is assumed that a poor quality habitat would not have the structural integrity to provide effective flood protection e.g. width not sufficient, pioneer communities with limited ability to bind sediments (dunes and saltmarsh). As discussed in the UK NEA for sand dunes, vegetation cover and root mass bind substrate, promote sand deposition and help to build wider and higher dunes. A poor quality habitat would also be vulnerable to erosion and therefore there is a link between quality and quantity. As the quality increases, the amount spent on manufactured capital flood defence will reduce.<br><br>Although it is considered that the accounting unit can substantially reduce the cost of manufacture capital flood defence, there will always be situations where additional protection is required.<br><br>Protection from hazards = f [**species**; **coasts** (feature is wide and elevated, low creek density (saltmarsh)); **ecological communities** (colonisers such as *Salicornia*, sand dune stabilisers e.g. marram grass, tall and dense vegetation); **freshwater** (sediment); **land** (coastal morphology, aspect); **ocean** (tidal submergence, tidal current velocity, salinity, temperature); **material capital** (hard engineering structures)]	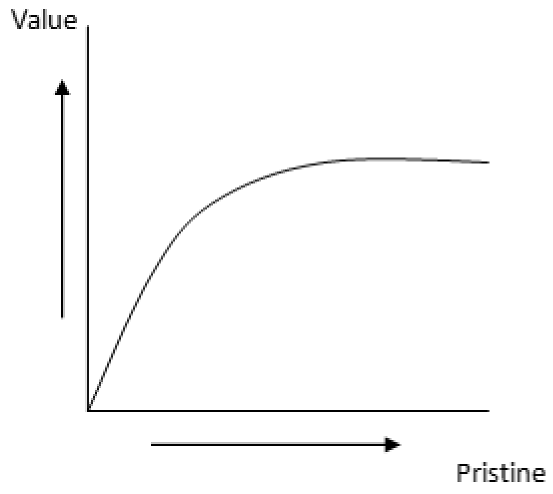

A/U	Benefit	Characteristic	Relationship Justification	Graph
CM	Wildlife	Quantity	The relationship is considered to be +L (major).<br><br>The A/U quantity can be increased and decreased in relation to some of the subcomponent habitats e.g. saltmarsh, sand dunes etc. With schemes such as managed realignment, substantial areas of saltmarsh can be created. The area of these same habitats can be reduced by change in land use e.g. drainage for enclosed farmland, changes for recreational benefits e.g. golf courses.<br><br>Therefore the contribution of coastal margins to wildlife is considered to be high, with increases in area possible which are significantly valued.	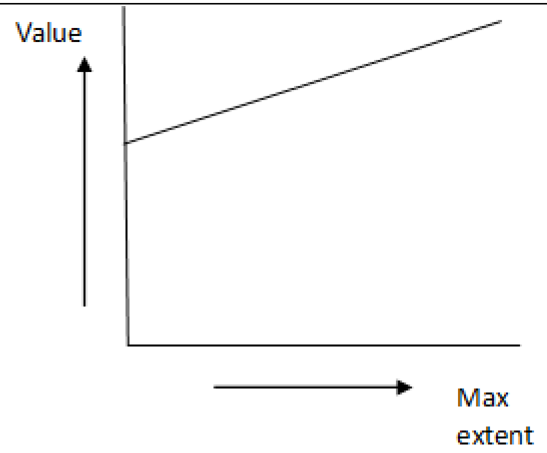
		Quality	The relationship is considered to be +NL (major).<br><br>'Pristine' coastal margins are those which have a high level of heterogenity, supporting a mosaic of habitats, including early successional habitats. These in turn support a range of highly specialised species which can tolerate the harsh conditions (salinity, inundation etc).<br><br>In habitats such as shingle banks, lichen live on the pebbles and therefore any level of disturbance can affect this.<br><br>It is considered that people will highly value habitats nearing 'pristine' and although the contribution of additional species may only be of marginal value after this,	

A/U	Benefit	Characteristic	Relationship Justification	Graph
			the value will still increase.<br><br>Wildlife = f [**species** (specialised, native, range of successional species); **ecological communities** (mosaic of habitats, range of successional stages, maintenance of stable systems); **freshwater** (sediment); **land** (coastal morphology incl. aspect and gradient); **atmosphere** (wind); **oceans** (tidal submergence, water velocity, turbulence, salinity levels, nutrient levels); **coasts** (stable systems, sediment, soil pH); **material capital** (management regimes e.g. light grazing, scrub clearance, lack of disturbance on shingle,)pressures (air pollution -acidification from sulphur and nitrogen deposition))]	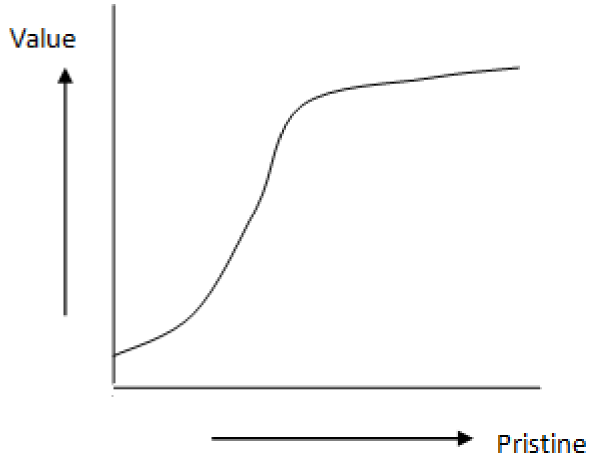
CM	Equable climate	Quantity<br>Saltmarsh<br>Sand dune	The relationship is considered to be +L (major). – MAJOR<br><br>Climate regulation provided by habitats where there is rapid soil development or sediment accumulation (sand dune and saltmarsh). UK saltmarsh have high rates of carbon sequestration storing 0.64-2.19 t c/ha/yr<br><br>The ability to increase the quantity of the accounting unit is limited; only the proportion of subcomponent habitat that could change.<br><br>Therefore the contribution of coastal margins to equable climate (carbon storage in saltmarsh) is considered to be high, with small increases in area that are possible but which are significantly valued.	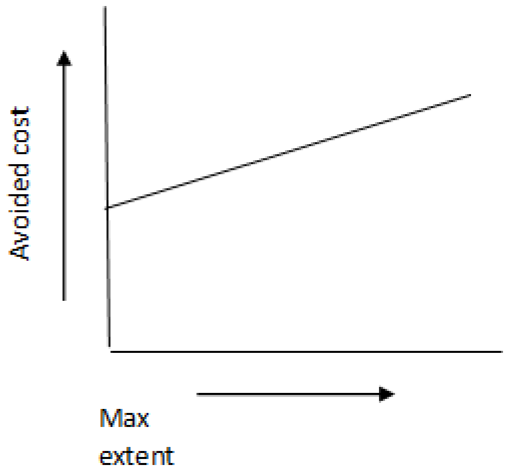

A/U	Benefit	Characteristic	Relationship Justification	Graph
		Quality<br>Saltmarsh<br>Sand dune	The relationship is considered to be +NL (- MAJOR)<br><br>Sand dune and saltmarsh act as carbon sinks, storing carbon as sediment accumulates and soils develop, with early successional systems having a greater potential to store (UK NEA Technical Report). However, these habitats also release methane and NO_x. The net effect on climate regulation is considered to be beneficial, however the overall contribution is limited by the quantity of these habitats. Potential release from degradation however is considered to be significant.<br><br>Carbon storage =f [**coasts** (early successional stages); **atmosphere** (wetter conditions); **ecological communities** (vegetation fixes CO_2)] pressures (development erosion).	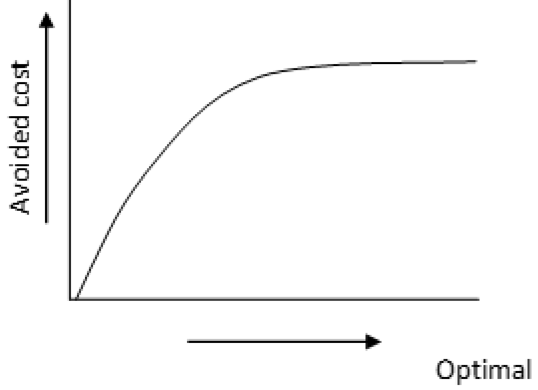
M	Food	Quality	The relationship is considered to be +NL (major).<br><br>The quality of the A/U can affect the yield of fish, shellfish etc. In a poor quality environment e.g. acidified ocean, high salinity levels, low phytoplankton, the numbers of fish etc that could be harvested would be very low, and considerable effort and inputs would be required to harvest this low number.<br><br>As the water quality improves, the system will become	

A/U	Benefit	Characteristic	Relationship Justification	Graph
			more productive and therefore number of fish/shellfish etc that it can support will increase. Similarly the value of the output from the A/U will increase. However, there will be a critical point where after any further increases in quality will produce marginal benefits.<br><br>There is an important link with coasts as saltmarsh provide a nursery ground for fish species.<br><br>Fish/shellfish yield = f [**species** (fish, shellfish), **coasts** (nursery ground for fish species), **atmosphere** (wind), **oceans** (salinity, currents, tides, waves, temperature, pH), **ecological communities** (population regulation, food web dynamics), **land** (morphology), **material capital** (harvesting effort, harvesting preferences - policy driven, equipment)pressures(pollution)].	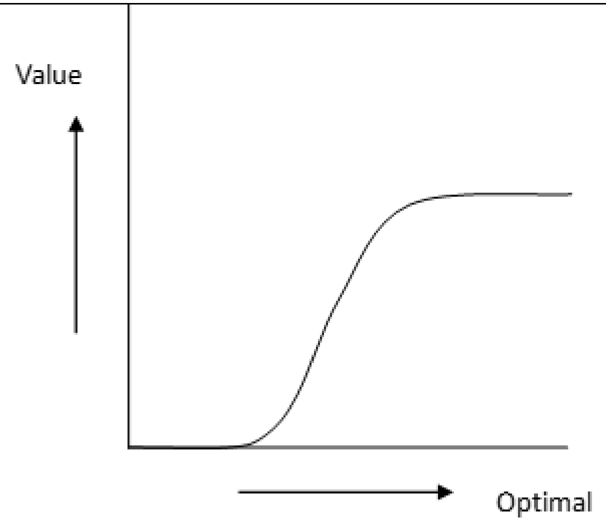
M	Wildlife	Quality	The relationship is considered to be +NL (major)<br><br>The abundance and diversity of marine wildlife will be primarily determined by the quality of the water. This will increase up to a critical point, after which any increase in quality, and therefore associated species diversity and/or abundance, will be less valued.<br><br>Wildlife = f [**species**; **ecological communities** (population regulation, food web dynamics); **land** (topography, elevation); **atmosphere** (wind), **oceans** (salinity, tides, currents, waves, temperature, pH); **pressures**(pollution (e.g. oil spills, sewage effluent), invasive species (e.g. ballast water), fish by-catch, damage to benthic	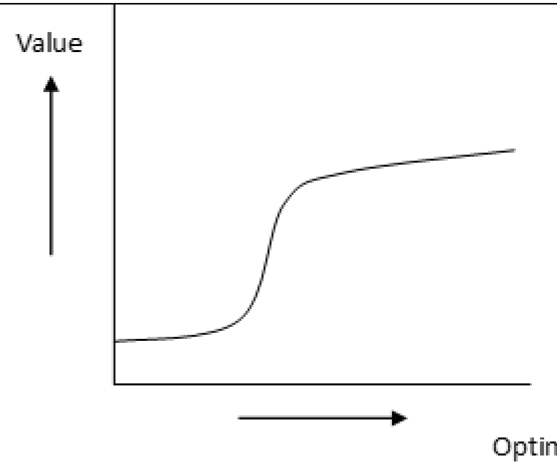

A/U	Benefit	Characteristic	Relationship Justification	Graph
			communities through trawl fishing)].	
