# Supplementary material for: REVIEW: Towards a risk register for natural capital
Source: J Appl Ecol. 2015 Apr 13;52(3):641–53. doi: 10.1111/1365-2664.12431 (PMC4979659; doi:10.1111/1365-2664.12431)
Supplement: Supplementary file 2 — Table S2. Justification for risk register scoring and references used to support the scoring. [file JPE-52-641-s002.pdf]

Supplementary Table 2:

Justification for risk register scoring

We set out here all 73 relationships identified in the first stage of the analysis as being most influential, where there is a link between the quality, quantity or spatial configuration of the Major Habitat type and the flow of benefits. These are the relationships that received a Red, Orange or Green rating according to their risk rating. We start with those relationships rated as ‘A’ and move through to ‘C’. The Table below explains out how the following Tables 1 to 3 can be read.

The R (Red), Amber (A), Green (G) score is shown in the RAG key table (below). Evidence for each assessment is shown in the Table and an Unicertain score for each Status and Trend measurement is estimated (1 to 4). These individual scores are added in the final column to give an overall uncertainty for the RAG rating (Low uncertainty<=4; high uncertainty >=5).

**Major Habitat**	**Benefit**	**Characteristic**	**Current Status**	**Target**	**Trend**	**RAG (A-C)**
		What characteristic of the Habitat are we concerned with? [Quantity, Quantity or Spatial Configuration]<br><br>Quality sets out production functions, with underlying natural capital assets. Those in **red** can be influenced and are important to provision of ES benefit.	What is the status of the relationship relative to a defined target?		What is the trend in the relationship?	**RAG**<br>(Overall RAG based on status and trend)
			RAG rating for Trend		RAG rating for Status	
			Uncertainty of Trend		Uncertainty of Status	Total Uncertainty<br><br>(Summation of Uncertainty)

Uncertainty Key

		Agreement	
		High	*Low*
Robustness	*Significant evidence*	1	3
	Limited evidence	2	4

RAG Key

		Status		
		Above, at or just below target	Below target	Substantially below target (>50%)
Trend	Positive or not discernible	*A*	*B*	*B*
	Negative	*B*	*B*	*C*
	Strongly negative	*C*	*C*	*C*

	High confidence	Low confidence
Low risk	*A*	*A*
High risk (or risk unknown)	*B*	*B*
Very high risk	*C*	*C*

Table 1: RAG status A

Habitat Type	Benefit	Characteristic	Current Status	Target	Trend	RAG (A-C)
Mountains Moors and Heath	Aesthetics	Spatial Configuration	Assumed target met based on Government ability to designate areas to meet societal preferences.		N/A	**A**<br><br>(8)
			(4)		(4)	
Enclosed farmland	Food	Quantity	52.1% of land area in England is EF (Defra, 2013)<br><br>The utilised agricultural area (UAA) in England is 9.0 million hectares (Defra, 2013)<br><br>Assume meeting necessary societal demands (no specific target) - quantity not limiting factor		UAA in England increased by 1% between 2012 and 2013 from 8.9 to 9.0 million hectares (Defra, 2013)<br><br>Production increased since 1945 driven by technology and policy (UK NEA, 2011)	**A**<br><br>(5)
			**A**		**B**<br>+ve	
			(2)		(3)	
Semi-Natural Grassland	Aesthetics	Spatial Configuration	Assumed target met based on Government ability to designate areas to meet societal preferences.		N/A	**A**<br><br>(8)
			(4)		(4)	
Woodland	Aesthetics	Quality	By area, 86% of SSSI woodland and wood-pasture is in favourable or recovering condition (SoNE, 2008)	≥95% SSSI favourable/ recovering by 2020 (Biodiversity Strategy 2020)	No significant change in species richness in broadleaved or coniferous woodland between 1998 and 2007 (Countryside Survey 2007)	**A**<br><br>(4)
			**A**<br>(-10% from target)		**B**<br>(stable)	
			(2)		(2)	
Semi-	Aesthetics	Quality	By area, 83% of SSSI	≥95% SSSI favourable/	Neutral grassland stable condition	**A**

natural Grassland			grassland (all types) is in favourable or recovering condition (SoNE, 2008)	recovering by 2020 (Biodiversity Strategy 2020)	and significant increase in area, calcareous grassland stable and under management to conserve, mixed improvements and declines in acid grassland (Countryside Survey 2007)	(5)
			**A** (-20% from target)		**A** (stable)	
			(2)		(3)	
Semi-natural Grassland	Equable climate	Quality	By area, 83% of SSSI grassland (all types) is in favourable or recovering condition (SoNE, 2008)	≥95% SSSI favourable/ recovering by 2020 (Biodiversity Strategy 2020)	Neutral grassland stable condition and significant increase in area, calcareous grassland stable and under management to conserve, mixed improvements and declines in acid grassland (Countryside Survey 2007)	**A** (5)
			**A** (-20% from target)		**A** (stable)	
			(2)		(3)	
Enclosed farmland	Equable climate	Quality	Greenhouse gas emissions from agriculture = ~7.0% of the UK total (UK NEA, 2011)<br><br>Agriculture was responsible for 43% of total UK methane emissions; 84% of total nitrous oxide emissions; and 86% of total ammonia emissions in 2012 (UK NEA, 2011)	No target	From 1990 emissions declined - nitrous oxide (-23%), methane (-18%) and carbon dioxide (-19%) (UK NEA, 2011)<br><br>2000- 2011 soil nutrient balances for nitrogen and phosphorus are estimated to have fallen (19% and 33% respectively) indicating a reduction in the nutrient surpluses which could be lost to the environment (Defra, 2012 - AUK)	**A** (6)
			**B** (unknown)		**A** (+ve +ve)	
			(4)		(2)	
Woodland	Clean air	Quantity	9% of England is wooded (UK NEA, 2011)	12% woodland cover by 2060 (DefraForestry and Woodlands Policy Statement, 2013)	Since 1945, the area of woodland has doubled to cover 12% of the UK (UK NEA, 2011)<br><br>Total area of the UK covered by	**A** (4)

					woodland increased by 0.3% 2010-2011 (ONS, 2012)	
			**A**<br>(-30% from target)		**A**<br>(+ve)	
			(2)		**(2)**	
Woodland	Clean air	Quantity	9% of England is wooded (UK NEA, 2011)	12% woodland cover by 2060 (DefraForestry and Woodlands Policy Statement, 2013)	Since 1945, the area of woodland has doubled to cover 12% of the UK (UK NEA, 2011)<br><br>Total area of the UK covered by woodland increased by 0.3% 2010-2011 (ONS, 2012)	**A**<br><br>(4)
			**A**<br>(-30% from target)		**A**<br>(+ve)	
			(2)		(2)	
Woodland	Recreation	Quantity	9% of England is wooded (UK NEA, 2011)	12% woodland cover by 2060 (DefraForestry and Woodlands Policy Statement, 2013)	Since 1945, the area of woodland has doubled to cover 12% of the UK (UK NEA, 2011)<br><br>Total area of the UK covered by woodland increased by 0.3% 2010-2011 (ONS, 2012)	**A**<br><br>(4)
			**A**<br>(-30% from target)<br>(2)		**A**<br>(+ve)<br>(2)	
			(2)		(2)	
Woodland	Aesthetics	Quantity	9% of England is wooded (UK NEA, 2011)	12% woodland cover by 2060 (Defra Forestry and Woodlands Policy Statement, 2013)	Since 1945, the area of woodland has doubled to cover 12% of the UK (UK NEA, 2011)<br><br>Total area of the UK covered by woodland increased by 0.3% 2010-2011 (ONS, 2012)	**A**<br><br>(4)
			**A**<br>(-30% from target)		**A**<br>(+ve)	
			(2)		(2)	
Woodland	Aesthetics	Spatial Configuration	9% of England is wooded (UK NEA, 2011)	12% woodland cover by 2060 (Defra Forestry and Woodlands Policy Statement, 2013)	Since 1945, the area of woodland has doubled to cover 12% of the UK (UK NEA, 2011)<br><br>Total area of the UK covered by	**A**<br><br>(4)

					woodland increased by 0.3% 2010-2011 (ONS, 2012)	
			**A**<br>(-30% from target)		**A**<br>(+ve)	
			(2)		(2)	
Woodland	Hazard protection	Quantity	9% of England is wooded (UK NEA, 2011)	12% woodland cover by 2060 (Defra Forestry and Woodlands Policy Statement, 2013)	Since 1945, the area of woodland has doubled to cover 12% of the UK (UK NEA, 2011)<br><br>Total area of the UK covered by woodland increased by 0.3% 2010-2011 (ONS, 2012)	**A**<br><br>(4)
			**A**<br>(-30% from target)		**A**<br>(+ve)	
			(2)		(2)	
Woodland	Wildlife	Quantity	9% of England is wooded (UK NEA, 2011)	12% woodland cover by 2060 (Defra Forestry and Woodlands Policy Statement, 2013)	Since 1945, the area of woodland has doubled to cover 12% of the UK (UK NEA, 2011)<br><br>Total area of the UK covered by woodland increased by 0.3% 2010-2011 (ONS, 2012)	**A**<br><br>(4)
			**A**<br>(-30% from target)		**A**<br>(+ve)	
			(2)		(2)	
Woodland	Equable climate	Quantity	9% of England is wooded (UK NEA, 2011)	12% woodland cover by 2060 (DefraForestry and Woodlands Policy Statement, 2013)	Since 1945, the area of woodland has doubled to cover 12% of the UK (UK NEA, 2011)<br><br>Total area of the UK covered by woodland increased by 0.3% 2010-2011 (ONS, 2012)	**A**<br><br>(4)
			**A**<br>(-30% from target)		**A**<br>(+ve)	
			(2)		(2)	
Urban	Aesthetics	Quality	Taking residents' satisfaction with local parks and green spaces as an indicator of condition, on average 73% of urban residents in England are 'satisfied' or 'highly	No formal or statutory target for the 'performance' of urban green space<br><br>Assume that everyone	Decline in quality of urban green space in England has been halted in most areas and there are signs of recovery in many places (NAO, 2006).	**A**<br><br>(6)

			satisfied' (NAO, 2006) (Eftec, asset check)	(100%) should be satisfied with greenspace.	In 2005, 16% of green space managers perceived the condition of urban green space in their local authority to be declining, 41% stable, and 43% improving (NAO, 2006) (Eftec, asset check)	
			**A**<br>(-30%)		**A**<br>(+ve)	
			(4)		(2)	
Coastal margin	Recreation	Quality	England's coastline is estimated to be over 7,000 km in length (SoNE 2008)<br><br>In 2008, 17 bathing waters failed to meet the mandatory standard. 16 of these were in England . (EA, Our Corporate Strategy 2010-2015 Evidence: water).	Right of access to all the coast of England has been created by the Marine and Coastal Access Act 2009 (UK NEA, 2011)<br><br>Bathing Water Directive	2013 - 55 beaches received the Blue Flag, this is down on the 79 for 2012 (Daily Mail, 2013)<br><br>Bathing water quality has improved over time. In 2008, 97% of bathing waters in England and Wales met water quality standards, compared to 78% in 1990 (EA, Our Corporate Strategy 2010-2015 Evidence: water)	**A**<br><br>(7)
			**A**<br>(+ve -ve)		**A**<br>(+ve -ve)	
			(4)		(3)	

Table 2: RAG status B

<table>
<tr><th>Habitat type</th><th>Benefit</th><th>Characteristic</th><th>Current Status</th><th>Target</th><th>Trend</th><th>RAG (A-C)</th></tr>
<tr><td rowspan="3">Mountains moors and heath</td><td rowspan="3">Aesthetics</td><td rowspan="3">Quality</td><td>SSSI in favourable status: blanket bog =58%, upland fen and marsh = 46% upland heath = 71% lowland heath = 81% (SoNE, 2008)</td><td>≥95% SSSI favourable/ recovering by 2020 (Biodiversity Strategy 2020)</td><td>Peatland bog areas decreased significantly last 60yrs, area of active peat bog declining by <1% per annum, 1990 to 1998 (UK NEA, 2011)</td><td rowspan="3">B

(8)</td></tr>
<tr><td colspan="2">B
(-40% from target)</td><td>B
-ve</td></tr>
<tr><td colspan="2">(4)</td><td>(4)</td></tr>
<tr><td rowspan="3">Mountains moors and heath</td><td rowspan="3">Hazard protection</td><td rowspan="3">Quantity</td><td>Coverage of upland areas = ~0.7million ha in England (UK NEA, 2011)</td><td>No target</td><td>No trend</td><td rowspan="3">B

(8)</td></tr>
<tr><td colspan="2">B
(unknown)</td><td>B
(unknown)</td></tr>
<tr><td colspan="2">(4)</td><td>(4)</td></tr>
<tr><td>Mountains moors and heath</td><td>Hazard protection</td><td>Quality

Soil erosion = f [ecological communities; soils (pH, nutrient concentrations (TOC, nitrate, phosphate, ammonium), erosion, infiltration), freshwater (water table) land (gradient), atmosphere (temperature, rainfall and wind);pressures (management practices e.g. low grazing, low drainage gripping, limit burning)]</td><td>SSSI in favourable status: blanket bog =58%, upland fen and marsh = 46% upland heath = 71% lowland heath = 81% (SoNE, 2008)</td><td>≥95% SSSI favourable/ recovering by 2020 (Biodiversity Strategy 2020)</td><td>Peatland bog areas decreased significantly last 60yrs, area of active peat bog declining by <1% per annum, 1990 to 1998 (UK NEA, 2011)

10-30% of UK peatland upland was subject to serious erosion (Eftec, asset check)</td><td>B

(8)</td></tr>
</table>

Habitat type	Benefit	Characteristic	Current Status	Target	Trend	RAG (A-C)
		*Flooding risk = f [**ecological communities; soils** (pH, nutrient concentrations (TOC, nitrate, phosphate, ammonium), erosion, infiltration), **freshwater** (water table) **land** (gradient), **atmosphere** (rainfall);**pressures** (management practices e.g. low drainage gripping, limit burning)]*<br><br>*Wildlife risk = f [**ecological communities** (heath biomass, blanket bog); **soils** (eroding); **freshwater** (low water table); **atmosphere** (temperature, rainfall); **pressures** (management practices e.g. burning regime)]*	**B**<br>(-40% from target)<br>(4)		**B**<br>(-ve)<br>(4)	
Mountains moors and heath	Wildlife	Quantity	Coverage of upland areas = ~0.7million ha in England (UK NEA, 2011)<br><br>UK has 75% of Europe's upland heath, and 10-15% of the world's blanket bog (Eftec, asset check)	No target	No trend.	**B**<br><br>(8)
			**B**<br>(unknown)		**B**<br>(unknown)	
			(4)		(4)	

Habitat type	Benefit	Characteristic	Current Status	Target	Trend	RAG (A-C)
Mountains moors and heath	Wildlife	Quality<br><br>*= f [* ***species;*** ***ecological communities*** *(pollination),* ***soils*** *(pH, nutrient concentrations (TOC, nitrate, phosphate, ammonium), erosion, infiltration),* ***freshwater*** *(water table);* ***land*** *(altitude, gradient, topography),* ***atmosphere*** *(temperature, rainfall, CO_2, N);* ***pressures*** *(management practices e.g. grazing, drainage gripping, burning)]*	SSSI in favourable status: blanket bog =58%, upland fen and marsh = 46% upland heath = 71% lowland heath = 81% (SoNE, 2008)<br><br>11% upland subject to drainage gripping (Eftec, asset check)	≥95% SSSI favourable/ recovering by 2020	Peatland bog areas decreased significantly last 60yrs, area of active peat bog declining by <1% per annum, 1990 to 1998 (UK NEA, 2011)<br><br>Vegetation richness stable 1998-2007 (Countryside Survey 2007)<br><br>Lowland heath birds recovering, upland wetland birds declining (SoNE, 2008)	**B**<br><br>(5)
			**B**<br>(-40% from target)		**B**<br>(-ve)	
			(2)		(3)	
Enclosed farmland	Food	Quality<br><br>*Crop yield = f [* ***species*** *(crop type);* ***soils*** *(agricultural Grade I – V, erosion);* ***land*** *(aspect, altitude, gradient, exposure to wind);* ***atmosphere*** *(temperature, rainfall),* ***freshwater*** *(groundwater);* ***minerals*** *(potassium, magnesium);* ***ecological communities*** *(pollination, invasive species/disease);* ***material capital*** *(management practices e.g. irrigation, pest/disease control, nutrient enrichment, aeration of soil, crop rotation, GM crops)]*	***SOILS:***<br>Grades 1 & 2 = 21% England; Subgrade 3a =21% (Natural England, 2012)<br><br>***ECOLOGICAL COMMUNITIES (POLLINATORS)***<br>Honeybees colonies in the UK = 274,000 (Eftec, asset check)	***SOILS:***<br>CAP (GAEC) maintain organic matter, reduce soil erosion risk & damage to soil structure.<br><br>By 2030, all England's soils will be managed sustainably and degradation threats tackled successfully (Defra, 2009 - Soil Strategy)<br><br>***ECOLOGICAL COMMUNITIES (POLLINATORS)***<br>Unknown	***SOILS***<br>Unknown<br><br>***ECOLOGICAL COMMUNITIES (POLLINATORS)***<br>1985-2005, honey bee colonies declined by 54% in England (UK NEA, 2011)<br><br>1990-2010, the honeybee species richness decline less dramatic than 1950-1989.<br>Solitary bee species have recovered, rates of wild flower species decline have slowed (Eftec, asset check)	**B**<br><br>(7)
			**B**<br>(unknown)		**B**<br>(-ve)	

Habitat type	Benefit	Characteristic	Current Status	Target	Trend	RAG (A-C)
			(4)		(3)	
Enclosed farmland	Clean water	Quantity	52.1% of land area in England is Enclosed farmland (Defra, 2013)<br><br>The utilised agricultural area (UAA) in England is 9.0 million hectares (Defra, 2013)	Unknown	Unknown	**B**<br><br>(8)
			(4)		(4)	
Enclosed farmland	Clean water	Quality<br><br>*Water quality = f [**species**; **ecological communities** (pollination, pollutant uptake);* **soils** *(exposure); **freshwater** (temperature, suspended sediment); **land** (aspect, altitude, gradient, exposure to wind); **atmosphere** (temperature, rainfall, wind); **material capital** (management practices e.g. use of buffer strips, reduced application of fertilisers, ploughing direction to reduce soil erosion, crop rotation to maximise uptake of nutrients for different plant species)]*	27% of England's freshwater bodies are currently classified as being of 'good status' or 'potential' or better (Environment Agency)<br><br>Agriculture was responsible for 28% of the damage to rivers due to phosphorous and 61% due to nitrogen in 2012 (Defra, 2013)	All inland and coastal waters within river basin districts must reach at least good status by 2015 (WFD)	Nitrate levels in English rivers have fallen overall since 2000 reflecting a decrease in fertiliser use (UK NEA, 2011)<br><br>2000- 2011 soil nutrient balances for nitrogen and phosphorus are estimated to have fallen (19% and 33% respectively) indicating a reduction in the nutrient surpluses which could be lost to the environment (Defra, 2012 - AUK)	**B**<br><br>(4)
			**C**<br>(-50% from target)		**A**<br>(+ve)	
			(2)		(2)	
Enclosed farmland	Hazard protection	Quality	Agriculture contributes to	***SOILS:***<br>CAP (GAEC) maintain	No trend information	**B**

Habitat type	Benefit	Characteristic	Current Status	Target	Trend	RAG (A-C)
		*Soil erosion = f [**species**; **ecological communities** (pollination); **soils** ; **land** (aspect, altitude, gradient, exposure to wind); **atmosphere** (temperature, rainfall, wind); **material capital** (management practices e.g. use of buffer strips, ploughing direction to reduce soil erosion, field drainage)]*	approximately 75% of sediment in watercourses<br><br>1/3 waterbodies are at risk from eroded soil (EA, Corporate Strategy 2010-2015)<br><br>***SOILS:***<br>Grades 1 & 2 = 21% England; Subgrade 3a =21% (Natural England, 2012)<br><br>2.2 million tonnes of topsoil are eroded each year (EA, Corporate Strategy 2010-2015).	organic matter, reduce soil erosion risk & damage to soil structure.<br><br>By 2030, all England's soils will be managed sustainably and degradation threats tackled successfully (Defra, 2009 - Soil Strategy)		(8)
			**B**<br>(unknown)<br>(4)		**B**<br>(unknown)<br>(4)	
			(4)		(4)	
Enclosed farmland	Wildlife	Quantity	60,000 ha of permanent grassland margins, 7,000 ha of cultivated margins, 9,000 ha of wild bird mix and 3,600 ha of flower margins for bumblebees and other insects (via AES) (SoNE, 2008)	No target	Landscape diversity improved through AES and set aside schemes - area of enclosed grassland increased by 5.4% between 1998 and 2007 (UK NEA, 2011)<br><br>Hedgerows in GB declined - ~624,000 km in 1984 to ~506,000km by 1990 (UK NEA, 2011)	**B**<br><br>(7)
			**B**<br>(unknown)		**A**<br>(+ve -ve)	

Habitat type	Benefit	Characteristic	Current Status	Target	Trend	RAG (A-C)
			(4)		(3)	
Enclosed farmland	Wildlife	Spatial Configuration	60,000 ha of permanent grassland margins, 7,000 ha of cultivated margins, 9,000 ha of wild bird mix and 3,600 ha of flower margins for bumblebees and other insects (via AES) (SoNE, 2008)	No target	Landscape diversity improved through AES and set aside schemes - area of enclosed grassland increased by 5.4% between 1998 and 2007 (UK NEA, 2011)<br><br>Hedgerows in GB declined - ~624,000 km in 1984 to ~506,000km by 1990 (UK NEA, 2011)	**B**<br><br>(7)
			**B**<br>(unknown)		**A**<br>(+ve -ve)	
			(4)		(3)	
Semi natural grassland	Wildlife	Quantity	Semi-natural grassland equates to approximately 109,576 ha covering 1% of the total area of England (SoNE, 2008)	No target	Countryside Survey 2007 - no change in area of acid and calcareous grasslands in each of the UK countries . There was a significant increase in the area of neutral grassland (UK NEA, 2011)	**B**<br><br>(6)
			**B**<br>(unknown)		**A**<br>(stable/+ve)	
			(4)		(2)	
Woodland	Fibre	Quality<br><br>*Fibre = f [species (hardwood and softwood), ecological communities (invasive, pests and disease); **soils** (decomposers, nitrifying bacteria - nitrogen fixation, nutrient cycling); **freshwaters** (groundwater); **land** (altitude, gradient); **atmosphere** (rain, temperature, nitrogen, carbon dioxide, wind); **minerals***	Total of 8.4 million green tonnes of softwood was produced in the UK in 2008, hardwood production of 0.4 million green tonnes (UK NEA, 2011)<br><br>***ECOLOGICAL COMMUNITIES:***	No target	***ECOLOGICAL COMMUNITIES:***<br>Statutory Plant Health Notices served 2012-2013 - 89% increase from 2011-12 (Forestry Commission, 2013)	**B**<br><br>(8)

Habitat type	Benefit	Characteristic	Current Status	Target	Trend	RAG (A-C)
		(potassium, magnesium); ***material capital*** *(management - coppicing, felling, crop rotation, irrigation, processing timber, machinery transport, pest control, nutrient enrichment, pollution -* SO_2*)]*	Statutory Plant Health Notices (prevent spread of pests and diseases) - 418 notices were served in England between 2010 and 2013 (= ~2.1 thousand hectares) (Forestry Commission, 2013)			
			**B** (unknown)		**B** (-ve)	
			(4)		(4)	
Woodland	Clean water	Quantity	9% of England is wooded (UK NEA, 2011)	Unclear relationship	Unknown trend	**B** (8)
			**B** (unknown)		**B** (unknown)	
			(4)		(4)	
Woodland	Clean water	Spatial Configuration	A study demonstrated that 99% of nitrate draining from arable fields in southern England during winter was retained within the first 5m of a buffer planted with poplar (The Woodland Trust, 2008).	No target	Unknown trend	**B** (8)
			**B** (unknown)		**B** (unknown)	

**Habitat type**	**Benefit**	**Characteristic**	**Current Status**	**Target**	**Trend**	**RAG (A-C)**
			(4)		(4)	
Woodland	Recreation	Spatial Configuration	55% of the population have access to woods greater than 20 ha within 4 km, and 10% have access to woods greater than 2 ha within 500 m of their home (Woodland Trust 2004)	Woodland Access Standard:<br>• no person live >500m from at least one area of accessible woodland (2ha)<br>• at least one area of accessible woodland 20ha within 4km (8km roundtrip) (Woodland Trust)	Overall, the percentage of people in the UK with access to woodland has increased over the five-year period from 2004 to 2009 (The Woodland Trust, 2010).	**B**<br><br>(6)
			**B**<br>(-40% from target)		**A**<br>(+ve)	
			(4)		(2)	
Woodland	Hazard protection	Spatial Configuration	9% of England is wooded (UK NEA, 2011)	No target for spatial configuration and flooding	Unknown trend	**B**<br><br>(8)
			**B**<br>(unknown)		**B**<br>(unknown)	
			(4)		(4)	
Woodland	Wildlife	Quality<br><br>*Wildlife = f [**species**; **ecological communities** (invasives, pests and disease); **soils** (decomposers, nitrifying bacteria - nitrogen fixation, nutrient cycling); **freshwaters** (groundwater); **land** (altitude, gradient); **atmosphere** (rain, temperature, nitrogen, carbon dioxide, wind); **minerals** (potassium, magnesium); **material***	86% SSSI favourable/ Recovering (SoNE, 2008)<br><br>~10% vascular woodland plants threatened (SoNE, 2008).<br><br>No recovery from bird declines in 1990's, ~20% (SoNE, 2008)	≥95% SSSI favourable/ recovering by 2020 (Biodiversity Strategy 2020)	<1980s much conversion to plantations (UK NEA, 2011)<br><br>No significant change in species richness in broadleaved or coniferous woodland between 1998 and 2007 (Countryside Survey 2007)<br><br>Increase in UK BAP habitats (SoNE, 2008).	**B**<br><br>(7)

Habitat type	Benefit	Characteristic	Current Status	Target	Trend	RAG (A-C)
		capital *(management -coppicing, felling, restocking with native species, dead log piles, pest control, pollution - SO_2)]*			Woodland Bird Survey – mixed (1980s-2003/4) and major decline in butterflies (SoNE, 2008)	
			**A** (-10%)		**B** (-ve)	
			(4)		(3)	
Woodland	Wildlife	Spatial Configuration	Low connectivity across landscape (UK NEA, 2011). Our woodland resource is highly fragmented (Biodiversity Strategy 2020)	No target for connectivity	Little or no overall change in the degree of connectivity for broad-leaved, mixed and yew woodland between 1990 and 2007. Over the same period there has been an increase in the area of broad-leaved woodland, which would tend to increase connectivity (JNCC Biodiversity Indicators, 2013)<br><br>Increase in UK BAP habitats (SoNE, 2008).	**B**<br><br>(6)
			**B** (unknown)		**A** (stable/+ve)	
			(4)		(2)	
Freshwater	Clean water	Quantity	The UK has at least 392,000 ha of fens, reedbed, lowland raised bog and grazing marsh (UK NEA, 2011)	No target	~90% of the national resource of wetlands has been lost since Roman times, 13% of the floodplain resource degraded or completely disconnected from river channels and area of lowland raised bog retaining a largely undisturbed surface has declined by 94% (UK NEA, 2011)	**B**<br><br>(6)

Habitat type	Benefit	Characteristic	Current Status	Target	Trend	RAG (A-C)
			**B**<br>(unknown)		**B**<br>-ve	
			(4)		(2)	
Freshwater	Clean water	Quality<br><br>*Potable water = f [**freshwater** (water - volume, nutrient concentrations e.g. phosphorous, nitrate, dissolved organic carbon, bacteria levels (E.coli and streptococci), levels of cyanobacteria, phytoplankton, macro-algae), suspended sediment); **atmosphere** (temperature, rainfall); **material capital** (abstraction infrastructure, water treatment plants); **pressures** (pollution, abstraction rates)]*	27% of England's freshwater bodies are currently classified as being of 'good status' or 'potential' or better (Environment Agency)<br><br>99.96% of all tests in 2012 met drinking water standards (UK DWI, 2012)<br><br>South east and eastern England - 'under stress' due to water abstraction (EEA, 2003)	All inland and coastal waters within defined river basin districts must reach at least good status by 2015 (WFD)<br><br>Drinking Water Directive 1998 standards	Biological and chemical classification of 7,000 km of English rivers improved significantly from 1990 to 2008 (UK NEA, 2011)<br><br>Nitrate levels in English rivers have fallen overall since 2000 reflectinga decrease in fertiliser use in Enclosed farmland (UK NEA, 2011)<br><br>1.6% of drinking water standard failures in 1991 compared to 0.04% in 2012 (UK DWI, 2012)	**D**<br><br>(6)
			**C**<br>(-50% from target)		**A**<br>(+ve)	
			(2)		(2)	
Freshwater	Recreation	Quality<br><br>*Recreation = f [**freshwater** (water - volume, flow velocity, nutrients, bacteria, aquatic vegetation), **land** (gradient, altitude), **species** (fish), **material capital** (access, signage/waymarks), **pressures** (Enclosed farmland outputs)]*	27% of England's freshwater bodies are currently classified as being of 'good status' or 'good ecological potential' or better (Environment Agency)	All inland and coastal waters within defined river basin districts must reach at least good status by 2015 (WFD)	Biological and chemical classification of 7,000 km of English rivers improved significantly from 1990 to 2008 (UK NEA, 2011)<br><br>Nitrate levels in English rivers have fallen overall since 2000 reflecting a decrease in fertiliser use in Enclosed farmland (UK	**B**<br><br>(4)

Habitat type	Benefit	Characteristic	Current Status	Target	Trend	RAG (A-C)
					NEA, 2011)<br><br>Bathing water quality has improved over time - 97% met standards in 2008 compared to 78% in 1990 (EA, Our Corporate Strategy 2010-2015 Evidence: water)	
			**C**<br>(-50% from target)		**A**<br>(stable/+ve)	
			(2)		(2)	
Freshwater	Aesthetic	Quality<br><br>*Aesthetics = f [**freshwater** (water - volume, flow, nutrients, floodplain connectivity), **land** (gradient, altitude); **atmosphere** (temperature, rainfall); **species**, **material capital** (pollution e.g. oil, litter, absence of significant modifications)]*	27% of England's freshwater bodies are currently classified as being of 'good status' or 'good ecological potential' or better (Environment Agency)<br><br>55% open water, 69% wetland, 81% lowland raised bog, 87% fen, marsh and swamp SSSIs favourable/ recovering (SoNE, 2008)	All inland and coastal waters within defined river basin districts must reach at least good status by 2015 (WFD)<br><br>≥95% SSSI favourable/ recovering by 2020 (Biodiversity Strategy 2020)	Biological and chemical classification of 7,000 km of English rivers improved significantly from 1990 to 2008 (UK NEA, 2011)<br><br>Nitrate levels in English rivers have fallen overall since 2000 reflecting a decrease in fertiliser use in Enclosed farmland (UK NEA, 2011)	**B**<br><br>(4)
			**C**<br>(-50% from target)		**A**<br>(stable/+ve)	
			(2)		(2)	

Habitat type	Benefit	Characteristic	Current Status	Target	Trend	RAG (A-C)
Freshwater	Hazard protection	Quality<br><br>*Flood protection = f [**freshwater** (floodplain connectivity, extent and permeability, water - volume, flow velocity, suspended sediment), **land** (gradient), **atmosphere** (rainfall), **species** (woody debris), **material capital** (flow regulation, storage reservoirs, channel modification), **pressures** (Enclosed farmland outputs)]*	By area, 69% of wetland, 81% of lowland raised bogs, 87% of fen, marsh and swamp and 89% of lowland neutral grasslands SSSIs are in favourable or recovering condition (UK NEA, 2011)<br><br>More than 50% of English and Welsh rivers have been modified physically	≥95% SSSI favourable/ recovering by 2020 (Biodiversity Strategy 2020)	No trend information	**B**<br><br>(6)
			**A**<br>(-30% from target)		**B**<br>(unknown)	
			(2)		(4)	
Freshwater	Hazard protection	Spatial Configuration	Over 2/5 (42% by area) of all floodplains in England and Wales (defined by the 100-year flood envelope) have been separated from their rivers by flood embankments and channel modifications (UK NEA, 2011).	No target	Unknown trend	**B**<br><br>(8)
			**B**<br>(unknown)		**B**<br>(unknown)	
			(4)		(4)	

Habitat type	Benefit	Characteristic	Current Status	Target	Trend	RAG (A-C)
Freshwater	Wildlife	Quantity	There are more than 389,000 km of rivers in the UK, almost 6,000 permanent large lakes covering around 200,000 ha and nearly half a million ponds (covering less than 2 ha) (UK NEA, 2011)	No target<br><br>~ 1.1% (UK wide) of land for wetlands (lowland) is needed to deliver sustainable populations of all birds considered within a calculation by the RSPB (Pers. comm. Jo Gilbert 2007) (Hume, 2008 - Wetland Vision Technical Document)	~90% of the national resource of wetlands has been lost since Roman times, 13% of the floodplain resource degraded or completely disconnected from river channels and area of lowland raised bog retaining a largely undisturbed surface has declined by 94% (UK NEA, 2011)	**B**<br><br>(6)
			**B**<br>(unknown)		**B**<br>(-ve)	
			(4)		(2)	
Freshwater	Equable climate	Quantity	~ 392,000 ha of fens, reedbed, lowland raised bog and grazing marsh (UK NEA, 2011)<br><br>Remaining lowland fen in English peatlands stored 1,004–2,576 tonnes of carbon/ha, and raised bog peats stored 1,575–1,629 tonnes of carbon/ha (UK NEA, 2011)	No target	~90% of the national resource of wetlands has been lost since Roman times and area of lowland raised bog retaining a largely undisturbed surface has declined by 94% (UK NEA, 2011)<br><br>Lowland meadows have declined from 6,600,000 ha to 200,000 ha, fens 310,000 ha to 26,000 ha, reedbeds 10,000 ha to 6–8,000 ha (UK NEA, 2011)	**B**<br><br>(6)
			**B**<br>(unknown)		**B**<br>(-ve)	
			(4)		(2)	

Habitat type	Benefit	Characteristic	Current Status	Target	Trend	RAG (A-C)
Freshwater	Equable climate	Quality<br><br>*Carbon sequestration = f [**species** (plankton biomass); **freshwater** (water - volume, flow, nutrients, floodplain connectivity, suspended sediment, nutrient levels, acidity, groundwater); **land** (gradient, altitude); **atmosphere** (temperature); **pressures** (pollution e.g. oil, flow regulation, channel modification)]*	69% wetland, 81% lowland raised bog, 87% fen, marsh and swamp SSSIs favourable/ recovering (UK NEA, 2011)	≥95% SSSI favourable/ recovering by 2020 (Biodiversity Strategy 2020)	~90% of the national resource of wetlands has been lost since Roman times and area of lowland raised bog retaining a largely undisturbed surface has declined by 94% (UK NEA, 2011)<br><br>Lowland meadows have declined from 6,600,000 ha to 200,000 ha, fens 310,000 ha to 26,000 ha, reedbeds 10,000 ha to 6–8,000 ha (UK NEA, 2011)	**B**<br><br>(4)
			**A**<br>(-30% from target)		**B**<br>(-ve)	
			(2)		(2)	

Habitat type	Benefit	Characteristic	Current Status	Target	Trend	RAG (A-C)
Urban	Clean air	Quantity	~10% of England's land area is classified as urban (UK NEA, 2011)<br><br>90 % of city dwellers in the EU are exposed to damaging air pollutants (EEA AQ Report, 2013)	Air Quality Strategy 2007	Urban land cover projected to rise from 10.6% in 1991 to 11.9% in 2016 (SoNE, 2008)<br><br>Average projection for urban growth between 2011 and 2060 across UK is 3% (UKNEA. 2011)	**B**<br><br>(6)
			**B**<br>(-40% from target - approximate)		**B**<br>(-ve)	
			(4)		(2)	
Urban	Clean air	Quality<br><br>*Clean air = f [**species** (London plane trees); **atmosphere**; **material capital** (policies to cap emissions -* PM_{10}*,* NO_2*,* SO_2*, reduction in car useage, proportion of green space to built urban)]*	Current 7% tree cover in the West Midlands reduces air concentrations of PM10 (particulates < 10 micro-metres) by 4% (McDonald *et al*. 2007)	No target	Decline in condition of greenspace has been halted (NAO, 2004)<br><br>2004/2005 - 70% of urban street trees in good condition. Decline since 1992/3 data (UK NEA, 2011)	**B**<br><br>(6)

**Habitat type**	**Benefit**	**Characteristic**	**Current Status**	**Target**	**Trend**	**RAG (A-C)**
			**B**<br>(unknown)		**B**<br>(-ve)	
			(4)		(2)	
Urban	Recreation	Quantity	Total extent of urban green space in GB is ~290,000 ha (Eftec, asset check)	No target	Urban land cover projected to rise from 10.6% in 1991 to 11.9% in 2016 (SoNE, 2008)<br><br>Average projection for urban growth between 2011 and 2060 across UK is 3% (UKNEA. 2011)	**B**<br><br>(8)
			**B**<br>(unknown)		**B**<br>(-ve)	
			(4)		(4)	
Urban	Recreation	Spatial Configuration	Mean accessible greenspace is 2 hectares (ha) per 1,000 people in England (UK NEA, 2011)<br><br>Wards with fewer than 20 dwellings per hectare have three times as much greenspace as wards in high density areas (Eftec, asset check)	Accessible Natural Greenspace Standards (ANGSt) - 1ha of LNR per 1000 pop, 20ha site within 2km from home (UKNEAFO, 2014)	Urban land cover projected to rise from 10.6% in 1991 to 11.9% in 2016 (SoNE, 2008)<br><br>Average projection for urban growth between 2011 and 2060 across UK is 3% (UKNEA. 2011)	**B**<br><br>(8)
			**B**<br>(-40% from target - approximate)		B<br>(-ve)	

Habitat type	Benefit	Characteristic	Current Status	Target	Trend	RAG (A-C)
			(4)		(4)	
Urban	Aesthetics	Quantity	Total extent of urban green space in GB is ~290,000 ha (Eftec, asset check)<br><br>Mean accessible greenspace is 2 hectares (ha) per 1,000 people in England (UK NEA, 2011)<br><br>Wards with fewer than 20 dwellings per hectare have three times as much greenspace as wards in high density areas (Eftec, asset check)	No target	Urban land cover projected to rise from 10.6% in 1991 to 11.9% in 2016 (SoNE, 2008)<br><br>Average projection for urban growth between 2011 and 2060 across UK is 3% (UKNEA. 2011)	**B**<br><br>(8)
			**B**<br>(unknown)		**B**<br>(-ve)	
			(4)		(4)	
Urban	Aesthetics	Spatial Configuration	Use of Natural England's Accessible Natural Greenspace Standards (ANGSt) (?)	No formal or statutory target for the ‘performance’ of urban green space in the UK	No trend information	**B**<br><br>(8)
			**B**<br>(unknown)		**B**<br>(unknown)	
			(4)		(4)	

Habitat type	Benefit	Characteristic	Current Status	Target	Trend	RAG (A-C)
Urban	Hazard protection	Quantity	80,000 homes in England and Wales at risk of urban flooding	No target	Urban land cover projected to rise from 10.6% in 1991 to 11.9% in 2016 (SoNE, 2008)<br><br>Average projection for urban growth between 2011 and 2060 across UK is 3% (UKNEA. 2011)	**B**<br><br>(6)
			**B**<br>(unknown)		**B**<br>(-ve)	
			(4)		(2)	
Urban	Wildlife	Quantity	The total extent of urban green space in GB is just under 290,000 ha (Eftec, asset check)	No target	Urban land cover projected to rise from 10.6% in 1991 to 11.9% in 2016 (SoNE, 2011)<br><br>Average projection for urban growth between 2011 and 2060 across UK is 3% (UKNEA. 2011)	**B**<br><br>(6)
			**B**<br>(unknown)		**B**<br>(-ve)	
			(4)		(2)	
Urban	Wildlife	Quality	Common frog, song thrush and hedgehog, are found in significant numbers in urban areas and particularly domestic gardens (SoNE 2008)	No target	Increase in urban generalist bird species between 1994 and 2006 e.g. woodpecker, wood pigeon, goldfinch. Urban specialists e.g. swift, house martin, collared dove and house sparrow have declined by 15% over this period (SoNE 2008).	**B**<br><br>(8)

Habitat type	Benefit	Characteristic	Current Status	Target	Trend	RAG (A-C)
			'Wider countryside' butterfly species more likely to be found in suburban areas than in rural areas (SoNE 2008).			
			**B** (unknown)		**A** (+ve -ve)	
			(4)		(4)	
Urban	Wildlife	Spatial configuration	Green corridors are generally poorly quantified by local authorities making their extent and condition difficult to assess	No target	Green corridors included in planning and conservation policy in 2010 through their inclusion in the UK BAP as Open Mosaic Habitats.(UK NEA)<br><br>No trend information.	**B**<br><br>(8)
			**B** (unknown)		**B** (unknown)	
			(4)		(4)	
Urban	Equable climate	Quantity	The total extent of urban green space in GB is just under 290,000 ha (Eftec, asset check)	No target	Urban land cover projected to rise from 10.6% in 1991 to 11.9% in 2016 (SoNE, 2011)<br><br>Average projection for urban growth between 2011 and 2060 across UK is 3% (UKNEA. 2011)	**B**<br><br>(6)
			**B** (unknown)		**B** (-ve)	
			(4)		(2)	

Habitat type	Benefit	Characteristic	Current Status	Target	Trend	RAG (A-C)
Coastal margins	Aesthetics	Quality<br><br>*Aesthetics = f [**coasts** (abundance of habitats); **oceans** (view, sense of being at seaside); **ecological communities** (wildlife associated with habitats); **material capital** (hard engineering, cultural memories, archaeology and heritage)]*	By area, 91% of SSSI coastal habitat is in favourable or recovering condition (SoNE, 2008)	≥95% SSSI favourable/ recovering by 2020 (Biodiversity Strategy 2020)	Proportion of early successional habitats has decreased -by up to 90% in some dune systems—while scrub and grassland have increased (UK NEA, 2011)<br><br>Quality of coastal margin habitats has declined since 1945 due to changes in soft sediment supply (UK NEA, 2011)	**B**<br><br>(4)
			**A**<br>(-10% from target)		**B**<br>(-ve)	
			(2)		(2)	
Coastal margins	Aesthetics	Spatial Configuration	Unknown		Unknown	**B**<br><br>(8)
			(4)		(4)	
Coastal margins	Hazard protection	Quantity	Approximately 44% of the English coastline is dEnclosed farmlandended, with 30% of the coastline eroding (Defra)	No target	Coastal margin habitats have declined by an estimated 16% since 1945 due to development and coastal squeeze (UK NEA, 2011)<br><br>Sand dunes - 30% loss since 1900. Major saltmarsh loss pre-1980s, current losses are estimated at 100ha per year (UK NEA, 2011).	**B**<br><br>(6)
			**B**<br>(unknown)		**B**<br>(-ve)	
			(4)		(2)	
Coastal margins	Hazard protection	Quality	By area, 91% of SSSI coastal habitat	≥95% SSSI favourable/ recovering by 2020	Proportion of early successional habitats has	**B**

Habitat type	Benefit	Characteristic	Current Status	Target	Trend	RAG (A-C)
		*Hazard protection = f [**species**; **coasts** (sediment, feature is wide and elevated, low creek density (saltmarsh)); **ecological communities** (colonisers such as Salicornia, sand dune stabilisers e.g. marram grass, tall and dense vegetation); **freshwater** (sediment); **land** (coastal morphology, aspect); **ocean** (tidal submergence, tidal current velocity, salinity, temperature)]+ [**pressures** (hard engineering structures - interrupt sediment flows, change wave action)]*	is in favourable or recovering condition (SoNE, 2008)	(Biodiversity Strategy, 2020)	decreased -by up to 90% in some dune systems—while scrub and grassland have increased (UK NEA, 2011)<br><br>Quality of coastal margin habitats has declined since 1945 due to changes in soft sediment supply (UK NEA, 2011)	(4)
			**A**<br>(-10% from target)		**B**<br>(-ve)	
			(2)		(2)	
Coastal margins	Wildlife	Quantity	Sand dunes, machair, saltmarsh, shingle, sea cliffs and coastal lagoons) make up only 0.6% of the UK's land area. Sand dunes and saltmarsh have areas of approximately 70,000 hectare (ha) and 45,000 ha respectively (UK NEA, 2011)	No target	Coastal margin habitats have declined by an estimated 16% since 19452 due to development and coastal squeeze (UK NEA, 2011)<br><br>Sand dunes - 30% loss since 1900. Major saltmarsh loss pre-1980s, current losses are estimated at 100ha per year (UK NEA, 2011)	**B**<br><br>(6)
			**B**<br>(unknown)		**B**<br>(-ve)	
			(4)		(2)	
Coastal margins	Wildlife	Quality<br><br>*Wildlife = f [**species** (specialised, native, range of successional species); **ecological communities** (mosaic of habitats, range of*	By area, 91% of SSSI coastal habitat is in favourable or recovering condition (SoNE, 2008)	≥95% SSSI favourable/ recovering by 2020 (Biodiversity Strategy 2020)	Proportion of early successional habitats decreased -by up to 90% in some dune systems—scrub and grassland have increased (UK NEA, 2011)	**B**<br><br>(4)

Habitat type	Benefit	Characteristic	Current Status	Target	Trend	RAG (A-C)
		successional stages, maintenance of stable systems); ***freshwater*** *(sediment);* ***land*** *(coastal morphology incl. aspect and gradient);* ***atmosphere*** *(wind);* ***oceans*** *(tidal submergence, water velocity, turbulence, salinity levels, nutrient levels);* ***coasts*** *(stable systems, sediment, soil pH);* ***material capital*** *(management regimes e.g. light grazing, scrub clearance, lack of disturbance on shingle);* ***pressures*** *(air pollution - acidification from sulphur and nitrogen deposition)]*	***SPECIES:***<br>England's mudflats support some 4.3-4.7 million such birds in winter (~70% to 80% of GB total) (SoNE, 2008)		Sand dunes - 30% loss since 1900. Major saltmarsh loss pre-1980s, current losses are estimated at 100ha per year (UK NEA, 2011).<br><br>***SPECIES:***<br>Average numbers of waterbirds wintering in, or migrating through, marine areas in the UK doubled mid-1970s - mid-1990s. However, some species of diving duck and estuarine wader have recently declined (SoNE, 2008)<br><br>***COASTS:***<br>Quality of coastal margin habitats has declined since 1945 due to changes in soft sediment supply (SoNE, 2008)	
			**A**<br>(-10% from target)		**B**<br>(-ve)	
			(2)		(2)	
Coastal margins	Equable climate	Quantity	Sand dunes, machair, saltmarsh, shingle, sea cliffs and coastal lagoons) make up only 0.6% of the UK's land area. Sand dunes and saltmarsh have areas of approximately 70,000 hectare (ha) and 45,000 ha	No target	Coastal margin habitats have declined by an estimated 16% since 19452 due to development and coastal squeeze (UK NEA, 2011)<br><br>Sand dunes - 30% loss since 1900. Major saltmarsh loss pre-1980s, current losses are estimated at 100ha per year (UK	**B**<br><br>(6)

Habitat type	Benefit	Characteristic	Current Status	Target	Trend	RAG (A-C)
			respectively (UK NEA, 2011)<br><br>Sand dunes on the west coast of the UK store 0.58 to 0.73t C/ha/yr, while saltmarsh stores 0.64 to 2.19 t C/ha/yr (UK NEA, 2011)		NEA, 2011).	
			**B**<br>(unknown)<br>(4)		**B**<br>(-ve)<br>(2)	
			(4)		(2)	
Coastal margins	Equable climate	Quality<br><br>*Carbon sequestration=f [**coasts** (sediment); **atmosphere** (wetter conditions); **ecological communities** (successional species, vegetation fixes CO_2)]*	By area, 91% of SSSI coastal habitat is in favourable or recovering condition (SoNE, 2008)	≥95% SSSI favourable/ recovering by 2020 (Biodiversity Strategy 2020)	Proportion of early successional habitats has decreased -by up to 90% in some dune systems—while scrub and grassland have increased (UK NEA, 2011)<br><br>Sand dunes - 30% loss since 1900. Major saltmarsh loss pre-1980s, current losses are estimated at 100ha per year (UK NEA, 2011).	**B**<br><br>(4)
			**A**<br>(-10% from target)		**B**<br>(-ve)	
			(2)		(2)	
Marine	Food	Quality<br><br>*Food = f [**species** (fish, shellfish); **coasts** (saltmarsh - nursery ground for fish species;, **atmosphere** (wind); **oceans** (salinity, currents,*	***SPECIES:***<br>50% of 18 indicator finfish stocks in UK waters = full reproductive capacity &harvested sustainably, 2008	***SPECIES:***<br>Fish stocks c. 1938-1970<br><br>***COASTS:***<br>≥95% SSSI favourable/ recovering by 2020 (Biodiversity Strategy 2020)	***SPECIES:***<br>10% of 18 indicator finfish stocks in UK waters = full reproductive capacity &harvested sustainably, 1998 (UK NEA, 2011)	**B**<br><br>(6)

Habitat type	Benefit	Characteristic	Current Status	Target	Trend	RAG (A-C)
		*tides, waves, temperature, pH); **ecological communities** (population regulation, food web dynamics); **land** (morphology); **pressures** (harvesting effort, harvesting preferences - policy driven, equipment, pollution)]*	(UK NEA, 2011)<br><br>The majority of stocks continue to be fished at rates well above the values expected to provide the highest long-term yield (UK NEA, 2011)<br><br>***COASTS:***<br>Intertidal mudflats and saltmarsh - 90% of SSSI area in favourable or recovering condition (SoNE, 2008)		***COASTS:***<br>Major saltmarsh loss pre-1980s, current losses are estimated at 100ha per year (UK NEA, 2011).	
			**B**<br>(-ve)		**B**<br>(-ve)	
			(4)		(2)	
Marine	Wildlife	Quality<br><br>*Wildlife = f [**species**; **ecological communities**; **land** (topography, elevation); **atmosphere** (wind), **oceans** (salinity, tides, currents, waves, temperature, pH); **material capital** (pollution (e.g. oil spills, sewage Enclosed farmlandfluent), invasive species (e.g. ballast water), fish by-catch)]*	Two of the four Annex I marine habitats for which SACs have been designated are in unfavourable condition (SoNE, 2008).<br><br>22% of UK large shallow inlets and bays identified as being 'at risk' of failing to meet the standard of GES (SoNE, 2008)	Marine Strategy Framework Directive (2008) - achieve Good Environmental Status (GES) in all UK marine waters by 2020	Most estuarine and marine fish communities have improved in recent years, however certain vulnerable fish have continued to deteriorate e.g. many deep-water fish species, and species that move between fresh- and saltwater, such as the European eel and sturgeon (UK NEA, 2011)<br><br>Between 2000 and 2008, the total number of breeding seabirds decreased by around 9% (UK NEA, 2011)	**B**<br><br>(7)

<table>
<tr><th>Habitat type</th><th>Benefit</th><th>Characteristic</th><th>Current Status</th><th>Target</th><th>Trend</th><th>RAG
(A-C)</th></tr>
<tr><td></td><td></td><td></td><td colspan="2">B
(-ve)
(4)</td><td>A
(+ve -ve)
(3)</td><td rowspan="2"></td></tr>
<tr><td></td><td></td><td></td><td colspan="2">(4)</td><td>(3)</td></tr>
</table>

Table 3: RAG status C

Habitat type	Benefit	Characteristic	Current Status	Target	Trend	RAG (A-E)
Mountains moors and heath	Clean water	Quality<br>*Clean water = f [**species** (sphagnum moss), **ecological communities** (vegetation - nutrient cycling, pollutant absorption), **soils** (pH, nutrient concentrations (TOC, nitrate, phosphate, ammonium), erosion, infiltration), **freshwater** (high water table) **land** (altitude, gradient), **atmosphere** (temperature and rainfall); pressures (management practices e.g. low intensity grazing, low drainage gripping, limit burning)]*	SSSI in favourable status: blanket bog =58%, upland fen and marsh = 46% upland heath = 71% lowland heath = 81% (SoNE, 2008)	≥95% SSSI favourable/ recovering by 2020 (Biodiversity Strategy 2020)	Peatland bog areas decreased significantly last 60yrs, area of active peat bog declining by <1% per annum, 1990 to 1998 (UK NEA, 2011)<br><br>10-30% of UK peatland upland was subject to serious erosion (Eftec, asset check)	**C**<br><br>(4)
			**B**<br>(-40% from target)		**C**<br>-ve -ve	
			(2)		(2)	
Mountains moors and heath	Equable climate	Quality<br><br>*Carbon sequestration = f [**species** (sphagnum moss); **ecological communities** (photosynthesis and carbon locking); **soils** (high acidity, organic matter and water holding capacity, nutrient availability); **atmosphere** (temperatures, rainfall, CO_2, N); **freshwater** (high water table); **land** (low gradient); **pressures** (extraction methods, land management - burning and grazing regimes)]*	40% of UK's soil carbon is stored in upland peatland ~300MtCO_2 (Eftec, asset check)<br><br>SSSI in favourable status: blanket bog =58%, upland fen and marsh = 46% upland heath = 71% lowland heath = 81% (SoNE, 2008)	≥95% SSSI favourable/ recovering by 2020 (Biodiversity Strategy 2020)<br><br>Restore 10,000km^2 peatland by 2020 (UK Committee Peatlands Programme, 2012)	Peatland bog areas decreased significantly last 60yrs, area of active peat bog declining by <1% per annum, 1990 to 1998 (UK NEA, 2011)<br><br>10-30% of UK peatland upland was subject to serious erosion (Eftec, asset check)<br><br>Peatlands are net-emitters of carbon due to degradation (Eftec, asset check).	**C**<br><br>(6)
			**B**<br>(-40% from target)		**C**<br>-ve -ve	
			(2)		(2)	

Enclosed farmland	Wildlife	Quality<br><br>*Wildlife = f [**species**; **ecological communities** (pollination); **soils**; **land**; **atmosphere**; **material capital** (management practices e.g. use of buffer strips, set aside schemes, creation of waterbodies, reduction in pesticide application, reduction in monoculture)]*	Only 26 out of 710 Areas/Sites of Special Scientific Interest on Enclosed farmland are in favourable condition (UK NEA, 2011)	≥95% SSSI favourable/ recovering by 2020 (Biodiversity Strategy 2020)	Increase in agri-environment scheme and payments under CAP.<br><br>Numbers of specialist farmland birds had fallen to 40% of their 1970 levels in 2000, and they have fallen a further 4% since then (UK NEA, 2011)<br><br>Specialist species (those restricted to semi-natural habitats) showing recovery, generalists stable trend (SoNE, 2008)	**C**<br><br>(5)
			**C**<br>(-50% from target)		**A**<br>(+ve -ve)	
			(2)		(3)	
Semi natural grassland	Wildlife	Quality<br><br>*Wildlife = f [**species** (high diversity); **ecological communities** (pollination); **soils**; **land** (topography); **atmosphere** (rain, temperature); **material capital** (conservation management - grazing, cutting, scrub management)]*	By area, 83% of SSSI grassland (all types) is in favourable or recovering condition (SoNE, 2008)<br><br>Calcareous grasslands provide breeding habitat for 85% of British butterfly species (SoNE, 2008)	≥95% SSSI favourable/ recovering by 2020 (Biodiversity Strategy 2020)	Neutral grassland stable condition and significant increase in area, calcareous grassland stable and under management to conserve, mixed improvements and declines in acid grassland (Countryside Survey 2007)<br><br>Significant decline in plant species richness 1998-2007 (Countryside Survey 2007)<br><br>Major declines in breeding and wintering birds associated with SNG, and butterflies (UK NEA, 2011)	**C**<br><br>(5)
			**A**<br>(-20% from target)		**C**<br>(-ve -ve)	
			(2)		(3)	

Freshwater	Wildlife	Quality<br><br>*Wildlife = f [**species; freshwater** (water - volume, flow, nutrients, floodplain connectivity, suspended sediment, nutrient levels, acidity, groundwater), **land** (gradient, altitude), **pressures** (pollution e.g. oil, litter, flow regulation, channel modification)]*	27% of England's freshwater bodies are currently classified as being of 'good status' or 'good ecological potential' or better (Environment Agency)<br><br>55% open water, 69% wetland, 81% lowland raised bog, 87% fen, marsh and swamp SSSIs favourable/ recovering (UK NEA, 2011)	All inland and coastal waters within defined river basin districts must reach at least good status by 2015 (WFD)<br><br>≥95% SSSI favourable/ recovering by 2020 (Biodiversity Strategy 2020)	1996-2007 plant species richness in ponds decreased by 20% and proportion of poor or very poor quality ponds increased by 17% (UK NEA, 2011).<br><br>Bird data is mixed - wet meadows declined, reed beds increased, slow/standing water increased, wetland birds declined with increasing severity in recent years (UK NEA, 2011)	**C**<br><br>(5)
			**C**<br>(-50% from target)		**B**<br>(-ve)	
			(2)		(3)	
Freshwater	Wildlife	Spatial configuration	27% of England's freshwater bodies are currently classified as being of 'good status' or 'good ecological potential' or better (Environment Agency)	All inland and coastal waters within defined river basin districts must reach at least good status by 2015 (WFD)	No trend information.	**C**<br><br>(8)
			**C**<br>(-50% from target)		**B**<br>(unknown)	
			(4)		(4)	
Urban	Clean water	Quantity	27% of England's freshwater bodies are currently classified as being of 'good status' or 'good ecological potential' or better (Environment Agency)<br><br>~10% of England's land area is classified as urban (UK NEA, 2011)	All inland and coastal waters within defined river basin districts must reach at least good status by 2015 (WFD)	Urban land cover projected to rise from 10.6% in 1991 to 11.9% in 2016 (SoNE, 2008)<br><br>Average projection for urban growth between 2011 and 2060 across UK is 3% (UKNEA. 2011)<br><br>Within London, the proportion of rivers and canals of 'good' chemical or	**C**<br><br>(7)

					biological status has more than doubled between 1990 and 2005 (ONS, 2007)	
			**C**<br>(-50% from target)		**A**<br>(+ve -ve)	
			(4)		(3)	

References:

Countryside Survey (2007) Countryside Survey: England Results 2007 Chapter 7 Mountain, Moor and Heath: Bracken, Dwarf Shrub Heath, Bog and Fen, Marsh and Swamp Broad Habitats http://www.countrysidesurvey.org.uk/sites/default/files/pdfs/reports2007/england2007/CS-England-Results2007-Chapter07.pdf]

Defra (2007) The Air Quality Strategy for England, Scotland, Wales and Northern Ireland https://www.gov.uk/government/uploads/system/uploads/attachment_data/file/69336/pb12654-air-quality-strategy-vol1-070712.pdf

Defra (2009) Safeguarding our Soils A Strategy for England. https://www.gov.uk/government/uploads/system/uploads/attachment_data/file/69261/pb13297-soil-strategy-090910.pdf]

Defra (2010) Charting Progress 2. The State of UK seas. UK Marine Monitoring and Assessment community

Defra (2011) Biodiversity 2020: A strategy for England’s wildlife and ecosystem services https://www.gov.uk/government/publications/biodiversity-2020-a-strategy-for-england-s-wildlife-and-ecosystem-services

Defra (2012) Agriculture in the UK. https://www.gov.uk/government/uploads/system/uploads/attachment_data/file/208436/auk-2012-25jun13.pdf

Defra, (2013a) Agriculture in the United Kingdom datasets. https://www.gov.uk/government/statistical-data-sets/agriculture-in-the-united-kingdom

Defra (2013b) Government Forestry and Woodlands Policy Statement https://www.gov.uk/government/uploads/system/uploads/attachment_data/file/221023/pb13871-forestry-policy-statement.pdf

DWI (2012) Drinking water 2012. A report by the Chief Inspector of drinking water http://dwi.defra.gov.uk/about/annual-report/2012/

EA (2008) Water resources in England and Wales - current state and future pressures http://webarchive.nationalarchives.gov.uk/20140328084622/http:/cdn.environment-agency.gov.uk/geho1208bpas-e-e.pdf

EA (2009) Corporate Strategy 2011-2015 Evidence: water and the water environment. http://www.environment-agency.gov.uk/static/documents/Research/Water_FINAL.pdf

EA (2013) Improving water quality https://www.gov.uk/government/policies/improving-water-quality

EEA (2013) Air Quality in Europe http://www.eea.europa.eu/publications/air-quality-in-europe-2013

EC (2008) Legislation: the Marine Directive http://ec.europa.eu/environment/marine/eu-coast-and-marine-policy/marine-strategy-framework-directive/index_en.htm

EC (2013) The Directive overview. Drinking Water Directive http://ec.europa.eu/environment/water/water-drink/legislation_en.html

EC (2014) The EU Water Framework Directive - integrated river basin management for Europe http://ec.europa.eu/environment/water/water-framework/index_en.html

Forestry Commission (2013) Forestry Statistics, 2013. http://www.forestry.gov.uk/newsrele.nsf/web-allbysubject/73409C3452A283B380257BF2002F2F2E

Hume, C. 2008. Wetland Vision Technical Document: overview and reporting of project philosophy and technical approach. The Wetland Vision Partnership. http://www.wetlandvision.org.uk/userfiles/File/Technical%20Document%20Website%20Version.pdf

IUCN (2012) UK Committee Peatlands Programme. Aletta Bonn Presentation at Bonn Climate Change Conference

JNCC (2013) Biodiversity Indicators http://jncc.defra.gov.uk/page-6891

Keep Britain Tidy (2013) Blue Flag beaches http://www.keepbritaintidy.org/blueflag/541

National Audit Office (2006). Enhancing green open space. Report by the Comptroller and Auditor General.

NE (2008) State of the Natural Environment http://publications.naturalengland.org.uk/publication/31043

NE (2010) Nature Nearby - Accessible Natural Greenspace Guidance (NE265) http://publications.naturalengland.org.uk/publication/40004

NE (2012) Agricultural Land Classification: protecting the best and most versatile agricultural land. Natural England Technical Information Note TIN049.

ONS (Office of National Statistics) (2007) Focus on London 2007. ONS, London.

ONS (2012) UK Environmental Accounts http://www.ons.gov.uk/ons/dcp171778_267211.pdf

Parliamentary Office of Science and Technology (2007) Postnote no 289 Urban Flooding.

UK National Ecosystem Assessment (2011) The UK National Ecosystem Assessment Technical Report. UNEP-WCMC, Cambridge. http://uknea.unep-wcmc.org/Resources/tabid/82/Default.aspx

UK National Ecosystem Assessment Follow-On (2014) Work Package Report 1: Annex 4 – Case studies http://uknea.unep-wcmc.org/LinkClick.aspx?fileticket=dNp%2bvIjmXi4%3d&tabid=82

Woodland Trust (2008) Woodland actions for biodiversity and their role in water management

Woodland Trust (2010) Space for People: Targeting action for woodland access. http://www.woodlandtrust.org.uk/publications/2010/07/space-for-people/
